# Dnmt3a overexpression disrupts skeletal muscle homeostasis, promotes an aging-like phenotype, and reduces metabolic elasticity

**DOI:** 10.1016/j.isci.2025.112144

**Published:** 2025-03-03

**Authors:** Mamoru Oyabu, Yuto Ohira, Mariko Fujita, Kiyoshi Yoshioka, Runa Kawaguchi, Atsushi Kubo, Yukino Hatazawa, Hinako Yukitoshi, Huascar Pedro Ortuste Quiroga, Naoki Horii, Fumihito Miura, Hiromitsu Araki, Masaki Okano, Izuho Hatada, Hitoshi Gotoh, Tatsuya Yoshizawa, So-ichiro Fukada, Yoshihiro Ogawa, Takashi Ito, Kengo Ishihara, Yusuke Ono, Yasutomi Kamei

**Affiliations:** 1 Graduate School of Life and Environmental Sciences, Kyoto Prefectural University, Kyoto 606-8522, Japan; 2 Institute for Research on Productive Aging (IRPA), Tokyo, Japan; 3 Laboratory of Stem Cell Regeneration and Adaptation, Graduate School of Pharmaceutical Sciences, Osaka University, 1-6 Yamadaoka, Suita, Osaka 565-0871, Japan; 4 Department of Muscle Development and Regeneration, Institute of Molecular Embryology and Genetics, Kumamoto University, Kumamoto 860-0811, Japan; 5 Department of Biochemistry, Kyushu University Graduate School of Medical Sciences, Fukuoka 812-8582, Japan; 6 Department of Pluripotent Stem Cell Biology, Institute of Molecular Embryology and Genetics, Kumamoto University, Kumamoto 860-0811, Japan; 7 Laboratory of Genome Science, Biosignal Genome Resource Center, Institute for Molecular and Cellular Regulation, Gunma University, Gunma 371-8512, Japan; 8 Viral Vector Core, Gunma University Initiative for Advanced Research (GIAR), Maebashi 371-8511, Japan; 9 Cell Biology, Graduate School of Medical Science, Kyoto Prefectural University of Medicine, Kyoto 606-0823, Japan; 10 Department of Medicine and Bioregulatory Science, Graduate School of Medical Sciences, Kyushu University, Fukuoka 812-8582, Japan; 11 Department of Food Science and Human Nutrition, Faculty of Agriculture, Ryukoku University, Shiga 520-2194, Japan; 12 Tokyo Metropolitan Institute for Geriatrics and Gerontology, Tokyo 173-0015, Japan

**Keywords:** Age, Epigenetics, Integrative aspects of cell biology, Model organism, Transcriptomics

## Abstract

Mammalian aging is reportedly driven by the loss of epigenetic information; however, its impact on skeletal muscle aging remains unclear. This study shows that aging mouse skeletal muscle exhibits increased DNA methylation, and overexpression of DNA methyltransferase 3a (Dnmt3a) induces an aging-like phenotype. Muscle-specific Dnmt3a overexpression leads to an increase in central nucleus-positive myofibers, predominantly in fast-twitch fibers, a shift toward slow-twitch fibers, elevated inflammatory and senescence markers, mitochondrial OXPHOS complex I reduction, and decreased basal autophagy. Dnmt3a overexpression resulted in reduced muscle mass and strength and impaired endurance exercise capacity with age, accompanied by an enhanced inflammatory signature. In addition, Dnmt3a overexpression reduced not only sensitivity to starvation-induced muscle atrophy but also the restorability from muscle atrophy. These findings suggest that increased DNA methylation disrupts skeletal muscle homeostasis, promotes an aging-like phenotype, and reduces muscle metabolic elasticity.

## Highlights

• Increased DNA methylation is a feature of aging mouse skeletal muscle
• Dnmt3a overexpression promotes aging-like phenotype in skeletal muscle
• Dnmt3a overexpression and aging synergistically increases the inflammatory signature
• Dnmt3a overexpression reduces muscle metabolic elasticity

## Graphical abstract

**Figure:**
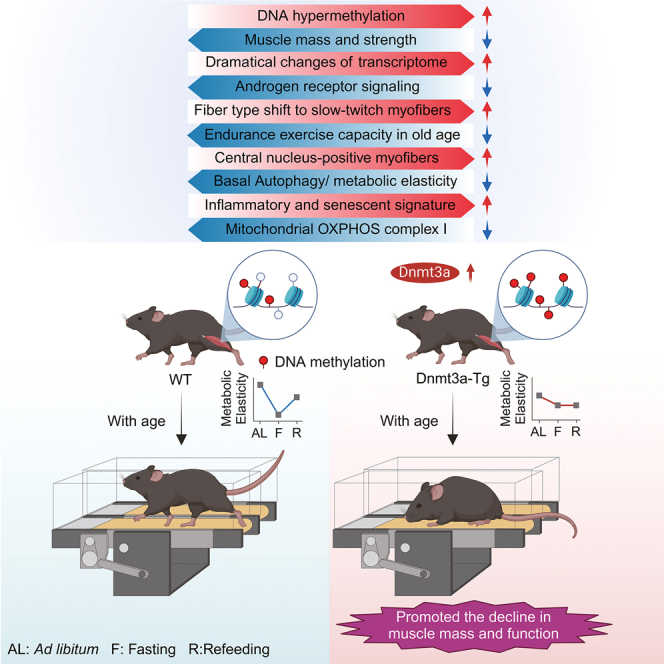

## Introduction

A decline in muscle mass and function is a feature of skeletal muscle aging, known as sarcopenia, which reduces activities of daily living and quality of life and increases the number of care-dependent individuals, number of hospitalizations, and rates of morbidity and mortality.^1^^,^^2^^,^^3^ Epigenetic modifications modify genes without changing the DNA sequence, determine tissue specificity, and maintain tissue homeostasis.^4^^,^^5^ Disruption of the epigenome is one of the hallmarks of aging,^6^ and its imbalance causes a wide range of diseases and loss of identity of multiple organs.^7^^,^^8^ DNA methylation, one of the most tightly regulated epigenetic modifications, is closely linked to mammalian aging and maximum lifespan across species.^9^^,^^10^^,^^11^^,^^12^^,^^13^^,^^14^ DNA methylation state at CpG sites is a potential biomarker for predicting biological age in skeletal muscle,^8^^,^^15^ and DNA methylation in skeletal muscle has been reported to increase with age.^16^^,^^17^^,^^18^^,^^19^ Other studies have demonstrated the hypermethylation in promoter regions in aging skeletal muscle.^15^^,^^20^ In contrast, positive stimuli such as exercise reduce DNA methylation in skeletal muscle. For example, acute^21^^,^^22^ and chronic aerobic exercise in humans,^23^^,^^24^^,^^25^ acute and chronic resistance exercise in humans,^26^^,^^27^^,^^28^ acute mechanical overload^29^ and chronic progressive weighted wheel running (PoWeR) in mice,^30^^,^^31^ and acute and chronic high-intensity exercise in humans^32^^,^^33^ have all been reported to hypomethylate the skeletal muscle methylome, although the DNA hypomethylation occurs in different regions depending on the type of exercise. Methylation of genomic DNA is regulated by DNA methyltransferases (Dnmts), which are classified into *de novo* methyltransferase (Dnmt3a and Dnmt3b) and maintenance methyltransferase (Dnmt1).^34^ Contrary to the fact that aging seems to increase DNA methylation in skeletal muscle and exercise rejuvenates DNA methylome in skeletal muscle,^18^^,^^19^^,^^35^^,^^36^^,^^37^ transient overexpression of muscle Dnmt3a, responsible for increasing DNA methylation, could suppress denervation- and diabetes-induced muscle atrophy.^38^^,^^39^ Moreover, Dnmt3a deficiency suppressed muscle satellite cell proliferation and reduced muscle regeneration.^40^^,^^41^ After endurance exercise, endogenous Dnmt3a increased in slow-twitch muscle to prevent reactive oxygen species production, promoting tolerance to endurance exercise.^42^ A missense mutation in Dnmt3a was associated with a congenital myopathy with rhabdomyolysis.^43^ These studies indicate that Dnmt3a is a potential target for treating muscle atrophy and muscle disease, because it (1) confers resistance to acute muscle atrophy and (2) plays an important role in muscle regeneration and exercise adaptation. However, the effect of chronic increase in DNA methylation by Dnmt3a on muscle homeostasis is unknown. Moreover, whether chronic increase in DNA methylation is sufficient to affect skeletal muscle aging, skeletal muscle mass, and function is unknown.

Recent advances in research focusing on skeletal muscle aging have shown that aging skeletal muscle is characterized by upregulation of several senescence marker genes, including *Cdkn1a/p21*^*CIP1*^ and *Cdkn2a/p16*^*Ink4a*^, and by inflammation associated with aging (“inflammaging”^44^), caused, at least in part, by the secretion of pro-inflammatory cytokines and chemokines by senescent cells, known as the senescence-associated secretory phenotype (SASP).^45^^,^^46^^,^^47^ In addition, aging skeletal muscle is characterized by enhanced immune cell infiltration, and enrichment of immune-related pathways, such as complement cascade and coagulation.^48^^,^^49^ Another hallmark of skeletal muscle aging is dysregulated muscle mitochondria.^47^^,^^50^^,^^51^^,^^52^ However, the underlying causes of the hallmarks of skeletal muscle aging, in particular the impact of epigenetic changes on muscle senescence-like changes and “inflammaging,” remain to be elucidated.

In this study, we investigated what changes occur in skeletal muscle when epigenomic information is perturbed by the approach of increased DNA methyltransferase (overexpression of Dnmt3a). We show that sustained Dnmt3a overexpression in skeletal muscle increases DNA methylation, disrupts muscle homeostasis, and causes aging and myopathy-like phenotype in skeletal muscle. Dnmt3a overexpression in skeletal muscle resulted in increased inflammatory and senescent signatures in skeletal muscle, shift to slow myofibers (occurs in sarcopenia), decreased androgen receptor (AR) signaling, decreased mitochondrial OXPHOS complex I protein level, low basal autophagy, and fast myofiber-specific muscle atrophy. Furthermore, sustained Dnmt3a overexpression augmented inflammatory signature in a manner that is enhanced with age, leading to promoted age-related muscle atrophy and reduced endurance exercise capacity with age. In addition, overexpression of Dnmt3a in the postnatal myofibers using the myotropic AAV (MyoAAV) also induced skeletal muscle atrophy with similar molecular changes to the Dnmt3a-Tg model. These data suggest that a balance of DNA methylation is important for maintaining skeletal muscle homeostasis and identity and that increased DNA methylation leads to skeletal muscle atrophy.

## Results

### Dnmt3a overexpression increases DNA methylation, dramatically alters the transcriptome, and results in reduced fast-twitch muscle mass

**Figure 1:**
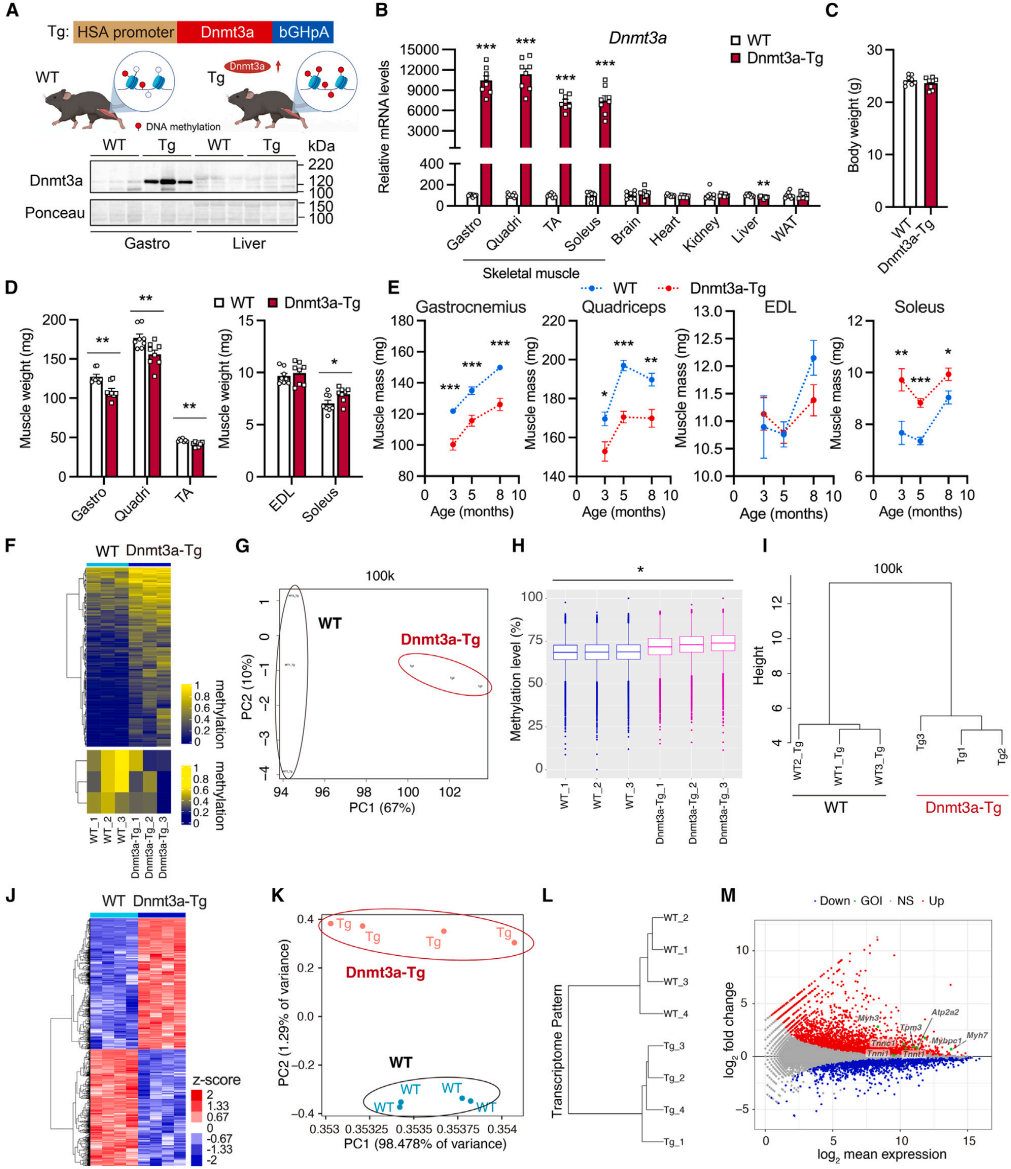
Sustained Dnmt3a overexpression increases DNA methylation, dramatically changes the transcriptome, and results in reduction of fast-twitch muscle mass (A) Schematic representation of Dnmt3a transgenic experimental systems and immunoblot of Dnmt3a levels in the gastrocnemius muscle and liver of WT and Dnmt3a-Tg mice (*n* = 3 mice/group). (B) Quantitative RT-PCR of Dnmt3a mRNA in the skeletal muscle (gastrocnemius, quadriceps, tibialis anterior [TA], and soleus) and other tissues (brain, heart, kidney, liver, and white adipose tissue [WAT]) in female WT and Dnmt3a-Tg mice (*n* = 8 mice/group, except for heart where WT; *n* = 8, Dnmt3a-Tg; *n* = 6). (C) Body weight of male 3-month-old WT and age-matched Dnmt3a-Tg mice (*n* = 8 mice/group). (D) Weights of skeletal muscles (gastrocnemius [Gastro], quadriceps [Quadri], tibialis anterior [TA], extensor digitorum longus [EDL], and soleus [Soleus]) from male 3-month-old WT and age-matched Dnmt3a-Tg mice (*n* = 8 mice/group). (E) Weights of skeletal muscles (gastrocnemius, quadriceps, extensor digitorum longus [EDL], and soleus [Soleus]) at the age of indicated months (n = 8–12 male mice/group). (F) Heatmap showing hypermethylated and hypomethylated DMRs in TA muscle from Dnmt3a-Tg mice compared with WT mice, identified using post-bisulfite adaptor-tagging (PBAT)-mediated targeted methylome sequencing (*n* = 3 mice/group). (G) Principal-component analysis of DNA methylation of 100-kb sidling windows (*n* = 3 mice/group). (H) Boxplot of mean CpG methylation level of 100-kb sidling windows (*n* = 3 mice/group). Significant differences in median DNA methylation levels are shown as ∗*p* < 0.05. (I) Unsupervised hierarchical clustering using Euclidean distance across the sample set (*n* = 3 mice/group). (J) Hierarchical clustering of differentially expressed genes (DEGs) in young (3-month-old) Dnmt3a-Tg muscle with FDR <0.05 compared to age-matched WT muscle (*n* = 4 mice/group). (K) Principal-component analysis of gene expression profile from young (3-month-old) WT and Dnmt3a-Tg muscles (*n* = 4 mice/group). (L) Hierarchical clustering of transcriptome pattern across the sample set from young (3-month-old) WT and Dnmt3a-Tg muscles (*n* = 4 mice/group). (M) MA plot showing gene expression changes from young (3-month-old) WT and Dnmt3a-Tg muscles (*n* = 4 mice/group). Red and blue plots show significantly increased and decreased genes, respectively, with FDR <0.05. GOI, genes of interest; NS, not significant. All data indicate mean ± SE. ∗*p* < 0.05, ∗∗*p* < 0.01, ∗∗∗*p* < 0.001. (B–E and H) Student’s two-tailed unpaired t test. WT, wild type; Tg, Dnmt3a-Tg.

In skeletal muscle, DNA methylation increases with age.^16^^,^^17^^,^^18^^,^^19^ To mimic the chronic increase of DNA methylation, we generated mice overexpressing Dnmt3a specifically in skeletal muscle under the control of human skeletal α-actin promoter (Dnmt3a-Tg mice) (Figure 1A). Quantitative reverse transcription PCR (RT-qPCR) and western blot analysis confirmed Dnmt3a overexpression only in skeletal muscles, including gastrocnemius, quadriceps, tibialis anterior (TA), and soleus (slow-twitch muscle) (Figures 1A and 1B). Although Dnmt3a-Tg mice had normal body weight, glucose tolerance, insulin sensitivity, and comparable sensitivity to high-fat-diet-induced obesity with wild-type (WT) mice (Figures 1C and S1A–S1C), their gastrocnemius, quadriceps, and TA muscle (fast-twitch muscle) mass were smaller (similar to that in patients with sarcopenia); conversely, soleus muscle mass was larger in Dnmt3a-Tg mice (Figure 1D). These robust changes in muscle mass of Dnmt3a-Tg mice were observed not only at 3 months but also at 5 and 8 months of age (Figure 1E).

To examine whether overexpressed Dnmt3a is functional in Dnmt3a-Tg mice, we analyzed the DNA methylome of gastrocnemius muscles harvested from WT and Dnmt3a-Tg mice using microarray-based integrated analysis of methylation by isoschizomers (MIAMI).^53^ Several genes were hypermethylated in Dnmt3a-Tg mice (Figure S1D). To evaluate the features of differentially methylated genes (DMGs) hypermethylated by Dnmt3a, we performed gene ontology (GO) enrichment analysis. Enrichment in cellular component terms “neuronal cell body,” “neuromuscular junction,” and “sarcomere” (Figure S1E) indicate Dnmt3a-mediated hypermethylation of predominantly skeletal-muscle-related genes. GO enrichment analysis of biological process and molecular function, as well as protein-protein interaction (PPI) networks, using the STRING database^54^ showed enrichment of genes associated with neuromuscular junction, myogenesis, and PI3K-Akt signaling (Figures S1F–S1I). We performed post-bisulfite adaptor-tagging (PBAT)-mediated methylome sequencing^55^^,^^56^ using TA muscle from WT and Dnmt3a-Tg mice. Heatmap analysis of differentially methylated regions (DMRs), principal component analysis, analysis of methylation status at CpG sites, and unsupervised hierarchical clustering clearly separated the methylation patterns between WT and Dnmt3a-Tg muscles and indicated that DNA methylation was relatively higher in Dnmt3a-Tg muscle than WT muscle (Figures 1F–1I and S1J–S1L). Increased levels of DNA methylation in skeletal muscle of Dnmt3a-Tg mice were confirmed by comparing DNA methylation changes not only in the 100 kb window but also in the 5 kb window (Figures S1J–S1L). Thus, overexpressed Dnmt3a is functional in Dnmt3a-Tg mice. As epigenetic changes could affect the gene expression profile, we next performed microarray analysis of WT and Dnmt3a-Tg muscles to evaluate Dnmt3a-expression-mediated changes in the transcriptome. Heatmap analysis revealed a number of differentially expressed genes (DEGs) in Dnmt3a-Tg muscle with a false discovery rate (FDR) < 0.05 compared to WT muscle (Figure 1J). Principal-component analysis and hierarchical clustering clearly separated the transcriptome patterns between WT and Dnmt3a-Tg muscles and indicated that transcriptome was dramatically altered in Dnmt3a-Tg muscle (Figures 1K and 1L). Given the nature of transcriptome analysis, where genes with high expression tend to show a narrower range of expression variation, we generated an MA plot with a criterion of FDR <0.05. The MA plot showed that Dnmt3a expression significantly altered the expression of genes, including relatively high basal expression in skeletal muscle, such as muscle structural genes encoding myosin heavy chain and troponin (Figure 1M). The DMGs or DMR-associated genes (DMRGs) induced by Dnmt3a expression marginally overlapped with the DEGs (Figures S1M and S1N, Table S1). We speculate that in skeletal muscle, increased DNA methylation leads to the erosion of the epigenetic landscape, which indirectly affects gene expression profiles and some phenotypic changes.

### Dnmt3a overexpression results in dramatic fiber-type shift to slow-twitch myofibers

**Figure 2:**
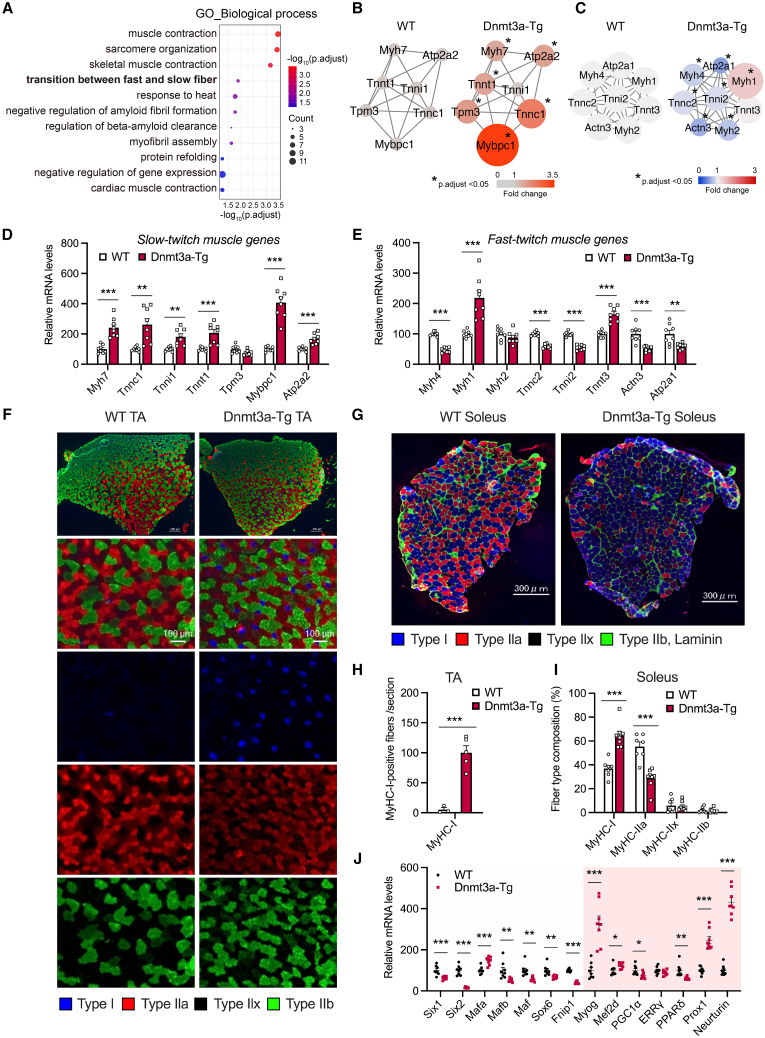
Sustained Dnmt3a overexpression results in dramatic fiber-type shift to stress-resistant type I myofibers (A) GO enrichment analysis in biological process of DEGs with relatively high basal expression (genes presumably expressed in myofibers), with increased (fold change >1.5, FDR <0.05) expression in gastrocnemius muscle from Dnmt3a-Tg mice compared with WT mice. (B and C) Protein-protein interaction (PPI) network from microarray data illustrating a set of slow-twitch myofiber-specific genes was upregulated (B), and conversely fast-twitch myofiber-specific genes were downregulated (C) in Dnmt3a-Tg muscle compared with WT muscle. Color and circle size of nodes indicate fold-change values (*n* = 4 mice/group). (D and E) Relative mRNA expression of slow-twitch muscle genes (D) and fast-twitch muscle genes (E) in gastrocnemius muscle from WT and Dnmt3a-Tg mice (*n* = 8 male mice/group). (F) Representative images of immunohistochemical staining of the myosin heavy chains (type I, blue; type IIa, red; type IIb, green) in TA muscle cross-sections of WT and Dnmt3a-Tg mice. Unstained fibers are considered type IIx fibers (black). Scale bar, 300 μm (top) and 100 μm. (G) Representative images of immunohistochemical staining of the myosin heavy chains and laminin (type I, blue; type IIa, red; type IIb and laminin, green) in soleus muscle cross-sections of WT and Dnmt3a-Tg mice. Unstained fibers are considered type IIx fibers (black). Scale bar, 300 μm. (H) Quantification of type I myofibers of TA muscle sections of WT and Dnmt3a-Tg mice (*n* = 3 WT mice, *n* = 5 Dnmt3a-Tg mice). (I) Frequency of each fiber type in the soleus muscle from WT and Dnmt3a-Tg mice (n = 7–8 mice/group). (J) Relative mRNA expression of the key regulators of muscle fiber type determination in gastrocnemius muscle from WT and Dnmt3a-Tg mice (*n* = 8 male mice/group). All data indicate mean ± SE. ∗*p* < 0.05, ∗∗*p* < 0.01, ∗∗∗*p* < 0.001. (B–E, H–J) Student’s two-tailed unpaired t test.

To evaluate phenotypic changes due to increased DNA methylation, we examined changes in the skeletal muscle transcriptome in Dnmt3a-Tg mice. We identified genes with relatively high basal expression (genes presumably expressed in myofibers) that characterize the Dnmt3a-Tg muscle. In total, 169 DEGs had increased (fold change >1.5) expression with a criterion of FDR <0.05 in Dnmt3a-Tg muscle (Table S2). GO enrichment analysis of biological process showed enrichment in genes associated with muscle fiber type switching and muscle development, including “muscle contraction,” “sarcomere organization,” and “transition between fast and slow fiber” (Figure 2A). GO enrichment analysis of molecular function and cellular component exhibited enrichment in muscle-development-associated genes, including “actin filament binding,” “actin binding,” “Z disc,” “M band,” and “I band” (Figures S2A and S2B). Kyoto Encyclopedia of Genes and Genomes (KEGG) pathway analysis showed that Dnmt3a-Tg muscle is enriched in pathways associated with cardiomyopathy (Figure S2C). Previous studies have shown that aging human skeletal muscle has an increased proportion of slow-twitch (type I, Myh7^+^) myofibers.^48^^,^^49^ Network analysis showed upregulation of a set of slow-twitch myofiber-specific genes and downregulation of fast-twitch myofiber-specific genes in Dnmt3a-Tg muscle compared with WT muscle (Figures 2B and 2C). RT-qPCR analysis confirmed that the expression of type I muscle-fiber-related genes, such as *Myh7*, *Tnnc1*, *Tnni1*, *Tnnt1*, *Mybpc1*, and *Atp2a2/SERCA2*, significantly increased (Figure 2D), whereas that of type II muscle-fiber-related genes, such as *Myh4*, *Tnnc2*, *Tnni2*, *Actn3*, and *Atp2a1/SERCA1*, significantly decreased (Figure 2E) in Dnmt3a-Tg muscle compared with WT muscle. TA rarely has type I myofibers; however, immunohistochemical analysis showed that Dnmt3a expression dramatically increased the formation of slow-twitch type I myofibers in TA (fast-twitch) and soleus (slow-twitch) muscles (Figures 2F–2I and S2D–S2I), with altered expression of muscle fiber type determining factors^57^^,^^58^^,^^59^^,^^60^^,^^61^^,^^62^^,^^63^^,^^64^^,^^65^^,^^66^^,^^67^^,^^68^^,^^69^ (Figure 2J). To test the hypothesis that a proportion of type I myofibers increased in Dnmt3a-Tg mice due to differentiation of muscle satellite cells into type I myofibers, we induced muscle damage by injecting cardiotoxin (CTX) into TA. Immunohistochemical staining showed that the Dnmt3a-Tg muscle contained a remarkably larger number of type I myofibers than WT muscle; however, CTX injection did not increase the number of type I muscle fibers in WT or Dnmt3a-Tg mice (Figures S3A–S3C). Thus, type I myofiber formation in Dnmt3a-Tg mice is likely independent of muscle satellite cells. To evaluate the effects of Dnmt3a overexpression on muscle morphology and gene expression, we collected satellite cells from extensor digitorum longus (EDL) and soleus muscles of C57BL/6J mice and generated primary myotubes with Dnmt3a overexpression using a retrovirus *in vitro*. Dnmt3a overexpression resulted in myotube atrophy in EDL-derived primary myotubes; Dnmt3a increased type I myosin heavy chain gene (*Myh7*) expression and decreased type II myosin heavy chain gene (*Myh2*, *Myh1*, and *Myh4*) expression (Figures S4A–S4D). Slow-twitch muscles rely on oxidative metabolism, using fatty acids as fuel source.^70^ Because the number of type I muscle fibers increased in Dnmt3a-Tg mice, we measured oxygen consumption in WT and Dnmt3a-Tg mice. However, the rate of oxygen consumption did not increase in Dnmt3a-Tg mice (Figure S4E). Furthermore, respiratory exchange ratio (RER; VCO_2_/VO_2_) did not decrease (Figure S4F), and fat oxidation did not increase in Dnmt3a-Tg mice (Figure S4G). These results indicate that energy expenditure and fat oxidation are not coupled in Dnmt3a-Tg mice, despite the increase in type I myofibers. Slow-twitch muscles can support endurance exercise because they possess a large number of mitochondria.^70^ We measured mitochondrial content and activity in Dnmt3a-Tg muscles. Mitochondrial DNA content relative to nuclear DNA content (COX2/COX4 ratio) was not different in young (3-month-old) WT and Dnmt3a-Tg muscles, suggesting no change in the number of mitochondrial copies (Figure S4H). The activity of succinate dehydrogenase, a key enzyme of the TCA cycle and electron transport chain, was not different between young (3-month-old) Dnmt3a-Tg and WT muscles, suggesting no change in oxidative capacity based on mitochondrial complex II (Figure S4I). In addition, the endurance exercise capacity of young (7-week-old) Dnmt3a-Tg mice was not significantly different from that of age-matched WT mice, although a trend toward decreased endurance exercise capacity was observed in Dnmt3a-Tg mice (Figure S4J). Taken together, these results suggest that increase in Dnmt3a-mediated DNA methylation dramatically shifts the muscle fiber type from fast- to slow-twitch myofibers without increasing the mitochondrial content and endurance exercise capacity.

### Dnmt3a overexpression disrupts skeletal muscle homeostasis and results in a fast myofiber-specific muscle atrophy with increased inflammatory and senescent signatures

**Figure 3:**
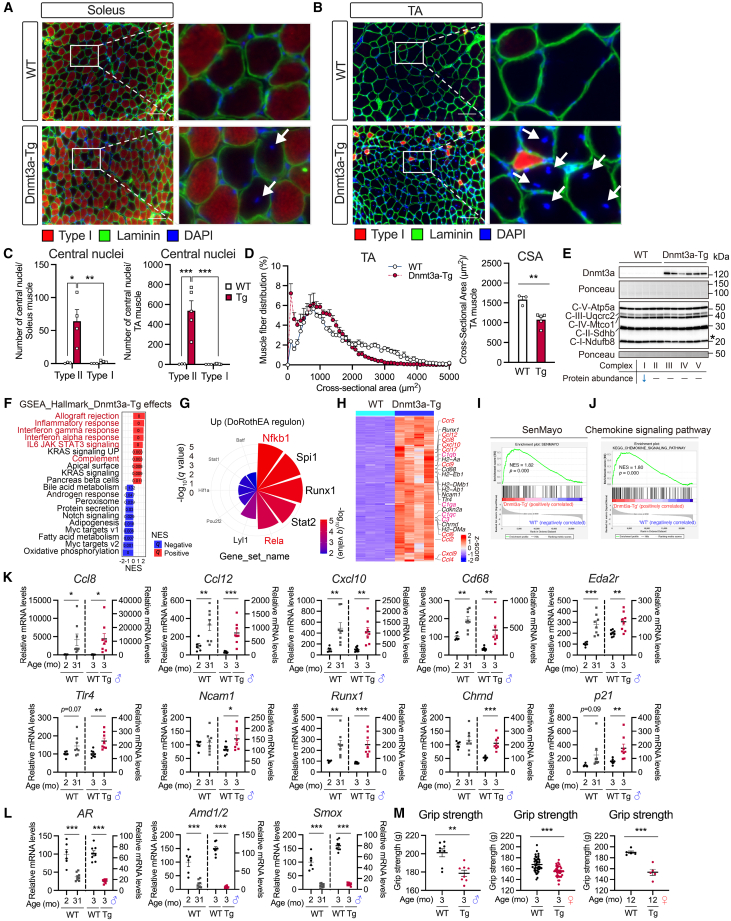
Sustained Dnmt3a overexpression results in myopathy-like phenotype with increased inflammatory and senescent signatures (A and B) Representative images of immunohistochemical staining of myosin heavy chains, laminin, and DAPI (type I, red; laminin, green and DAPI, blue) in soleus (A) and TA (B) muscle cross-section of WT and Dnmt3a-Tg mice. Scale bar, 100 μm. White arrows indicating observed central nuclei in a muscle section of Dnmt3a-Tg mice. (C) Quantification of central nuclei of soleus (left) and TA (right) muscle section of WT and Dnmt3a-Tg mice (*n* = 3 WT mice, n = 4–5 Dnmt3a-Tg mice). (D) Muscle fiber cross-sectional area (CSA) distribution (left) and average values (right) in TA muscle sections of WT and Dnmt3a-Tg mice (*n* = 3 WT mice, *n* = 5 Dnmt3a-Tg mice). (E) Immunoblot of Dnmt3a and OXPHOS complex protein levels in the TA muscle of young (3-month-old, female) WT and Dnmt3a-Tg mice (*n* = 6 mice/group). (F) GSEA of Dnmt3a-regulated genes from microarray data of young (3-month-old) WT and Dnmt3a-Tg muscles (*n* = 4 mice/group) using gene sets of the Molecular Signatures Database (MSigDB) “hallmark gene sets.” Red and blue bar show positive and negative normalized enrichment score (NES), respectively, and FDR *q* values were shown in each bar. (G) Enrichment analysis using DoRothEA regulon gene sets, showing the top 10 putative central transcription factors that are positively regulated in the transcriptome of Dnmt3a-Tg muscle. (H) Heatmap showing differentially expressed genes (DEGs) increased in young (3-month-old) Dnmt3a-Tg muscle with FDR <0.05 and fold-change value >2 compared to age-matched WT muscle (*n* = 4 mice/group). Genes of interest were shown in the figure. (I and J) GSEA of gene sets of (I) SenMayo and (J) chemokine signaling pathway from microarray data of young (3-month-old) WT and Dnmt3a-Tg muscles (*n* = 4 mice/group). (K and L) Relative mRNA expression of chemokine-related genes, immune-system-related genes, denervation marker genes, a senescent cell marker gene (K), androgen receptor (AR), and polyamine-metabolism-related genes (L) in gastrocnemius muscle from 3-month-old male WT and Dnmt3a-Tg mice (*n* = 8 mice/group) and gastrocnemius muscle from young (3-month-old) and super-aged (31-month-old) male C57BL/6J mice (*n* = 6 young mice, *n* = 8 super-aged mice). (M) Grip strength in WT and Dnmt3a-Tg mice (3-month-old male; *n* = 8 mice/group, 3-month-old female; *n* = 34–37 mice/group, and 12-month-old female; *n* = 5 mice/group). All data indicate mean ± SE. ∗*p* < 0.05, ∗∗*p* < 0.01, ∗∗∗*p* < 0.001. (D, E, K, L, and M) Student’s two-tailed unpaired t test. (C) Two-way analysis of variance (ANOVA) followed by Tukey’s *post hoc* test. WT: wild type; Tg: Dnmt3a-Tg.

DNA methylation is tightly controlled to determine tissue specificity and maintain homeostasis.^4^^,^^5^ An imbalance in DNA methylation causes a wide range of diseases.^7^ Skeletal muscle Dnmt3a deletion did not affect muscle fiber type in our muscle-specific Dnmt3a knockout mouse model (Figures S5A and S5B) or other studies.^42^ Slow-twitch myofibers are more resistant to disruptions in muscle homeostasis than fast-twitch myofibers.^71^ We hypothesized that an increase in Dnmt3a-mediated DNA methylation disrupts skeletal muscle homeostasis, resulting in a dramatic increase in stress-resistant slow-twitch myofibers, even in TA. We performed immunohistochemical analysis to test this hypothesis. Nuclear dislocation, a feature of dysfunctional myofibers in various muscle disorders, as well as regenerative myofibers, hinders muscle contraction.^72^ Aging skeletal muscle also shows an increase in degenerative myofibers with nuclear dislocation.^45^^,^^73^^,^^74^ Our results revealed that Dnmt3a-Tg muscle had increased myofiber damage, evidenced by an increased number of central nuclei in both soleus and TA muscles (Figures 3A and 3B). Interestingly, the central-nucleus-positive damaged fibers were predominantly observed in fast-twitch myofibers (Figure 3C), suggesting that fast-twitch myofibers were more sensitive to muscle damage caused by increased DNA methylation and the fibers may have changed to stress-resistant slow-twitch myofibers as a means of adaptation. Measurement of skeletal muscle cross-sectional area showed that Dnmt3a-Tg muscle had a dramatically increased frequency of small myofibers and loss of large myofibers in the TA muscle (Figure 3D), indicating that Dnmt3a-mediated increase in DNA methylation results in muscle atrophy. As aging skeletal muscle is characterized by dysregulated muscle mitochondria,^47^^,^^50^^,^^51^ we measured the abundance of mitochondrial OXPHOS complex proteins and found that OXPHOS complex I protein abundance was specifically reduced in young (3-month-old) Dnmt3a-Tg muscle compared with age-matched WT muscle (Figures 3E and S5C).

To further evaluate disrupted skeletal muscle homeostasis induced by increased DNA methylation, we examined the changes in the skeletal muscle gene expression, including those with relatively low basal expression (expressed in all cell types; not only myofibers) of Dnmt3a-Tg muscle. Gene set enrichment analysis (GSEA) using all datasets from WT and Dnmt3a-Tg muscles showed that enrichment was increased in gene sets associated with the inflammation and abnormal immune response, including “allograft rejection,” “inflammatory response,” “interferon gamma response,” “interferon alpha response,” “IL6 JAK STAT3 signaling,” and “complement” (Figures 3F and S6A). In addition, transcription factor (TF) enrichment analysis based on the DoRothEA regulon gene set^75^ identified well-known inflammation-associated nuclear factor κB (NF-κB) subunits, including Nfkb1 and RelA (Figures 3G, S6B, and S6C), which are associated with transcriptional activation of SASP factors.^76^ We identified 1,571 DEGs with increased (fold change >2) expression in Dnmt3a-Tg muscle, including upregulation of genes associated with the senescence (*Cdkn2a/p16*^*Ink4a*^) and chemokines (Figure 3H, Table S3). Recent research has established a new gene set (SenMayo) that monitors the burden of senescent cells in aging and other conditions.^77^ GSEA of this senescence-associated gene set (SenMayo), which is predominantly composed of SASP factors (66.4%), showed that the senescent signature was increased in Dnmt3a-Tg muscle compared to WT muscle (Figures 3I and S6D). Dnmt3a-Tg muscle was also highly enriched for the chemokine signaling pathway (Figures 3J and S6E), consistent with a hallmark of aging skeletal muscle.^45^ RT-qPCR analysis confirmed that chemokine- (*Ccl8*, *Ccl12*, and *Cxcl10*) and immune-system- (*Cd68*, *Eda2r*, and *Tlr4*) related genes were upregulated in Dnmt3a-Tg muscle (Figure 3K). In addition, the expression of denervation markers (*Ncam1*, *Runx1*, and *Chrnd*)^78^^,^^79^ and a senescent cell marker (*Cdkn1a/p21*^*CIP1*^)^80^^,^^81^ increased in Dnmt3a-Tg muscle (Figure 3K). Similar transcript changes were observed in the aging skeletal muscle of super-aged 31-month-old C57BL/6J mice, which showed a dramatic loss of muscle mass even when compared to immature 2-month-old C57BL/6J mice (Figures 3K and S6F). The loss of muscle mass and the upregulation of chemokine-related genes (*Ccl8*, *Ccl12*, and *Cxcl10*) in Dnmt3a-Tg muscle were partially attenuated by 5-month resistance exercise based on ladder climbing, suggesting that resistance exercise could attenuate DNA-hypermethylation-induced muscle inflammatory signaling (Figures S7A and S7B). Decreased androgen receptor (AR) signaling in skeletal muscle leads to impaired muscle function and sarcopenia-like phenotype.^82^^,^^83^ Androgen response was negatively enriched in Dnmt3a-Tg muscle with negative normalized enrichment score (NES) (Figure 3F), and muscle *AR* gene expression was downregulated in skeletal muscles of both Dnmt3a-Tg and super-aged 31-month-old C57BL/6J mice (Figure 3L). Evidence suggests that expression of polyamine metabolic enzymes is regulated by androgen signaling in skeletal muscle.^83^^,^^84^^,^^85^ Network analysis demonstrated that a set of genes associated with polyamine metabolism were downregulated in Dnmt3a-Tg muscle compared with WT muscle (Figure S7C). RT-qPCR analysis confirmed that expression of polyamine-metabolism-related genes (*Amd1/2* and *Smox*) and a muscle-strength-associated AR target gene (*Mylk4*)^82^ significantly decreased in Dnmt3a-Tg muscle, similar to that seen in super-aged 31-month-old C57BL/6J muscle (Figures 3L and S6F). Because decreased muscle androgen signaling is associated with impaired muscle strength,^82^ we examined change in muscle strength in Dnmt3a-Tg mice. Our results revealed that Dnmt3a-Tg mice had significantly decreased muscle strength (Figure 3M). This muscle weakness was observed not only in males but also in female Dnmt3a-Tg mice (Figure 3M). Transcriptome analysis showed that the overall gene expression profile of 3-month-old Dnmt3a-Tg muscle correlated more closely with that of the 26-month-old WT muscle than the 3-month-old WT muscle (Figures S7D and S7E). Taken together, these results suggest that increase in Dnmt3a-mediated DNA methylation disrupts muscle homeostasis, resulting in a fast myofiber-specific muscle atrophy with increased inflammatory and senescent signatures in skeletal muscle.

### Sustained Dnmt3a overexpression promotes the age-related loss of muscle mass and impairs endurance exercise capacity with augmented muscle inflammatory signature with age

**Figure 4:**
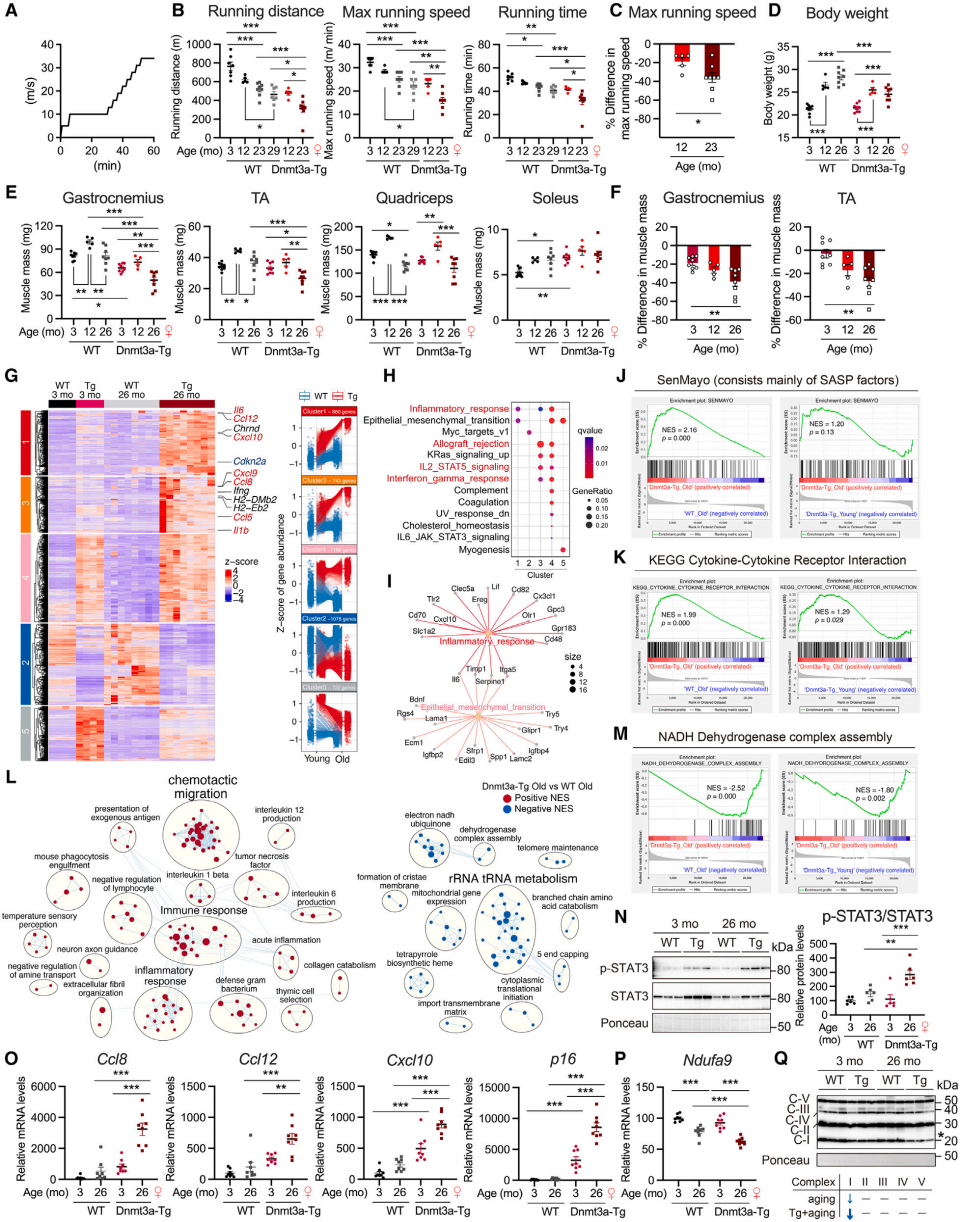
Sustained Dnmt3a overexpression exacerbates loss of muscle mass and function during aging with augmented muscle inflammatory signature with age (A) Protocol of the treadmill running performance test. (B) Total running distance and time, as well as peak speed, achieved by 3-, 12-, 23-, and 29-month-old WT and 12- and 23-month-old Dnmt3a-Tg female mice in a treadmill running test (n = 5–8 mice/group). (C) The percentage reduction in maximal running speed of Dnmt3a-Tg mice relative to WT mice at 12 and 23 months of age (n = 5–8 mice/group). (D) Body weights of 3-, 12-, and 26-month-old WT and Dnmt3a-Tg female mice (*n* = 8 mice/group). (E) Weights of skeletal muscles (gastrocnemius, tibialis anterior [TA], quadriceps, and soleus) from 3-, 12-, and 26-month-old WT and Dnmt3a-Tg female mice (n = 5–8 mice/group). (F) The percentage reduction in gastrocnemius and TA muscle mass of Dnmt3a-Tg mice relative to WT mice at 3, 12, and 26 months of age (n = 5–8 mice/group). (G) Heatmap analysis using k-means clustering (left) and boxplot (right) of differentially expressed genes (DEGs) in older (26-month-old) Dnmt3a-Tg muscle with FDR <0.05 and fold-change value > 1.5 compared to older (26-month-old) WT muscle. Multi-DEG analysis in young (3-month-old) and older (26-month-old) WT and age-matched Dnmt3a-Tg muscles was performed using RNAseqChef (3-month-old WT; *n* = 4, 3-month-old Dnmt3a-Tg; *n* = 4, 26-month-old WT; *n* = 8, 26-month-old Dnmt3a-Tg; *n* = 8). Genes of interest were shown in the figure. (H) Enrichment analysis based on MSigDB hallmark using DEGs classified into each cluster in Figure 4G. (I) Cnet plots of DEGs classified into cluster 1 in Figure 4G. (J and K) GSEA of gene sets of (J) SenMayo and (K) cytokine-cytokine receptor interaction from microarray data where older (26-month-old) Dnmt3a-Tg muscle was compared to older (26-month-old) WT and young (3-month-old) Dnmt3a-Tg muscle. (L) Clusters of gene sets (GSEA’s results) differentially enriched in older (26-month-old) Dnmt3a-Tg muscle compared to older (26-month-old) WT muscle with FDR <0.05 and the Jaccard overlap combined coefficient >0.375 with combined constant = 0.5. Red and blue nodes represent positive and negative normalized enrichment score (NES), and circle size of nodes were mapped to gene set size. (M) GSEA of a gene set of NADH dehydrogenase complex assembly from microarray data where older (26-month-old) Dnmt3a-Tg muscle was compared to older (26-month-old) WT and young (3-month-old) Dnmt3a-Tg muscle. (N) The representative immunoblot image of p-STAT3 and STAT3 protein levels in the TA muscle of young (3-month-old, female) and old (26-month-old, female) WT and Dnmt3a-Tg mice (*n* = 6 mice/group). (O and P) Relative mRNA expression of (O) chemokine-related genes, a senescent cell marker gene, and (P) NADH dehydrogenase complex gene in gastrocnemius muscle from young (3-month-old) and older (26-month-old) female WT and Dnmt3a-Tg mice (*n* = 8 mice/group). (Q) The representative immunoblot image of OXPHOS complex protein levels in the TA muscle of young (3-month-old, female) and old (26-month-old, female) WT and Dnmt3a-Tg mice (*n* = 6 mice/group). All data indicate mean ± SE. ∗*p* < 0.05, ∗∗*p* < 0.01, ∗∗∗*p* < 0.001. (C) Student’s two-tailed unpaired t test. (B, D, E, F, N, O, P and Q) One-way analysis of variance (ANOVA) followed by Tukey’s *post hoc* test. WT, wild type; Tg, Dnmt3a-Tg.

Since DNA methylation in human and rodent skeletal muscle has been reported to increase with age,^16^^,^^17^^,^^18^^,^^19^ we investigated the effect of chronic increases in DNA methylation by Dnmt3a expression on skeletal muscle aging. The endurance exercise capacity declines with age. The results of the treadmill running test (Figure 4A) showed that the endurance exercise capacity of WT mice declined significantly with age (Figure 4B). The 12-month-old Dnmt3a-Tg mice had a similar endurance exercise capacity as the 23- to 29-month-old WT mice (Figure 4B). The 23-month-old Dnmt3a-Tg mice had a significantly impaired endurance exercise capacity compared to the age-matched (23-month-old) WT mice, and the decline in endurance exercise capacity caused by Dnmt3a expression worsened with age (Figures 4B and 4C). The older (26-month-old), but not the young (3-month-old), Dnmt3a-Tg mice had dramatically reduced body weight (Figure 4D). Dnmt3a-Tg mice had reduced muscle mass in the gastrocnemius and TA muscles compared to WT mice from a younger age (3-month-old), and the degree of muscle atrophy worsened with age (Figures 4E and 4F). To gain insight into the causal mechanism of the worsened phenotype in older Dnmt3a-Tg mice, we performed microarray analysis of young (3-month-old) and older (26-month-old) WT and Dnmt3a-Tg muscles to evaluate synergistic effects of aging and DNA methylation by Dnmt3a. Heatmap analysis using k-means clustering, and boxplot showed a number of DEGs in older (26-month-old) Dnmt3a-Tg muscle with an FDR <0.05 compared to older (26-month-old) WT muscle (Figure 4G, Table S4). This analysis separated five group clusters, and enrichment analysis revealed the hallmark of each cluster (Figure 4H). Cluster 1 contains genes that were upregulated in young (3-month-old) Dnmt3a-Tg and older (26-month-old) WT muscles compared to young (3-month-old) WT muscle, and these genes showed a further increase in gene expression in older (26-month-old) Dnmt3a-Tg muscle. Cluster 3 contains genes that were upregulated in young (3-month-old) Dnmt3a-Tg compared to young (3-month-old) WT muscle, and these genes showed a further increase in gene expression in older (26-month-old) Dnmt3a-Tg muscle. These genes represent a set of genes where aging and Dnmt3a overexpression synergistically increase gene expression. The genes in cluster 1 and 3 were enriched with genes associated with the inflammation and abnormal immune response, including “inflammatory response,” “allograft rejection,” “IL2 STAT5 signaling,” and “interferon gamma response” (Figures 4H and 4I). GSEA of a senescence-associated gene set (SenMayo) showed that the senescent signatures were increased in older (26-month-old) Dnmt3a-Tg muscle compared to older (26-month-old) WT muscle (Figure 4J). In addition, GSEA of a cytokine-cytokine receptor interaction, a hallmark of aging skeletal muscle,^45^ showed that inflammatory signature was increased in older (26-month-old) Dnmt3a-Tg muscle compared to older (26-month-old) WT and young (3-month-old) Dnmt3a-Tg muscles (Figure 4K). Network visualization and clustering of GSEA results showed that older (26-month-old) Dnmt3a-Tg muscle exhibited enhanced inflammation and immune response compared to older (26-month-old) WT muscle (Figure 4L). Features such as decreased expression of mitochondrial and rRNA- and tRNA-metabolism-related genes were observed in older (26-month-old) Dnmt3a-Tg muscle compared to older (26-month-old) WT muscle (Figures 4L and 4M). The activity of inflammation-associated STAT3 was increased in older (26-month-old) Dnmt3a-Tg muscle compared to older (26-month-old) WT and young (3-month-old) Dnmt3a-Tg muscles, as evidenced by increased phosphorylation of STAT3 relative to total STAT3 in older (26-month-old) Dnmt3a-Tg muscle (Figure 4N). RT-qPCR analysis confirmed that the expression of chemokine (*Ccl8*, *Ccl12*, and *Cxcl10*) and senescence marker (*Cdkn2a/p16*^*Ink4a*^) genes were upregulated and mitochondrial complex I gene (*Ndufa9*) was downregulated in older (26-month-old) Dnmt3a-Tg muscle compared to older (26-month-old) WT and young (3-month-old) Dnmt3a-Tg muscles (Figures 4O and 4P). Reduced mitochondrial complex I level was supported by decreased protein abundance of OXPHOS complex I in old (26-month-old) Dnmt3a-Tg muscle compared with age-matched WT muscle (Figures 4Q and S7F). In addition, the expression of polyamine metabolic enzyme (*Amd1/2*) was downregulated in older (26-month-old) Dnmt3a-Tg muscle compared to older (26-month-old) WT muscle (Figure S7G). Taken together, these results suggest that an increase in Dnmt3a-mediated DNA methylation, in combination with aging, enhances the inflammatory signatures in skeletal muscle, resulting in exacerbation of age-related muscle atrophy, leading to impaired endurance exercise capacity with age.

### Increased DNA methylation is a feature of aging mouse skeletal muscle, and Dnmt3a expression induces hypermethylation in regions where DNA methylation increases with age

**Figure 5:**
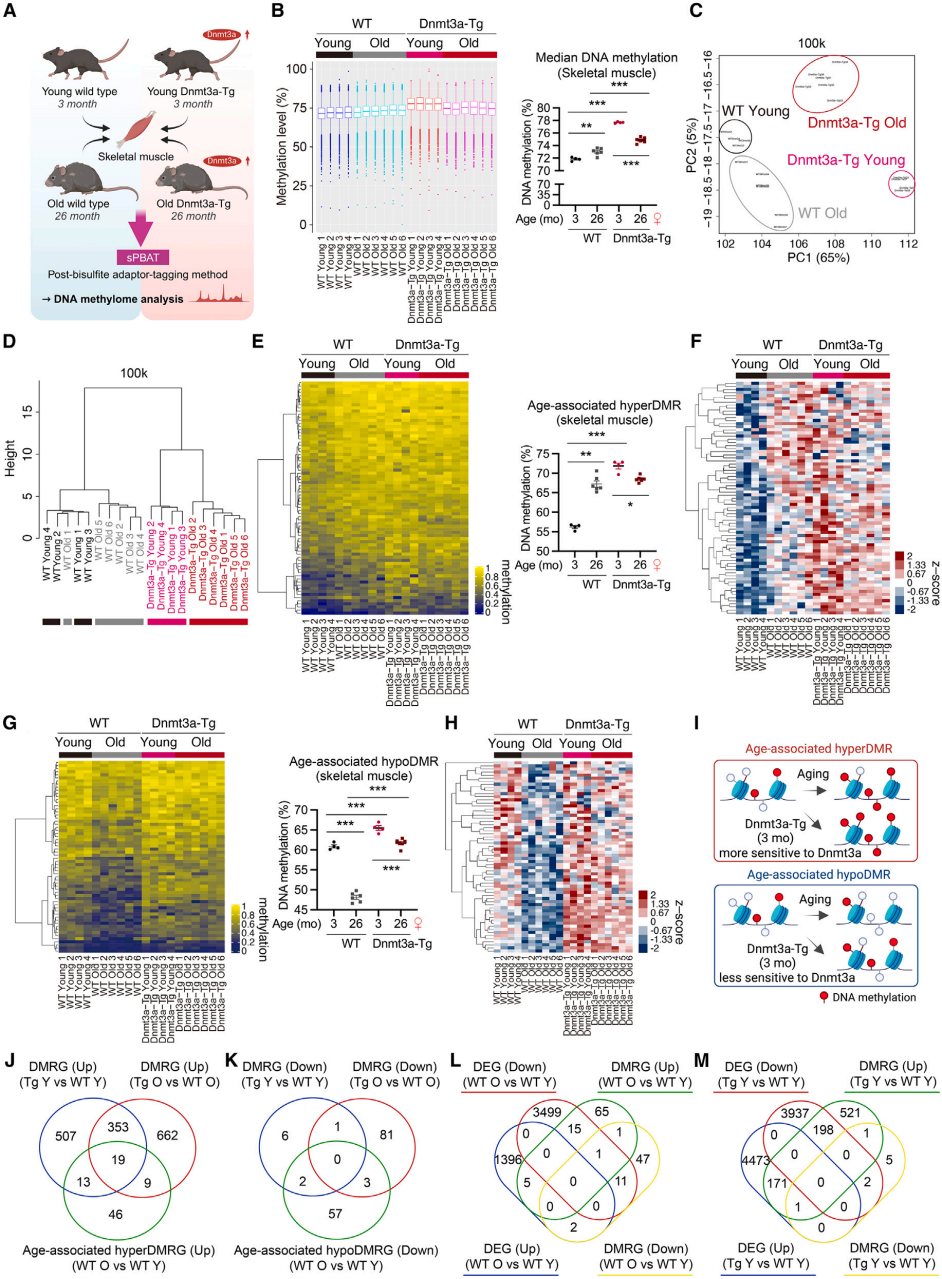
Increased DNA methylation by Dnmt3a induces hypermethylation in regions where DNA methylation increases with age (A) Schematic of the DNA methylome analysis of young and old WT and Dnmt3a-Tg muscles using post-bisulfite adaptor-tagging (sPBAT)-mediated methylome sequencing. (B) Boxplot of mean CpG methylation level of 100-kb sidling windows (3-month-old WT, *n* = 4; 26-month-old WT, *n* = 6; 3-month-old Dnmt3a-Tg, *n* = 4; 26-month-old Dnmt3a-Tg, *n* = 6) (left). The median DNA methylation levels are shown (right). (C) Principal-component analysis of DNA methylation of 100-kb sidling windows (3-month-old WT, *n* = 4; 26-month-old WT, *n* = 6; 3-month-old Dnmt3a-Tg, *n* = 4; 26-month-old Dnmt3a-Tg, *n* = 6). (D) Unsupervised hierarchical clustering using Euclidean distance across the sample set. (E) Heatmap showing DNA methylation levels in the age-associated hypermethylated regions (age-associated hyperDMR: hypermethylated regions in 26-month-old WT muscle compared to 3-month-old WT muscle) in gastrocnemius muscle from WT and Dnmt3a-Tg mice (3-month-old WT, *n* = 4; 26-month-old WT, *n* = 6; 3-month-old Dnmt3a-Tg, *n* = 4; 26-month-old Dnmt3a-Tg, *n* = 6) (left). The average DNA methylation levels in the age-associated hypermethylated regions are shown (right). (F) Heatmap of DNA methylation levels converted to *Z* score in Figure 5E. (G) Heatmap showing DNA methylation levels in the age-associated hypomethylated regions (age-associated hypoDMR: hypomethylated regions in 26-month-old WT muscle compared to 3-month-old WT muscle) in gastrocnemius muscle from WT and Dnmt3a-Tg mice (3-month-old WT, *n* = 4; 26-month-old WT, *n* = 6; 3-month-old Dnmt3a-Tg, *n* = 4; 26-month-old Dnmt3a-Tg, *n* = 6) (left). The average DNA methylation levels in the age-associated hypomethylated regions are shown (right). (H) Heatmap of DNA methylation levels converted to *Z* score in Figure 5G. (I) Schematic of the age- and Dnmt3a-mediated DNA methylation in the gastrocnemius muscle showing that age-associated hyperDMR is more sensitive to Dnmt3a, and age-associated hypoDMR is less sensitive to Dnmt3a, at least at young ages. (J and K) Venn diagram showing the overlap of DMRGs by aging and Dnmt3a expression. (L and M) Venn diagram showing that DMRGs by aging and Dnmt3a expression partially matched the differentially expressed genes (DEGs). All data indicate mean ± SE. ∗*p* < 0.05, ∗∗*p* < 0.01, ∗∗∗*p* < 0.001. (B, E, and G) Two-way analysis of variance (ANOVA) followed by Tukey’s *post hoc* test. WT, wild type; Tg, Dnmt3a-Tg; Y, Young, O: Old.

To compare changes in the DNA methylome due to aging and Dnmt3a overexpression, we performed PBAT-mediated methylome analysis using gastrocnemius muscles from young (3-month-old) and older (26-month-old) WT and Dnmt3a-Tg mice (Figure 5A). Analysis of methylation status at CpG sites, principal-component analysis, and unsupervised hierarchical clustering clearly separated the methylation patterns between young WT, older WT, young Dnmt3a-Tg, and older Dnmt3a-Tg muscles (Figures 5B–5D and S8A–S8C). Similar changes of DNA methylation were observed by comparing DNA methylation changes not only in the 100 kb window but also in the 5 kb window (Figures S8A–S8C). Previous studies have shown that DNA methylation is increased in aging human skeletal muscle,^16^^,^^17^ and promoter DNA methylation is increased in aging rodent skeletal muscle.^18^ The results of PBAT-mediated methylome sequencing showed that DNA methylation is highly stable and DNA methylation was significantly increased in aging WT mouse skeletal muscle (Figures 5B and 5C). DNA methylation levels in older Dnmt3a-Tg muscle were relatively lower than in younger Dnmt3a-Tg muscle, although this may be a technical issue, as described in “Limitation of the study,” the details of which are unknown (Figures 5B and S8D). To understand the effect of aging on the DNA methylome, we identified age-associated differentially methylated regions (DMRs) by comparing the DNA methylome of young and older WT muscles (age-associated hyperDMR: Figures 5E and 5F; age-associated hypoDMR: Figures 5G and 5H; see also Table S5). At young ages, Dnmt3a overexpression markedly increased hypermethylation in the regions where DNA methylation increased with age in WT muscle (Figures 5E and 5F), whereas it slightly increased hypermethylation in regions where DNA methylation decreased with age (Figures 5G and 5H). These results suggest that age-associated hyperDMR are more sensitive to Dnmt3a and age-associated hypoDMR are less sensitive to Dnmt3a, at least at young ages (Figure 5I). The DMRs or DMR-associated genes (DMRGs) altered by Dnmt3a expression were, as expected, more numerous than the age-associated DMRs or DMRGs, suggesting that the effects of DNA hypermethylation by Dnmt3a overexpression may be greater than the effects of DNA hypermethylation by aging (Figures 5J, 5K, S8E, and S8F; see also Table S5). The DMRGs by aging and Dnmt3a expression were partially consistent with the change in expression of DEGs (Figures 5L, 5M, and S8G; Table S6). To evaluate the features of DMRG by aging and Dnmt3a expression, we performed GO enrichment analysis and KEGG pathway analysis. A recent study of human skeletal muscle aging showed that pathways involved in muscle structural integrity and innervation, such as the focal adhesion and axon guidance pathways, are lost with age.^48^ Aging- and Dnmt3a-associated hyperDMRG were associated with the muscle structural integrity and innervation, including “cell adhesion,” “neuron migration,” and “nervous system development” (Figures S9A–S9H and S10A). In addition, examination of the DNA methylation status of gene bodies or in the vicinity of genes of interest among those with altered expression in Dnmt3a-Tg muscle, particularly those related to muscle fiber type, identified possible Dnmt3a-targeted regions where DNA methylation in or near the gene bodies was increased by Dnmt3a expression, independent of aging (Figures S10B, S10C, and S11A–S11C). Taken together, these data suggest that increased DNA methylation by Dnmt3a partially induces DNA hypermethylation in the age-associated hyperDMR. Although Dnmt3a overexpression increases DNA methylation more broadly than the accumulation of DNA methylation with age, Dnmt3a-Tg mice may be useful for studying the effects of the aging characteristics of increased DNA methylation on skeletal muscle homeostasis.

### Dnmt3a not only causes loss of sensitivity to starvation but also reduces metabolic elasticity

**Figure 6:**
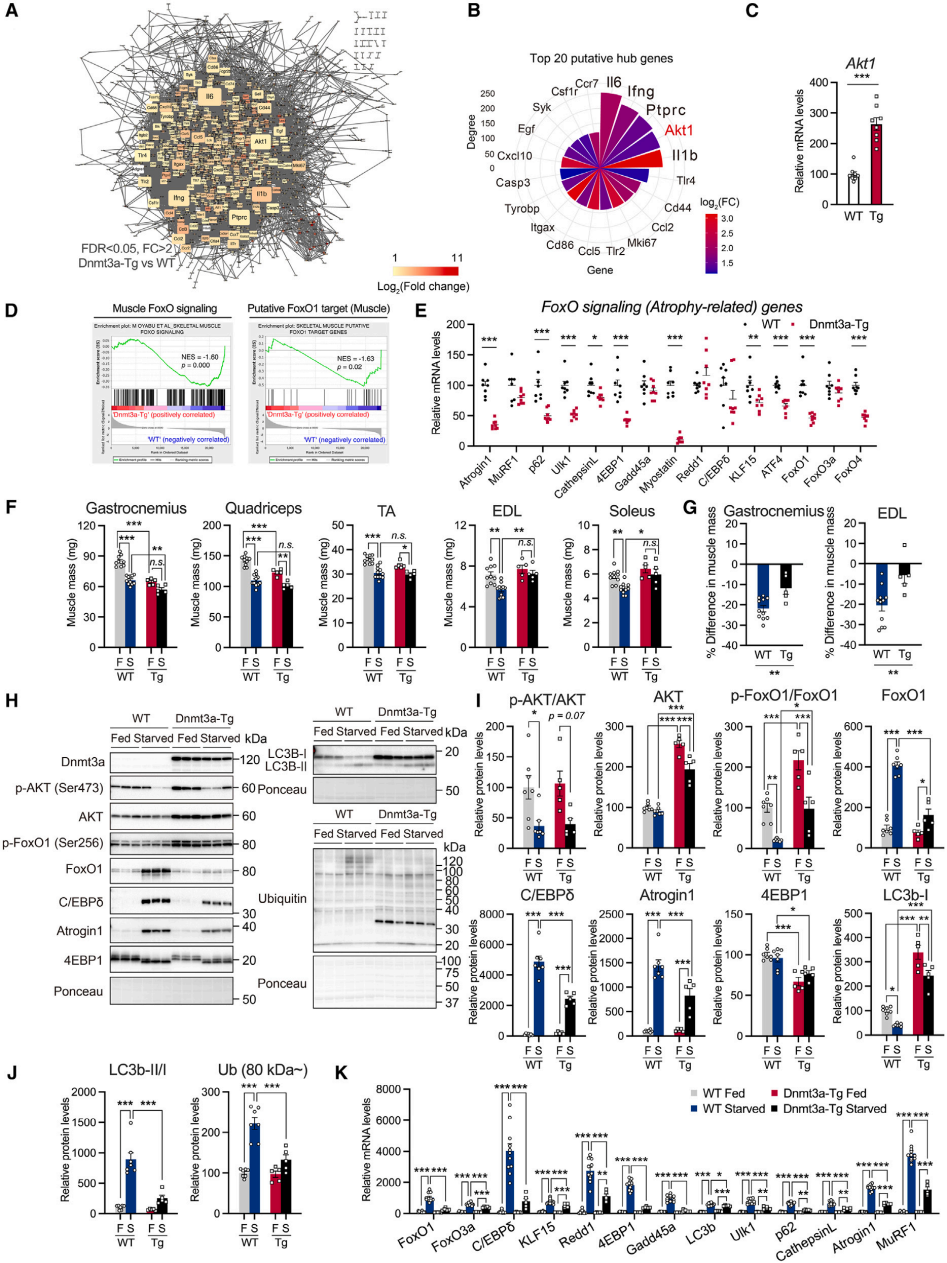
Dnmt3a regulates the Akt-FoxO-atrogene axis and causes loss of sensitivity to starvation (A) PPI network of differentially expressed genes (DEGs) with FDR <0.05 and fold-change value >2 upregulated in Dnmt3a-Tg muscle compared with WT muscle (1,534 nodes and 12,704 edges are shown). Node color indicates log_2_ fold-change value, and circle size of nodes indicates number of direct edges (degree). (B) The top 20 putative hub genes among the network of DEGs upregulated in Dnmt3a-Tg muscle were identified by PPI network analysis. (C) Relative mRNA expression of *Akt1* in gastrocnemius muscle from young (3-month-old) WT and age-matched Dnmt3a-Tg mice (*n* = 8 mice/group). (D) GSEA of a gene set of muscle FoxO signaling and putative FoxO1 target in skeletal muscle from microarray data of young (3-month-old) WT and Dnmt3a-Tg muscles (*n* = 4 mice/group). (E) Relative mRNA expression of FoxO signaling pathway and atrophy-related genes in gastrocnemius muscle from young (3-month-old) WT and Dnmt3a-Tg mice (*n* = 8 mice/group). (F) Weights of skeletal muscles (gastrocnemius, quadriceps, TA, EDL, and soleus) from fed and 48-h-fasted WT and Dnmt3a-Tg female mice (*n* = 10–11 fed and 48-h-fasted WT mice, *n* = 5 fed and 48-h-fasted Dnmt3a-Tg mice). (G) Starvation-induced difference in skeletal muscle mass of gastrocnemius and EDL muscles in WT and Dnmt3a-Tg female mice (*n* = 11 WT mice, *n* = 5 Dnmt3a-Tg mice). (H–J) Representative immunoblot images (H) and densitometric analysis (I, J) of Dnmt3a, p-Akt (Ser473), AKT, p-FoxO1 (Ser256), FoxO1, C/EBPδ, Atrogin1, 4EBP1, LC3b, and ubiquitin protein in the gastrocnemius of fed and 48-h-fasted WT and Dnmt3a-Tg female mice (*n* = 7 fed and 48-h-fasted WT mice, *n* = 5 fed and 48-h-fasted Dnmt3a-Tg mice). (K) Relative mRNA expression of atrogenes downstream of FoxO in gastrocnemius muscles from WT and Dnmt3a-Tg mice (*n* = 10–11 fed and 48-h-fasted WT mice, *n* = 5 fed and 48-h-fasted Dnmt3a-Tg mice). All data indicate mean ± SE. ∗*p* < 0.05, ∗∗*p* < 0.01, ∗∗∗*p* < 0.001. (C, E, G) Student’s two-tailed unpaired t test. (F, I–K) Two-way analysis of variance (ANOVA) followed by Tukey’s *post hoc* test. F, fed; S, starvation; WT, wild type; Tg: Dnmt3a-Tg.

**Figure 7:**
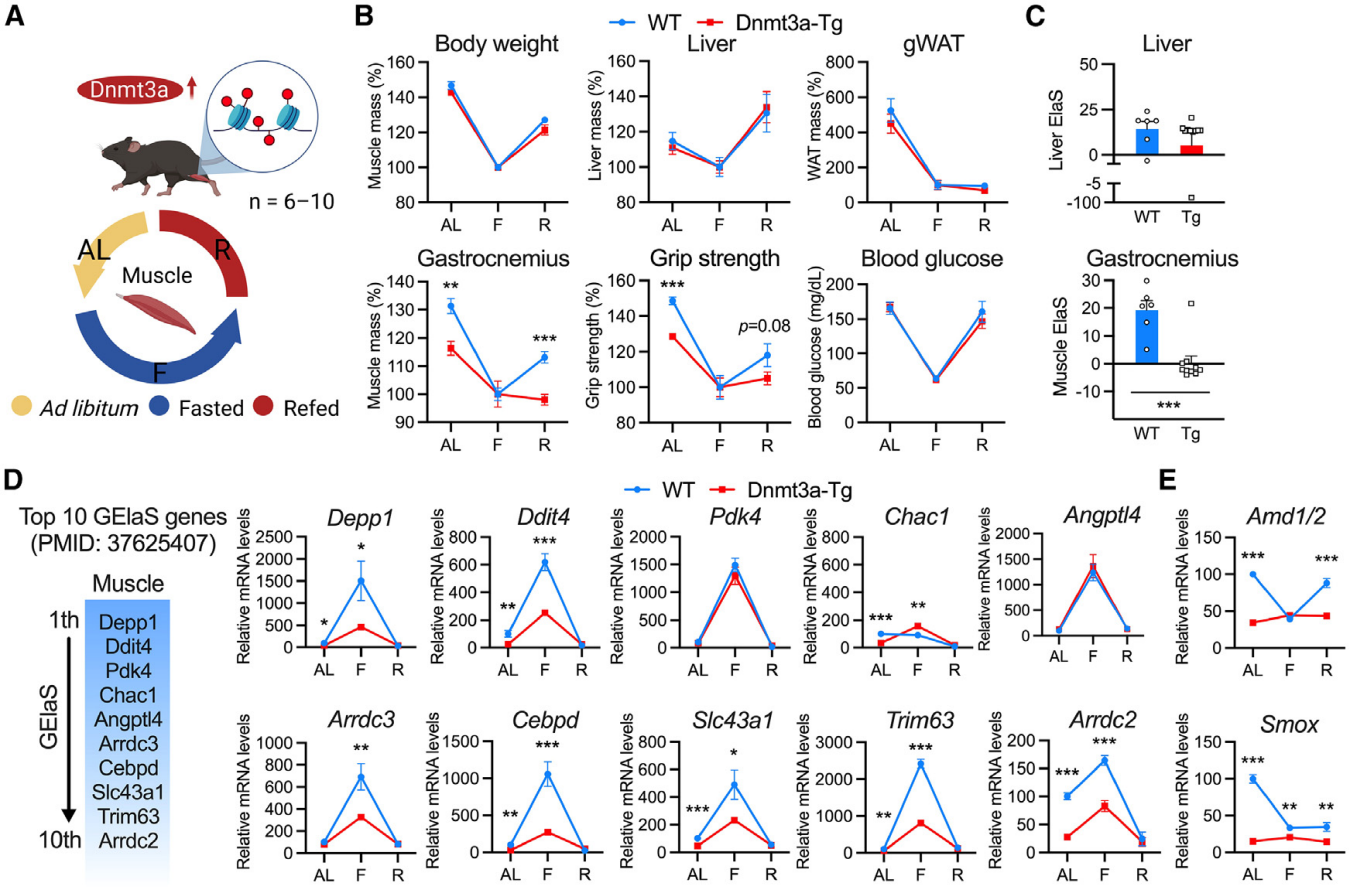
Dnmt3a reduces restorability from starvation-induced muscle atrophy (A) A schematic diagram of the *ad libitum*-fasting-refeeding (AL-F-R) cycle, focusing on muscle mass regulation by sustained Dnmt3a overexpression. (B) The relative values of body weight, liver mass, gonadal white adipose tissue (gWAT) mass, gastrocnemius muscle mass, grip strength, and blood glucose levels in one AL-F-R cycle. The values are normalized to the fasting state of WT and Dnmt3a-Tg mice, respectively. (C) Liver and muscle elasticity scores (ElaS) in WT and Dnmt3a-Tg mice in one AL-F-R cycle. (D) The previously reported top 10 genes with high gene elasticity scores (GElaS) in skeletal muscle^93^ and relative mRNA expression of top 10 genes with high GElaS in gastrocnemius muscles of 3-month-old fed, fasted, and refed WT and Dnmt3a-Tg mice (n = 6–10 mice/group). (E) Relative mRNA expression of polyamine-metabolism-related genes in gastrocnemius muscles of 3-month-old fed, fasted, and refed WT and Dnmt3a-Tg mice (n = 6–10 mice/group). All data indicate mean ± SE. ∗*p* < 0.05, ∗∗*p* < 0.01, ∗∗∗*p* < 0.001. (B, C, D, and E) Student’s two-tailed unpaired t test. AL, *ad libitum*, F, fasting; R, refeeding; WT, wild type; Tg, Dnmt3a-Tg.

To identify putative hub genes within the transcriptome upregulated in response to Dnmt3a overexpression, we used the STRING database^54^ to construct PPI networks from DEGs in Dnmt3a-Tg muscle (Figure 6A). PPI network analysis identified putative hub genes with a large number of direct edges (PPI) (top 20 genes are shown in Figure 6B). Among the hub genes, Akt1 was the 4th gene with the large number of direct edges, indicating that Akt1 functions as a central hub gene under the networks of Dnmt3a-upregulated genes (Figure 6B). RT-qPCR analysis confirmed that Akt1 expression increased in Dnmt3a-Tg muscle (Figure 6C). Studies have shown that transient Dnmt3a overexpression attenuated denervation- and diabetes-induced muscle atrophy.^38^^,^^39^ In diabetes, Dnmt3a modulates the Pten/Akt pathway and prevents muscle atrophy. Akt is one of the most important downstream factors of the insulin/IGF-1 signaling pathway that regulates skeletal muscle atrophy through the FoxO-atrogene axis.^86^^,^^87^^,^^88^^,^^89^^,^^90^ GSEA of the gene sets of muscle FoxO signaling and putative FoxO1 target genes in muscle (Table S7), the gene sets we previously established as FoxO-regulated genes in skeletal muscle,^91^ showed that the skeletal muscle FoxO signaling was decreased in Dnmt3a-Tg muscle compared to WT muscle (Figure 6D). Network analysis showed that a set of atrogenes were downregulated in Dnmt3a-Tg muscle compared with WT muscle (Figure S12A). RT-qPCR analysis confirmed this result (Figure 6E). Because Dnmt3a modulated the Akt-FoxO-atrogene axis, we investigated whether Dnmt3a overexpression could attenuate starvation-induced muscle atrophy. Dnmt3a-Tg mice showed loss of sensitivity to starvation-induced muscle atrophy, as evidenced by reduced starvation-induced muscle mass loss (Figures 6F, 6G, and S12B). Western blot analysis showed that Dnmt3a-Tg muscle had significantly increased Akt protein expression without changing the phosphorylation of Akt (Ser473 and Thr308) relative to total Akt; conversely, starvation-induced upregulation of FoxO1, decreased phosphorylation of FoxO1 relative to total FoxO1, and induction of FoxO1 target proteins (C/EBPδ and Atrogin1/Fbxo32)^91^ were attenuated in Dnmt3a-Tg muscle (Figures 6H, 6I, S12C, and S12D). Dnmt3a-Tg muscle was resistant to activation of starvation-induced autophagy and accumulation of polyubiquitinated proteins, particularly those of high molecular weight (>80 kDa) (Figures 6H and 6J). Dramatic accumulation of LC3b-I, a hallmark of impaired autophagy,^92^ occurred in Dnmt3a-Tg muscle under fed conditions (Figures 6H and 6I), suggesting impaired basal autophagy. RT-qPCR analysis showed that FoxO downstream atrogenes were upregulated by starvation in WT muscle, and the upregulation of these genes was attenuated in Dnmt3a-Tg muscle (Figures 6K and S12E). Taken together, these results suggest that Dnmt3a reduces susceptibility to starvation-induced muscle atrophy by modulating the Akt-FoxO-atrogene axis. Recent studies have shown that aging tissues are characterized by a decrease in metabolic elasticity and the ability to adapt to and recover from the environmental changes and that metabolic elasticity is characterized by coordinated versatility in gene expression, which is known as “gene elasticity.”^93^ As the majority of genes with a high gene elasticity score (GElaS) in skeletal muscle^93^ are FoxO1 target genes,^91^ we speculated on the possibility that Dnmt3a-Tg muscle would have reduced metabolic elasticity. To test this hypothesis, we examined the responsiveness of Dnmt3a-Tg muscle not only to fasting but also to refeeding (Figure 7A). The refed Dnmt3a-Tg mice showed a similar restorability from fasting in body weight, liver mass, white adipose tissue mass, and blood glucose levels (Figures 7B and S12F). Although only certain specific skeletal muscles, the gastrocnemius muscle mass showed significantly reduced restorability from muscle atrophy (Figures 7B, 7C, S12F, and S12G). Muscle strength of Dnmt3a-Tg mice also showed a similar trend of reduced restorability from muscle atrophy (Figure 7B). Therefore, we measured the expression of top 10 genes with high GElaS in skeletal muscle^93^ and found that WT muscle showed dynamic responsibility and restorability from fasting in several genes with high GElaS, whereas Dnmt3a-Tg muscle showed low responsibility (Figure 7D). WT muscle also showed dynamic responsibility and restorability from fasting in polyamine-metabolism-related gene (*Amd1/2*), but Dnmt3a-Tg muscle showed loss of responsibility and restorability from fasting (Figure 7E). These data suggest that Dnmt3a expression reduces not only sensitivity to starvation-induced muscle atrophy but also the restorability from muscle atrophy, which leads to reduced metabolic elasticity in skeletal muscle.

### Sustained Dnmt3a overexpression in adult postnatal myofibers disrupts skeletal muscle homeostasis and results in muscle atrophy

**Figure 8:**
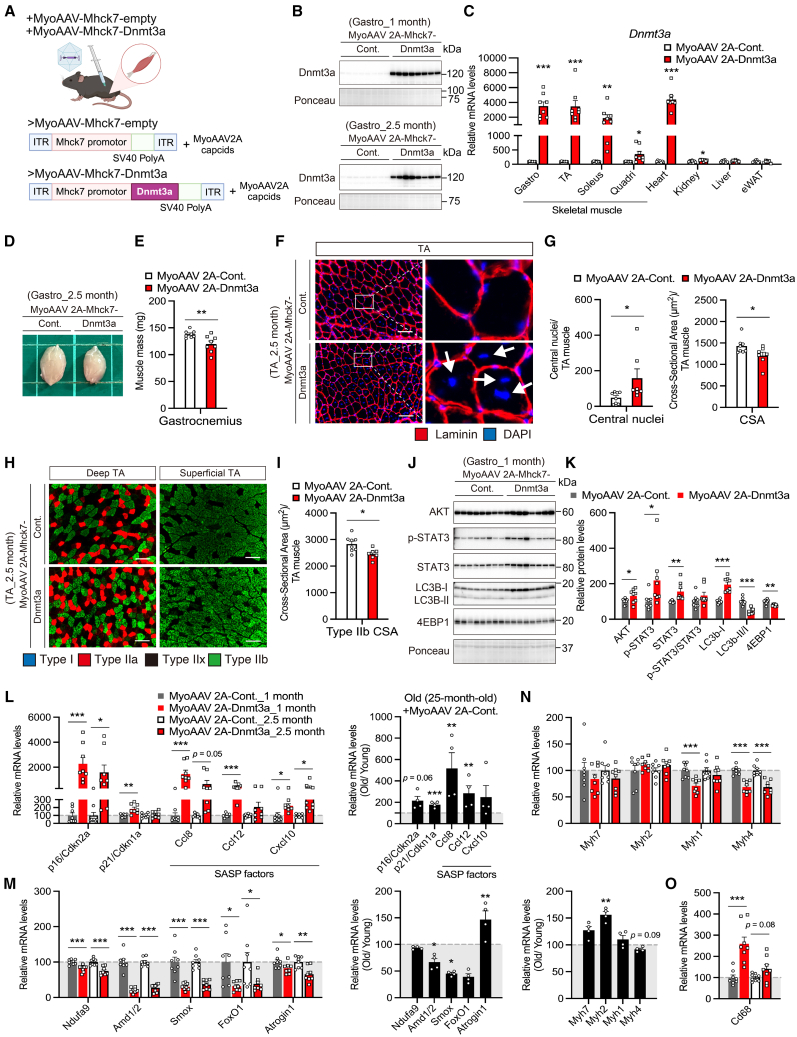
Dnmt3a overexpression in adult postnatal myofibers disrupts skeletal muscle homeostasis and results in muscle wasting (A) A schematic diagram of experiment using MyoAAV-Mhck7-empty and MyoAAV-Mhck7-Dnmt3a vectors. ITR, inverted terminal repeat. (B) Immunoblot of Dnmt3a protein in the gastrocnemius muscles of MyoAAV-Mhck7-empty- or MyoAAV-Mhck7-Dnmt3a-treated mice (*n* = 8 mice/group) (1 or 2.5 months after AAV injection). (C) Relative mRNA expression of Dnmt3a in in the skeletal muscle and other tissues of MyoAAV-Mhck7-empty- or MyoAAV-Mhck7-Dnmt3a-treated mice (*n* = 8 mice/group) (2.5 months after AAV injection). (D) Representative photographs of gastrocnemius muscles (2.5 months after AAV injection). (E) Weights of gastrocnemius muscles from MyoAAV-Mhck7-empty- or MyoAAV-Mhck7-Dnmt3a-treated mice (*n* = 8 mice/group) (2.5 months after AAV injection). (F) Representative images of immunohistochemical staining of laminin and DAPI (laminin, red and DAPI, blue) in TA muscle cross-section from MyoAAV-Mhck7-empty- or MyoAAV-Mhck7-Dnmt3a-treated mice (2.5 months after AAV injection). Scale bar, 100 μm. White arrows indicating observed central nuclei in a muscle section. (G) Quantification of central nuclei (left) and average muscle fiber cross-sectional area (CSA) (right) of TA section (n = 7–8 mice/group) (2.5 months after AAV injection). (H) Representative images of immunohistochemical staining of myosin heavy chains (type I, blue; type IIa, red; type IIb, green) in deep and superficial TA muscle cross-section from MyoAAV-Mhck7-empty- or MyoAAV-Mhck7-Dnmt3a-treated mice (2.5 months after AAV injection). Scale bar, 100 μm. (I) Quantification of type IIb fiber CSA (right) of deep TA section (n = 7–8 mice/group) (2.5 months after AAV injection). (J and K) Immunoblot images (J) and densitometric analysis (K) of AKT, p-STAT3, STAT3, LC3b, and 4EBP1 proteins in the gastrocnemius muscles of MyoAAV-Mhck7-empty- or MyoAAV-Mhck7-Dnmt3a-treated mice (*n* = 8 mice/group) (1 month after AAV injection). (L–O) Relative mRNA expression of genes related to senescence and SASP factors (L), mitochondrial complex I, polyamine metabolism, FoxO1 signaling (M), myosin heavy chain (N), and *Cd68* (O) in the gastrocnemius muscles of MyoAAV-Mhck7-empty- or MyoAAV-Mhck7-Dnmt3a-treated mice (*n* = 8 mice/group) (1 or 2.5 months after AAV injection). The black bar shows the relative mRNA expression in the gastrocnemius muscles of old (25-month-old) mice treated with MyoAAV-Mhck7-empty compared to young (3-month-old) mice treated with MyoAAV-Mhck7-empty (*n* = 4 mice/group). All data indicate mean ± SE. ∗*p* < 0.05, ∗∗*p* < 0.01, ∗∗∗*p* < 0.001. Student’s two-tailed unpaired t test.

To rule out the effects of epigenetic reprogramming in early myogenesis due to Dnmt3a overexpression, which was a concern when using the Dnmt3a-Tg line, we performed a Dnmt3a overexpression experiment in adult postnatal myofibers using adeno-associated virus (AAV) (Figure 8A). Adult myocyte tropism was enhanced using the MyoAAV serotype^94^ and confirmed by RT-qPCR and western blot analysis 1 or 2.5 months after a single intramuscular (i.m.) injection of AAV (Figures 8B, 8C, and S13A). One month of Dnmt3a overexpression by MyoAAV did not reduce skeletal muscle mass, whereas 2.5 months of Dnmt3a overexpression by MyoAAV clearly reduced skeletal muscle mass (Figures 8D, 8E, S13B, and S13C). Immunohistochemical analysis of TA muscle section revealed that Dnmt3a overexpression was sufficient to increase myofiber damage and reduce myofiber size, as evidenced by an increased number of central nuclei and decreased muscle cross-sectional area, respectively (Figures 8F, 8G, and S13D). Dnmt3a overexpression was sufficient to reduce the cross-sectional area of fast-twitch (type IIb) myofibers in TA muscle, which is a phenotypic hallmark of sarcopenia (Figures 8H, 8I, S13E, and S13F). One-month overexpression of Dnmt3a by MyoAAV increased total Akt and STAT3 and decreased 4EBP1 protein levels (Figures 8J, 8K, S13G, and S13H). In addition, MyoAAV-mediated Dnmt3a overexpression led to accumulation of LC3b-I and decreased LC3b-II/LC3b-I ratio, suggesting that Dnmt3a overexpression resulted in impaired autophagy (Figures 8J, 8K, S13G, and S13H). MyoAAV-mediated Dnmt3a overexpression also resulted in upregulation of genes associated with the senescence (*Cdkn2a/p16*^*Ink4a*^ and *Cdkn1a/p21* ^*CIP1*^) and SASP factors (*Ccl8*, *Ccl12*, and *Cxcl10*), partially similar to older mice (Figures 8L and S13I), suggesting that Dnmt3a overexpression in adult postnatal myofibers was sufficient to increase inflammatory signaling. Conversely, MyoAAV-mediated Dnmt3a overexpression resulted in downregulation of genes associated with the mitochondrial complex I (*Ndufa9*), polyamine metabolism (*Amd1/2* and *Smox*), and FoxO1 signaling (*FoxO1* and *Atrogin1*/*Fbxo32*) (Figures 8M and S13J). Consistent with the decreased cross-sectional area of type IIb myofibers, the expression of *Myh4* gene was decreased in skeletal muscle by overexpression of Dnmt3a via MyoAAV (Figures 8N and S13K). The expression of an immune-system-related gene (Cd68) and a mitochondrial-biogenesis-related gene (PGC1α) was also increased and decreased, respectively, by MyoAAV-mediated Dnmt3a overexpression (Figures 8O and S13L). These data further clarify the relationship between Dnmt3a overexpression and the age-related phenotype in adult mice.

### Knockout of Dnmt3a in muscle reduces endurance exercise capacity

Since overexpression of Dnmt3a in skeletal muscle led to increased DNA methylation and impaired skeletal muscle function, we next focused on phenotypic changes in Dnmt3a-deficient mice (Dnmt3a-KO mice). Although two papers reported mice lacking Dnmt3a specifically in muscle using a muscle creatine kinase promoter (Dnmt3a^flox/flox^;mCK-Cre Tg: Dnmt3a-KO mice), there was no consensus on the phenotype in terms of physiological function of Dnmt3a-KO mice.^42^^,^^95^ On the one hand, no change in endurance exercise capacity was reported in Dnmt3a-KO mice.^95^ On the other hand, a decrease in endurance exercise capacity was observed in Dnmt3a-KO mice.^42^ As a causal factor in the latter, the increased expression of the enzyme ALDH1L1 associated with the decreased expression of Dnmt3a in Dnmt3a-KO mice promotes the production of NADPH from NADP^+^, which in turn leads to the accumulation of reactive oxygen species (ROS) produced by NADPH oxidase (NOX), resulting in impaired muscle function. Indeed, it has been reported that impaired endurance exercise capacity in Dnmt3a-KO mice can be alleviated by *in vivo* knockdown of ALDH1L1 or intraperitoneal administration of the antioxidant N-acetylcysteine (NAC).^42^

**Figure 9:**
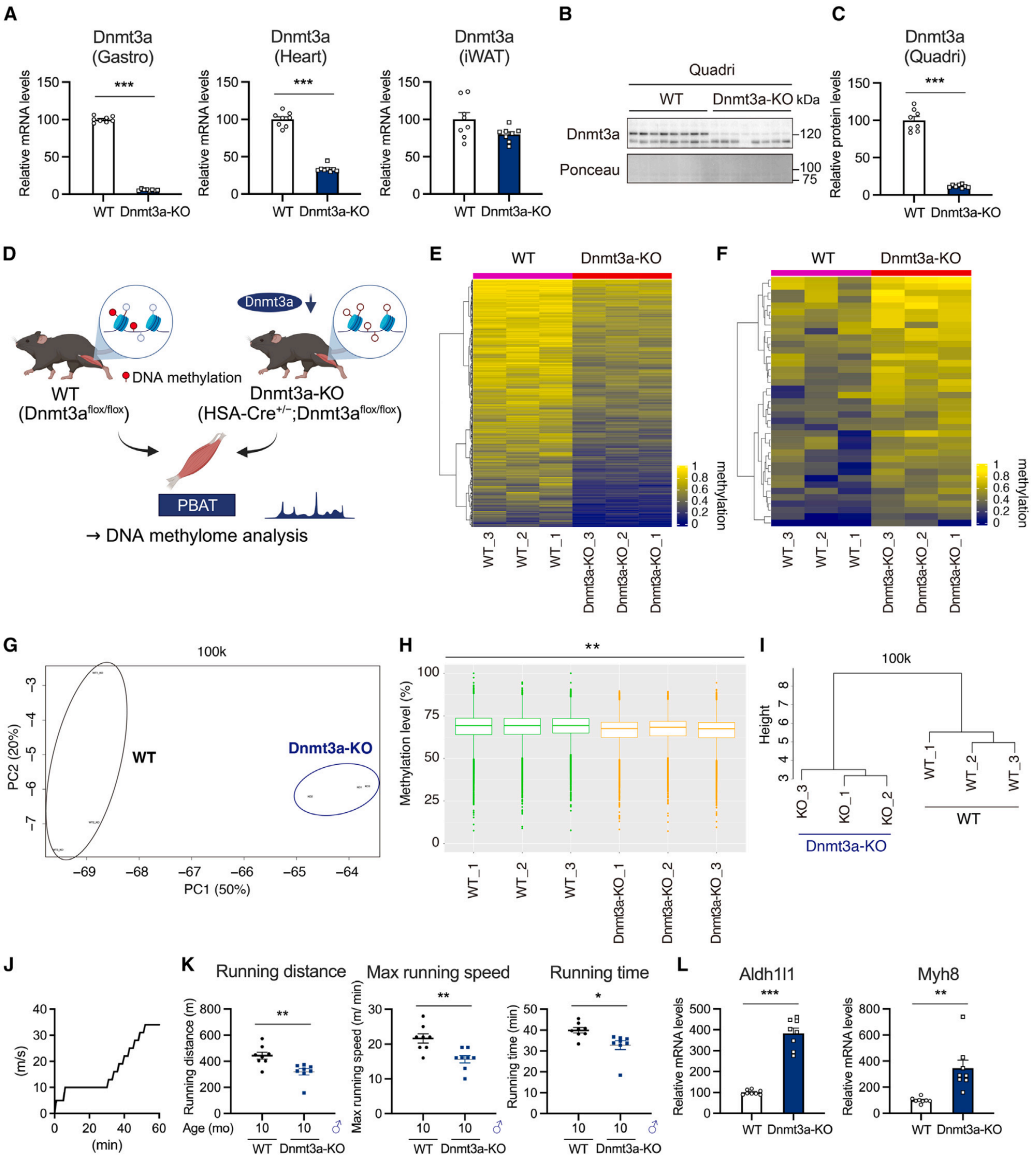
Knockout of Dnmt3a in muscle reduces endurance exercise capacity (A) Relative mRNA expression of Dnmt3a in the gastrocnemius muscle, heart, and inguinal white adipose tissue (iWAT) of WT and Dnmt3a-KO mice (*n* = 8 mice/group). (B and C) Immunoblot images (B) and densitometric analysis (C) of Dnmt3a protein in the quadriceps muscles of WT and Dnmt3a-KO mice (*n* = 8 mice/group). (D) Schematic of the DNA methylome analysis of WT and Dnmt3a-KO muscles using post-bisulfite adaptor-tagging (PBAT)-mediated methylome sequencing. (E and F) Heatmap showing hypomethylated (E) and hypermethylated (F) DMRs in TA muscle from Dnmt3a-KO mice compared with WT mice, identified using PBAT-mediated targeted methylome sequencing (*n* = 3 mice/group). (G) Principal-component analysis of DNA methylation of 100-kb sidling windows (*n* = 3 mice/group). (H) Boxplot of mean CpG methylation level of 100-kb sidling windows (*n* = 3 mice/group). Significant differences in median DNA methylation levels are shown as ∗∗*p* < 0.01. (I) Unsupervised hierarchical clustering using Euclidean distance across the sample set (*n* = 3 mice/group). (J) Protocol of the treadmill running performance test. (K) Total running distance and time, as well as peak speed of WT and Dnmt3a-KO mice in a treadmill running test (*n* = 8 mice/group). (L) Relative mRNA expression of *Aldh1l1* and *Myh8* in the gastrocnemius muscle, heart, and inguinal white adipose tissue (iWAT) of WT and Dnmt3a-KO mice (*n* = 8 mice/group). All data indicate mean ± SE. ∗*p* < 0.05, ∗∗*p* < 0.01, ∗∗∗*p* < 0.001. Student’s two-tailed unpaired t test.

As there was no consensus on the phenotype of Dnmt3a-KO mice as mentioned earlier, we therefore analyzed the exercise capacity of mice lacking Dnmt3a in a muscle-specific manner using a different muscle-specific promoter (human skeletal α-actin promoter) (Dnmt3a^flox/flox^;HSA-Cre Tg: Dnmt3a-KO^41^). These Dnmt3a-KO mice did not show a muscle atrophy phenotype (e.g., reduced skeletal muscle mass or increased expression of inflammation and cellular senescence related genes) (Figures 9A–9C, S14A, and S14B). In contrast, DNA methylation in skeletal muscle was significantly reduced in Dnmt3a-KO mice (Figures 9D–9I and S14C–S14E), and reduced endurance exercise capacity was observed in middle-aged (10-month-old) male Dnmt3a-KO mice (Figures 9J and 9K). Decreased levels of DNA methylation in skeletal muscle of Dnmt3a-KO mice were confirmed by comparing DNA methylation changes not only in the 100 kb window but also in the 5 kb window (Figures S14C–S14E). In addition, the expression of Aldh1l1, which is responsible for ROS production, and Myh8 was increased as shown in a previous study^42^ (Figure 9L). Finally, we identified several candidate genes whose DNA methylation is regulated by Dnmt3a, based on a comparative analysis of DMRGs in skeletal muscle from Dnmt3a-Tg and Dnmt3a-KO mice (Figures S15A–S15C; Table S8). Based on these results, we conclude that reduced DNA methylation due to Dnmt3a deficiency could also lead to impaired skeletal muscle function, i.e., both increased and decreased DNA methylation affect physiological function.

## Discussion

This is the study to demonstrate that increased DNA methylation in skeletal muscle could be a primary driver of skeletal muscle homeostasis disorders and aging-like changes. Loss of transcriptional networks and epigenetic information over time accelerates aging—known as the “Information Theory of Aging.’’^8^ Meta-analysis of altered DNA methylation in human aging skeletal muscle revealed that many of differentially methylated genes were related to skeletal muscle structure and development.^20^ In this study, we observed that hypermethylation of the regions could be associated with the muscle structural integrity and innervation by Dnmt3a overexpression. A vast majority of cell-type-specific genes are often specifically hypomethylated in their cell type,^5^ whereas DNA methylation of tissue-specific gene is increased in older skeletal muscle.^17^^,^^96^ Thus, the increased DNA methylation of the regions is likely to be associated with the muscle structural integrity, and innervation in Dnmt3a-Tg muscle may have led to the disruption of skeletal muscle homeostasis and disturbance of their identity. Indeed, we observed an increased number of central nuclei in myofibers in Dnmt3a-Tg muscle, which is also a hallmark of muscle degeneration with age,^45^^,^^73^^,^^74^ suggesting that DNA methylation balance is important for maintaining muscle homeostasis. The myofiber type shift observed in Dnmt3a-Tg muscle may be due to the disruption of muscle homeostasis and an adaptive response to attenuate stress by increasing the number of stress-resistant type I myofibers. Indeed, central-nucleus-positive type I myofibers were almost absent in Dnmt3a-Tg muscle, whereas central-nucleus-positive myofibers dramatically increased in type II myofibers (Figures 3A–3C). A proportion of type I myofibers was not reduced in skeletal-muscle-specific Dnmt3a-KO mice, suggesting that Dnmt3a is dispensable for muscle fiber type determination. In another study, treatment with histone deacetylase and Dnmt inhibitors could improve loss of muscle strength associated with congenital myopathy in mice with mutations in ryanodine receptor 1.^97^ Based on our findings, we propose that increased DNA methylation may be closely associated with the development of skeletal muscle homeostasis disorders and age-related diseases, such as sarcopenia. Although it would be interesting to know whether Dnmt3a depletion in skeletal muscle would offset the increase in DNA methylation, it is unlikely that the age-related phenotype would be ameliorated in old Dnmt3a-KO mice, since middle-aged male Dnmt3a-KO mice showed reduced exercise capacity compared to age-matched WT mice.

Sarcopenia is characterized by a specific atrophy of the fast-twitch muscle, and the fast-to-slow transition of myofibers is a feature of skeletal muscle aging.^73^^,^^98^ We observed that the weight of fast-twitch muscles was specifically reduced in Dnmt3a-Tg muscle, whereas the weight of slow-twitch muscle increased. A shift in skeletal muscle fiber type from fast to slow occurred in Dnmt3a-Tg muscle. Previous studies have shown that aging human skeletal muscle has an increased proportion of type I myofibers,^48^^,^^49^ which is consistent with the change of Dnmt3a-Tg muscle. In addition, Dnmt3a-Tg muscle showed an increase in local inflammatory markers, denervation markers, and senescent markers; decreased AR signaling; low basal autophagy; reduced mitochondrial OXPHOS complex I protein level; reduced muscle strength; and impaired endurance exercise capacity during aging, which may have partially fulfilled aging-like changes in skeletal muscle.^6^ Recent evidence showed that aging skeletal muscle is characterized by senescent cell burden and an increased inflammatory signature,^45^^,^^46^^,^^47^ and this aging-like inflammatory niche is created by multiple factors, including reactive oxygen species, DNA damage, DNA damage response, loss of silencing at repetitive elements (e.g., LINE1 derepression), and senescent cells.^44^^,^^46^^,^^99^^,^^100^ However, the direct evidence for the effect of epigenetic changes on aging-associated inflammation (“inflammaging”) is scarce. Our study showed that chronic increase in DNA methylation by Dnmt3a not only drives inflammatory and senescent signatures at young age but also robustly promotes the inflammatory signature in skeletal muscle with age. Therefore, the increased DNA methylation in skeletal muscle, in combination with aging, could drive an “inflammaging”-like change and age-related muscle atrophy in a manner that is enhanced with age. One possible explanation of the inflammatory phenotype that worsened with age in Dnmt3a-Tg muscle is a vicious circle between inflammation and senescence, in which SASP factors secreted by senescent cells promote chronic inflammation, which can accelerate senescence in normal cells such as immune cells, resulting in a weakened immune system and an inability to clear senescent cells and inflammatory factors.^101^ As skeletal muscle is a potential central tissue that induces the pathogenesis of sarcopenia and immune senescence,^102^ a more detailed analysis of our skeletal muscle-specific Dnmt3a-Tg mice may be useful to understand the relationship between epigenetic alteration and the pathogenesis of sarcopenia and immune senescence. In this study, we also showed that Dnmt3a overexpression disrupts skeletal muscle homeostasis and leads to muscle atrophy even in adult postnatal myofibers using myotropic AAV (MyoAAV). This could allow us to rule out the effects of epigenetic reprogramming in early myogenesis due to Dnmt3a overexpression and further clarify the relationship between Dnmt3a overexpression and the age-related phenotype in adult mice.

If increased DNA methylation causes skeletal muscle dysfunction, in what ways can we reset or rejuvenate dysfunctional and aged skeletal muscle? In terms of aging, exercise training can reset or partially rejuvenate the aging skeletal muscle methylome. For example, resistance training in aged humans can reset (from hypermethylation in aged to hypomethylation after training) the nuclear^19^ and mitochondrial DNA methylome in skeletal muscle.^35^ Also, aerobic exercise training can restore the DNA methylome in cancer survivor’s muscle,^25^ and a large meta-analysis of the methylome of human skeletal muscle samples indicated that exercise training represents “rejuvenation” of the age-related methylation changes.^36^ In rodent model, late-life PoWeR exercise training in older mice prevents aging-associated promoter hypermethylation and promotes a younger DNA methylation age by 8 weeks (about 8% of total lifespan) as measured by epigenetic clocks.^18^^,^^37^ Although there is little evidence in humans regarding the levels of DNMTs versus ten-eleven translocations (TETs), the enzymes that mediate DNA demethylation, acute exercise has been shown to increase TETs but not DNMTs,^28^ supporting that there is a predominance of hypomethylation after exercise. Taken together, these reports and the results of this study suggest a positive feedback relationship whereby exercise can reset the accumulation of DNA methylation, and reduced DNA methylation could prevent abnormal inflammation in aging skeletal muscle, resulting in the maintenance of high exercise capacity. On the flip side, the accumulation of DNA methylation due to non-exercise with age may lead to an abnormal inflammation in skeletal muscle and further decline of exercise capacity, suggesting that a vicious circle may be formed in which DNA methylation can further accumulate and reduce exercise capacity with age. In terms of Dnmt3a-mediated increases in DNA methylation leading to muscle atrophy, this is supported by the fact that the opposite (hypomethylation) is associated with hypertrophy in humans, and when exposed to repeated hypertrophic stimuli, hypomethylation is even greater and associated with the greatest increases in lean mass.^27^

The previous study showed that DNA methylation in highly methylated regions (repeated sequences such as LINE) decreases with age. On the other hand, DNA methylation in low methylated regions was shown to increase with age, suggesting that aging induces a “regression to the mean” of DNA methylation, with a gradual loss of pre-existing methylation and local patterns in genomic regions that were established early in development.^103^ Our data and previous studies have also shown that DNA methylation in skeletal muscle appears to increase with age. So why might aging increase DNA methylation in skeletal muscle? We have previously reported that endogenous DNMT3a protein expression is reduced in skeletal muscle of aged mice.^41^ Endogenous Dnmt3a mRNA expression is also reduced in skeletal muscle of older mice (Figure S8D of this paper; Min et al.^103^). It has also been reported that DNA demethylase (TET) expression is also reduced in aging skeletal muscle.^103^ Therefore, we have not shown clear evidence that increased Dnmt3a expression is the primary cause of increased DNA methylation in aging skeletal muscle (rather, Dnmt3a expression itself decreases with age). The possible causes of increased DNA methylation in aging skeletal muscle includes (1) changes in the accessibility of DNA methyltransferases and demethylases to the genome, (2) reduced ability to maintain skeletal muscle DNA methylation patterns established early in development, and (3) changes in Dnmt3a-interacting proteins. Because exercise reportedly rejuvenates the skeletal muscle DNA methylome, prolonged inactivity, the opposite of exercise, may increase skeletal muscle DNA methylation with age. In addition, DNA methylation by Dnmt3a requires the substrate S-adenosylmethionine (SAM). Previous reports have shown that SAM accumulates in skeletal muscle of older (28-month-old) mice,^104^ so accumulation of SAM in skeletal muscle, or increased activity of DNMTs or decreased activity of TETs, may also increase DNA methylation in skeletal muscle. Alternatively, as skeletal muscle is composed of terminally differentiated, multinucleated myofibers, DNA methylation may accumulate over time due to the reduced supply of new nuclei, which may be associated with the age-related exhaustion of satellite cell pool.^105^ However, further research is needed to test these speculations. It has been suggested that although a consensus has emerged that 5′ promoter methylation represses transcription, some exceptions to this rule have highlighted the need for further studies to assess and interpret the context-specific role of DNA methylation.^106^ A few examples have also been reported where tissue-specific genes were active even when methylation was high across the gene.^106^ These reports would help to understand why Dnmt3a overexpression and the resulting genomic hypermethylation did not lead to a genome-wide downregulation of genes in Dnmt3a-Tg muscle.

Another intriguing finding is that overexpression of Dnmt3a in skeletal muscle not only suppressed sensitivity to starvation but also reduced the restorability from starvation-induced muscle atrophy in certain specific muscles. Under catabolic conditions, FoxO transcription factors promote muscle atrophy by inducing the autophagy and ubiquitin-proteasome systems.^86^^,^^87^^,^^88^^,^^89^ We observed that Dnmt3a-Tg muscle had decreased FoxO signaling and was protected against starvation-induced muscle atrophy, as reported in denervation- and diabetes-induced muscle atrophy.^38^^,^^39^ In this study, we identified Akt1 as a potential hub gene among the transcriptome networks of Dnmt3a-modulated genes and found that Dnmt3a is a strong regulator of the Akt-FoxO signaling pathway. Because Dnmt3a can attenuate FoxO-mediated muscle atrophy, transient Dnmt3a overexpression may act as an epigenetic modulator that maintains muscle mass under the catabolic condition. However, the gastrocnemius muscle with Dnmt3a overexpression showed the reduced restorability from starvation-induced muscle atrophy, suggesting that Dnmt3a overexpression in skeletal muscle may have led to reduced metabolic elasticity.^93^ Furthermore, unlike acute Dnmt3a expression,^38^^,^^39^ sustained Dnmt3a overexpression disrupted muscle homeostasis and led to accelerated muscle atrophy. This suggests that the acute and chronic Dnmt3a overexpression effects on skeletal muscle are distinctly different and that reduced FoxO signaling under conditions of impaired muscle homeostasis in Dnmt3a-Tg muscle not only fails to cause muscle hypertrophy but also reduces muscle plasticity and may accelerate skeletal muscle aging. Indeed, FoxO3a has been reported to have a geroprotective role in skeletal muscle aging.^73^ A future challenge is to investigate whether increased DNA methylation reduces the efficiency of muscle hypertrophy in models of mechanical overload (e.g., tenotomy-induced mechanical overload) and resistance training (e.g., ladder climbing), because reduced muscle plasticity should be characterized by reduced muscle hypertrophy via mechanical stress. Given that Dnmt3a overexpression increases the inflammatory signature in skeletal muscle and that resistance exercise in humans^26^^,^^27^^,^^28^ and mechanical overload in rodents^29^ can hypomethylate the skeletal muscle methylome, Dnmt3a overexpression, which increases DNA methylation, would likely dampen the muscle hypertrophic response. The loss of muscle mass and upregulation of chemokine-related genes in Dnmt3a-Tg muscle were partially attenuated by resistance exercise (ladder climbing), suggesting that resistance exercise can at least attenuate DNA-hypermethylation-induced inflammatory signaling.

In conclusion, this is the study showing that increased DNA methylation in skeletal muscle disrupts muscle homeostasis and leads to (1) increased inflammatory and senescent signatures in skeletal muscle, (2) shift to slow myofibers (occurs in sarcopenia), (3) decreased AR signaling, (4) decreased mitochondrial OXPHOS complex I protein level, (5) low basal autophagy, and (6) fast myofiber-specific muscle atrophy. In addition, this study also demonstrated the potential synergistic effect of increased DNA methylation by Dnmt3a and aging, particularly on skeletal muscle inflammation. DNA methylation in skeletal muscle increases with age. Our study demonstrated the link between DNA methylation, muscle homeostasis, and skeletal muscle aging. Therefore, we propose that interventions targeting DNA methylation, such as exercise, which is known to rejuvenate the DNA methylome,^18^^,^^19^^,^^25^^,^^35^^,^^36^^,^^37^ may have therapeutic potential to rejuvenate skeletal muscle by attenuating muscle senescent and inflammatory signatures and preventing dysfunction caused by aging and other conditions.

### Limitations of the study

Because we used a single transgenic line for Dnmt3a overexpression in this study, we cannot completely rule out the possibility that the reduction in DNA methylation observed in older Dnmt3a-Tg mice (Figure 5B and elsewhere) could be influenced by the human skeletal muscle α-actin (HSA) promoter used to drive Dnmt3a expression or by positional effects related to the site of transgene insertion. In addition, we did not determine how increased DNA methylation affects skeletal muscle homeostasis in Dnmt3a-Tg mice at epigenetic levels other than DNA methylation. A notable finding of our study is that the change in methylation levels near DMGs or DMRGs induced by Dnmt3a expression only partially matched the change in expression of DEGs in postnatal skeletal muscle, which is consistent with the previous meta-analysis reports that many of the DNA methylated genes in aging skeletal muscle do not match changes in mRNA levels.^20^ One possibility to explain this is that the erosion of the epigenetic landscape by increased DNA methylation indirectly affects histone occupancy, histone modification, and chromatin structure, resulting in dramatic changes in gene expression profiles and accelerated aging phenotype, as reported in the double-stranded-DNA-breaks-induced “ICE” (inducible changes to the epigenome) mouse model.^8^ Thus, epigenomic analyses of Dnmt3a-Tg mice are warranted to understand how increased DNA methylation disrupts skeletal muscle homeostasis. We also recognize the limitation that it is difficult to mimic the same DNA methylation pattern as with aging, although we used the Dnmt3a-Tg model to induce the accumulation of DNA methylation at a young age, which “cannot occur naturally in young mice.” A more detailed analysis of site-specific differences in DNA methylation changes and local DNA methylation changes at the high-resolution levels was not performed in this study; however, due to its potential importance, this is an issue to be addressed in a future study. Furthermore, reduced metabolic elasticity by Dnmt3a overexpression is specific to muscle type, but the underlying cause of this specificity is unclear and is one of the challenges to be addressed.

## Resource availability

### Lead contact

Further information and requests for resources and reagents should be directed to and will be fulfilled by the lead contact, Yasutomi Kamei (kamei@kpu.ac.jp).

### Materials availability

Mice used and plasmid constructs will be shared upon reasonable request to the lead contact.

### Data and code availability

• The raw microarray data of mouse skeletal muscle are deposited in the GEO database (GSE252407 and GSE244336) and are publicly available as of the date of publication. The raw whole-genome bisulfite sequencing data of mouse skeletal muscle are deposited in the GEO database (GSE244432 and GSE262342) and are publicly available as of the date of publication.
• This paper does not report original code.
• Any additional information required to reanalyze the data reported in this paper is available from the lead contact upon request.

## Acknowledgments

We thank all laboratory members, Hana Kawai, Erika Ross-Smith, and Ayaka Nuruki (Kyoto Prefectural University) for experimental support. We thank Dr. Xunmei Yuan (Division of Molecular Biotherapy, Cancer Chemotherapy Center, Tokyo, Japan) for the technical advice on the MIAMI analysis. Microarray data analysis was, in part, performed at the Medical Research Support Center, Graduate School of Medicine, Kyoto University. This study was supported by Grants-in-Aids for Scientific Research KAKENHI (22H03539, 21K19728, 23K18436, and 22KJ2596) from the Japanese Ministry of Education, Culture, Sports, Science, and Technology (MEXT, Tokyo). This study was also partially supported by Platform Project for Supporting Drug Discovery and Life Science Research (Basis for Supporting Innovative Drug Discovery and Life Science Research [BINDS]) from 10.13039/100009619AMED under Grant Number JP21am0101103 and by Research Support Project for Life Science and Drug Discovery (BINDS) from 10.13039/100009619AMED under Grant Number 23ama121022j0002. This study was also partially supported by the program of the Joint Usage/Research Center for Developmental Medicine, Institute of Molecular Embryology and Genetics, Kumamoto University. This study was also supported by the Nakatomi Foundation, the Naito Foundation, and the Uehara Memorial Foundation. The funders had no role in study design, data collection and analysis, decision to publish, and preparation of the manuscript.

## Author contributions

M.Oyabu and Y.K. conceived and designed the experiments; M.Oyabu, Y.Ohira, M.F., K.Y., R.K., A.K., H.Y., H.P.O.Q., F.M., H.A., M.Okano, I.H., Y.Ogawa, T.I., K.I., and Y.Ono performed the experiments; M.Oyabu, Y.Ohira, M.F., K.Y., R.K., A.K., F.M., H.A., M.Okano, I.H., Y.Ogawa, T.I., K.I., and Y.Ono analyzed the data; M.Oyabu, K.Y., A.K., Y.H., N.H., F.M., H.A., M.Okano, I.H., H.G., T.Y., S.F., Y.Ogawa, T.I., K.I., Y.Ono, and Y.K. contributed reagents and materials/analysis tools; and M.Oyabu and Y.K. wrote the article. All authors read and approved the final manuscript.

## Declaration of interests

The authors declare that they have no conflicts of interest with the contents of this article.

## STAR★Methods

### Key resources table

**Table:** 

REAGENT or RESOURCE	SOURCE	IDENTIFIER
**Antibodies**		

Rabbit anti-Dnmt3a	Santa Cruz	Cat# sc-20703; RRID: AB_2093990
Rabbit anti-phospho-STAT3 (Tyr705)	Cell Signaling	Cat# 9145; RRID: AB_2491009
Mouse anti-STAT3	Cell Signaling	Cat# 9139; RRID: AB_331757
Rabbit anti-phospho-Akt (Ser473)	Cell Signaling	Cat# 9271; RRID: AB_329825
Rabbit anti-phospho-Akt (Thr308)	Cell Signaling	Cat# 4056; RRID: AB_331163
Rabbit anti-Akt (pan)	Cell Signaling	Cat# 4685; RRID: AB_2225340
Rabbit anti-phospho-FoxO1 (Ser256)	Cell Signaling	Cat# 9461; RRID: AB_329831
Rabbit anti-FoxO1	Cell Signaling	Cat# 2880; RRID: AB_2106495
Rabbit anti-C/EBPδ	Cell Signaling	Cat# 2318; RRID: AB_2078194
Mouse anti-Atrogin1/MAFbx	Santa Cruz	Cat# sc-166806; RRID: AB_2246982
Rabbit anti-4EBP1	Cell Signaling	Cat# 9644; RRID: AB_2097841
Rabbit anti-LC3b	Sigma Aldrich	Cat# L7543; RRID: AB_796155
Rabbit anti-Ubiquitin	Cell Signaling	Cat# 3933; RRID: AB_2180538
Total OXPHOS rodent WB antibody cocktail	Abcam	Cat# ab110413; RRID: AB_2629281
Mouse anti MyHC	DSHB	Cat# MF-20; RRID: AB_2147781
Mouse anti MyHC type I	DSHB	Cat# BA-D5; RRID: AB_2235587
Mouse anti-MyHC type IIa	DSHB	Cat# SC-71; RRID: AB_2147165
Mouse anti MyHC type IIb	DSHB	Cat# BF-F3; RRID: AB_2266724
Rat anti-Laminin α2	Santa Cruz	Cat# sc-59854; RRID: AB_784266
Goat anti-Mouse IgG (H+L) Cross-Adsorbed Secondary Antibody, Alexa Fluor™ 568	Thermo-Fisher	Cat# A11004; RRID: AB_2534072
Goat anti-Mouse IgG2b Cross-Adsorbed Secondary Antibody, Alexa Fluor™ 350	Thermo-Fisher	Cat# A21140; RRID: AB_2535777
Goat Anti-Mouse IgM mu chain (Alexa Fluor® 488)	Abcam	Cat# ab150121; RRID: AB_2801490
Goat Anti-Mouse IgM (Heavy chain) Cross-Adsorbed Secondary Antibody, Alexa Fluor™ 488	Thermo-Fisher	Cat# A21042; RRID: AB_2535711
Goat anti-Mouse IgG1 Cross-Adsorbed Secondary Antibody, Alexa Fluor™ 594	Thermo-Fisher	Cat# A21125; RRID: AB_2535767
Goat anti-Mouse IgG1 Cross-Adsorbed Secondary Antibody, Alexa Fluor™ 568	Thermo-Fisher	Cat# A21124; RRID: AB_2535766
Goat anti-Mouse IgG2b Cross-Adsorbed Secondary Antibody, Alexa Fluor™ 594	Thermo-Fisher	Cat# A21145; RRID: AB_2535781
Goat anti-Rat IgG (H+L) Cross-Adsorbed Secondary Antibody, Alexa Fluor™ 488	Thermo-Fisher	Cat# A11006; RRID: AB_2534074
Goat anti-Mouse IgG1 Cross-Adsorbed Secondary Antibody, Alexa Fluor™ 488	Thermo-Fisher	Cat# A21121; RRID: AB_2535764
Goat anti-Rat IgG (H+L) Cross-Adsorbed Secondary Antibody, Alexa Fluor™ 546	Thermo-Fisher	Cat# A11081; RRID: AB_2534125
Goat anti-Rat IgG (H+L) Cross-Adsorbed Secondary Antibody, Alexa Fluor™ 568	Thermo-Fisher	Cat# A11077; RRID: AB_2534121
Anti-Rabbit IgG, HRP-linked Antibody	GE Healthcare	Cat# NA934V
Anti-Rabbit IgG, HRP-linked Antibody	Cell Signaling	Cat# 7074; RRID: AB_2099233
Anti-Mouse IgG, HRP-linked Antibody	Cell Signaling	Cat# 7076; RRID: AB_330924
Anti-Mouse IgGκ, HRP-linked antibody	Santa Cruz	Cat# sc-516102; RRID: AB_2687626

**Bacterial and virus strains**		

MyoAAV 2A-Mhck7-empty	This study	N/A
MyoAAV 2A-Mhck7-Dnmt3a	This study	N/A

**Chemicals, peptides, and recombinant proteins**		

10% D-glucose solution	Otsuka Pharmaceutical	Cat# 079118 20mL
Humulin R Injection	Lilly	Cat# 02101 10mL
Glutest Neo Sensor	Sanwa Kagaku	Cat# 086532990
Cardiotoxin from *Naja mossambica mossambica*	Sigma-Aldrich	Cat# C9759
4%-Paraformaldehyde Phosphate Buffer Solution	Nacalai Tesque	Cat# 09154-85
Tween 20	Nacalai Tesque	Cat# 35624-15
Goat serum	Sigma-Aldrich	Cat# 9023
Triton X-100	Nacalai Tesque	Cat# 35501-15
DAPI Fluoromount-G	SouthernBiotech	Cat# 0100-20
Isopentane	Nacalai Tesque	Cat# 26405-65
O.C.T.Compound	Sakura	Cat# 4583
M.O.M. Immunodetection Kit, Basic	Vector	Cat# BMK-2202
Fluoro-KEEPER Antifade Reagent, Non-Hardening Type with DAPI	Nacalai Tesque	Cat# 12745-74
Fluoro-KEEPER Antifade Reagent, Non-Hardening Type	Nacalai Tesque	Cat# 12593-64
Dako Fluorescence Mounting Medium	Agilent	Cat# S3023
RIPA Lysis Buffer cocktail	Merck	Cat# 20-188
RIPA Lysis Buffer cocktail	Nacalai Tesque	Cat# 16488-34
Protein inhibitor cocktail	Nacalai Tesque	Cat# 25955-24
Sodium orthovanadate	Sigma-Aldrich	Cat# S6508
Phenylmethylsulfonyl fluoride	Nacalai Tesque	Cat# 27327-81
Dimethy Sulfoxide	Nacalai Tesque	Cat# 13445-45
Pierce™ BCA Protein Assay Kits	Thermo-Fisher	Cat# 23227
Sample Buffer Solution without 2-ME(2x) for SDS-PAGE	Nacalai Tesque	Cat# 30567-12
Dithiothreitol	Sigma-Aldrich	Cat# D9779
Methanol	Nacalai Tesque	Cat# 21914-03
MagicMark™ XP Western Protein Standard	Thermo Fisher	Cat# LC5602
Precision Plus Protein™ Dual Color Standards	Bio-Rad	Cat# 1610374
Tris	Nacalai Tesque	Cat# 35406-75
Glycine	Nacalai Tesque	Cat# 17109-35
SDS	Nacalai Tesque	Cat# 31607-65
e-PAGEL 10%	Atto	Cat# 2331810
e-PAGEL 15%	Atto	Cat# 2331850
0.1%-tTBS(10x)(pH 7.4)	Nacalai Tesque	Cat# 12750-81
Nitrocellulose membrane (0.45 μm)	Bio-Rad	Cat# 1620167
Immuno-Blot PVDF Membrane (0.2 μm)	Bio-Rad	Cat# 1620174
Ponceau S Solution	Sigma-Aldrich	P7170-1L
Nonfat Dry Milk	Cell Signaling	Cat# 9999
Skim Milk	Nacalai Tesque	Cat# 31149-75
Bovine Serum Albumin	Sigma-Aldrich	Cat# A7906
ECL Prime Western Blotting Detection reagent	GE Healthcare	Cat# RPN2232
TRIzol Reagent	Thermo Fisher	Cat# 15596018
Sepasol-RNA Ⅰ Super G	Nacalai Tesque	Cat# 09379-55
Chloroform	Nacalai Tesque	Cat# 08402-55
Isopropanol	Nacalai Tesque	Cat# 29113-95
Ethanol	Nacalai Tesque	Cat# 14713-95
Distilled Water, Deionized, Sterile	Nippongene	Cat# 312-90103
RNeasy Mini kit	Qiagen	Cat# 74104
SurePrint G3 Unrestricted Gene Expression 8x60K Microarray	Agilent	Cat# G4858A
Low Input Quick Amp Labeling Kit, One-Color	Agilent	Cat# 5190-2305
Gene Expression Hybridization Kit	Agilent	Cat# 5188-5242
Agilent One Color RNA Spike Mix Kit	Agilent	Cat# 5188-5282
Gene Expression Wash Buffer 1	Agilent	Cat# 5188-5325
Gene Expression Wash Buffer 2	Agilent	Cat# 5188-5326
Hybridization Gasket Slide Kit	Agilent	Cat# G2534-60014
ReverTra Ace qPCR RT MasterMix with gDNA Remover	Toyobo	Cat# FSQ-301
Thunderbird Next SYBR qPCR Mix	Toyobo	Cat# QPX-201
HpaII	New England Biolabs Japan	Cat# R0171M
MspI	New England Biolabs Japan	Cat# R0106S
Ecoli ligation kit	Takara	Cat# 2160A
Gene Taq	Nippongene	Cat# 318-02871
MinElute PCR Purification Kit	Qiagen	Cat# 28004
Cy3-dCTP	Sigma-Aldrich	Cat# PA53021
Cy5-dCTP	Sigma-Aldrich	Cat# PA55021
BioPrime™ DNA Labeling System	Thermo Fisher	Cat# 18094-011
Oligo aCGH/ChIP-on-chip Hybridization kit	Agilent	Cat# 5188-5220
Custom ChIP-on-chip/DNA Methylation, 4x44K; One glass slide formatted with four high-definition 44K arrays	Agilent	Cat# G4497A
Qubit dsDNA BR Assay Kit	Thermo Fisher	Cat# Q32850
Qubit dsDNA HS Assay Kit	Thermo Fisher	Cat# Q32851
Qubit ssDNA Assay Kit	Thermo Fisher	Cat# Q10212
Agencourt AMPure XP	Beckman Coulter	Cat# A63880
Agilent RNA 6000 Pico Kit	Agilent	Cat# 5067-1513
Klenow Fragment (3'→5' exo-)	New England Biolabs Japan	Cat# M0212M
Bst DNA polymerase large fragment	New England Biolabs Japan	Cat# M0275S
Exonuclease I	New England Biolabs Japan	Cat# M0293S
Phusion Hot Start High-Fidelity DNA Polymerase	Finnzymes	Cat# F-540S
EZ DNA Methylation-Gold Kit	ZYMO Research	Cat# D5005
Dynabeads M-280 Streptavidin	Thermo Fisher	Cat# 112-05D
KAPA Library Quantification Kit for Illumina	KAPA	Cat# KK4824
2.5g/l-Trypsin Solution	Nacalai Tesque	Cat# 35555-54
0.2g/l-EDTA Solution	Nacalai Tesque	Cat# 14367-74
Penicillin-Streptomycin Mixed Solution (Stabilized)	Nacalai Tesque	Cat# 09367-34
Lipofectamine 2000	Thermo Fisher	Cat# 11668019
Opti-MEM™ I Reduced Serum Medium	Thermo Fisher	Cat# 31985-070
DMEM (High glucose)	Nacalai Tesque	Cat# 08459-64; Cat# 08458-16
Puromycin Dihydrochloride	Nacalai Tesque	Cat# 19752-64
Polybrene Solution	Nacalai Tesque	Cat# 12996-81
Type I Collagenase	Worthington	Cat# LS004196
BSA lyophilized powder, BioReagent, suitable for cell	Sigma-Aldrich	Cat# A-9418
Matrigel Growth Factor Reduced Basement Membrane Matrix	Corning	Cat# 354230
DMEM, no glucose, no glutamine, no phenol red	Thermo Fisher	A14430-01
Fetal bovine serum	Sigma-Aldrich	Cat# F9423
Chicken Embryo Extract	US biological	Cat# C3999
GlutaMAX™ Supplement	Thermo Fisher	Cat# 35050061
Recombinant Murine FGF-basic	PEPROTECH	Cat# 450-33
Accutase	Nacalai Tesque	Cat# 12679-54
DMEM, high glucose, GlutaMAX™ Supplement, pyruvate	Thermo Fisher	Cat# 10569-010
Horse Serum	Thermo Fisher	Cat# 16050122
Phenol, Saturated with TE Buffer	Nacalai Tesque	Cat# 26829-96
Proteinase K	Wako	Cat# 169-21041
PureLink™ RNase A	Thermo Fisher	Cat# 12091-021
Takara Taq	Takara	Cat# R001A
Takara Ex Taq	Takara	Cat# RR001A
Agarose for ≧1kbp fragment (Fine Powder)	Nacalai Tesque	Cat# 02468-95
100 bp DNA ladder One	Nacalai Tesque	Cat# 07908-75
6x Loading Dye	Toyobo	Cat# RE-DYE
Ethidium Bromide Solution	Nacalai Tesque	Cat# 14631-94
Sodium Chloride	Nacalai Tesque	Cat# 31320-05
Bacto™ Peptone	DIFCO	Cat# 0118-17-0
Extract Yeast Dried	Nacalai Tesque	Cat# 15838-45
Bacto™ Agar	BD	Cat# 214010
Competent Quick DH5α	Toyobo	Cat# DNA-913
Ampicillin Sodium Salt	Nacalai Tesque	Cat# 02739-74
QIAGEN Plasmid Midi kit	QIAGEN	Cat# 12145
DNA Ligation Kit Ver.2.1	Takara	Cat# 6022
Viral Production Medium	Gibco	Cat# A4817901
AAV-MAX Transfection Kit	Gibco	Cat# A50515
Violamo Disposable Sterilized Erlenmeyer Flask 125 mL Bent Filter Cap	VIOLAMO	Cat# SEF125V
Salt Active Nuclease (SAN)	Sigma-Aldrich	Cat# SRE0015
PEG 8000	Promega	Cat# V3011
OptiPrep	Serumwerk Bernburg AG	Cat# 1893
Type 70 Ti	Beckman Coulter	Cat# 337922
OptiSeal Tube	Beckman Coulter	Cat# 361625
Vivaspin® 20 Centrifugal Concentrator Polyethersulfone	Sartorius	Cat# VS2041

**Deposited data**		

Microarray gene expression data (Dnmt3a-Tg)	This paper	Gene Expression Omnibus: GSE252407
Microarray gene expression data (Dnmt3a-Tg)	This paper	Gene Expression Omnibus: GSE244336
Whole genome bisulfite sequencing data	This paper	Gene Expression Omnibus: GSE244432
Whole genome bisulfite sequencing data	This paper	Gene Expression Omnibus: GSE262342

**Experimental models: Cell lines**		

Plat-E cells	CBL	Cat# VPK-300
Primary myoblast	This paper	N/A
Viral Production Cells 2.0	Thermo Fisher	Cat# A49784

**Experimental models: Organisms/strains**		

Mouse: C57BL/6J	Shimizu Laboratory Supplies	–
Mouse: C57BL/6J	CLEA	–
Mouse: HSA-Dnmt3a-Tg	Kamei Lab	N/A
Mouse: Dnmt3a^flox/flox^;HSA-Cre Tg	Kamei Lab	N/A

**Oligonucleotides**		

Primers for genotyping	This paper	Table S9
Primers for qRT-PCR	This paper	Table S10

**Recombinant DNA**		

pMX-flag-GFP	This paper	N/A
pMX-flag-Dnmt3a	This paper	N/A

**Software and algorithms**		

Image J	NIH	https://imagej.nih.gov/ij/download.html
BZ-X800 Viewer	Keyence	–
BZ-X800 Analyzer	Keyence	–
ImageLab software	Bio-Rad	–
STRING: functional protein association networks	Szklarczyk et al.^54^	https://string-db.org
Cytoscape	Shannon et al.^107^	https://cytoscape.org
DAVID Bioinformatics Resources	LHRI	https://david.ncifcrf.gov/
Cluster 3.0	de Hoon et al.^108^	http://bonsai.hgc.jp/∼mdehoon/software/cluster/
Java TreeView	Saldanha et al.^109^	https://jtreeview.sourceforge.net
Integrative Genomics Viewer (IGV)_2.16.2	Robinson et al.^110^	https://igv.org
ChIP-Atlas	Zou et al.^111^	https://chip-atlas.org
Adobe Illustrator	Adobe	https://www.adobe.com
RNAseqChef	Etoh and Nakao^112^	https://imeg-ku.shinyapps.io/RNAseqChef/
R system	R Core Team	https://cran.r-project.org
Programming environment of R: RStudio	RStudio Team	https://www.rstudio.com
GSEA software	Subramanian et al.^113^	https://www.gsea-msigdb.org/gsea/index.jsp
Excel	Microsoft	https://www.microsoft.com/en-us/microsoft-365/excel
Prism 9 software	GraphPad	https://www.graphpad.com/
Venn Diagrams	Bioinformatics & Evolutionary Genomics	http://bioinformatics.psb.ugent.be/webtools/Venn/
BioRender: Scientific Image and Illustration Software was used for creating Graphical Abstract, Figures 1A, 5A, 5I, 7A, 8A, and 9D	N/A	https://www.biorender.com
Primer Express 2.0	Thermo Fisher	–
Primer-BLAST	NIH	https://www.ncbi.nlm.nih.gov/tools/primer-blast/
BLAST	NIH	https://blast.ncbi.nlm.nih.gov/Blast.cgi
BMap	N/A	http://itolab.med.kyushu-u.ac.jp/BMap/index.html

**Other**		

Treadmill	Muromachi	Cat# MK-690S/4M
Grip strength meter	Melquest	Cat# GPM-101B
Mass Spectrometer for Respiratory Analysis	ARCO System	Cat# ARCO-2000
HiSeq X Ten	Illumina	–
NovaSeq X	Illumina	–
Agilent Microarray Scanner	Agilent Technologies	Cat# G2565CA
CFX Connect Real-Time PCR Detection System	Bio-Rad	Cat# 1855201J1
ChemiDoc XRS+ Imaging System	Bio-Rad	Cat# 1708265J1
NanoDrop Lite Plus	Thermo Fisher	Cat# NDLPLUSGL
BZ-X800 fluorescence microscope	Keyence	–
Glutest Ai	Sanwa Kagaku	Cat# 086533218
CellShake CS-LR	TAITEC	Cat# 0081704-000
Optima™ XPN-90	Beckman Coulter	Cat# A94468

### Experimental model and study participant details

#### Animal experiments

Animal experiments were performed with the approval of the Institutional Animal Care and Use Committees of Kyoto Prefectural University. The study protocol was approved by the committees (No. KPU260407, review board: Dr. Yasuhiro Tsukamoto). Mice were cared for according to the National Institutes of Health (NIH) Guide for the Care and Use of Laboratory Animals. All other experiments were performed according to institutional guidelines. Mice were housed at a constant temperature of 24°C under artificial light (12-h light/dark cycle) with *ad libitum* access to food (CE-2, CLEA, Japan) and water, unless otherwise indicated. Sex- and age-matched mice were used in all experiments; mice sex and age at the time of the experiment are given in the method details section.

#### Dnmt3a overexpression experiment using Dnmt3a-Tg mice

Skeletal muscle-specific Dnmt3a transgenic (Dnmt3a-Tg) mice were generated using the human skeletal muscle α-actin (HSA) promoter^114^ provided by Drs. E. D. Hardeman and K. Guven (Children’s Medical Research Institute, Australia). Mouse Dnmt3a1 cDNA was prepared, as described.^115^ The transgene was excised from agarose gel and purified for injection (2 ng/μl). Fertilized eggs were collected from C57BL/6 females crossed with C57BL/6 males and microinjected at Japan SLC Inc. (Hamamatsu, Japan). All of the Dnmt3a-Tg mice and their WT littermates were generated by in-house mating of Dnmt3a-Tg male mice and C57BL/6J female mice (CLEA). The genotypes of the mice were confirmed by genotyping using HSA primers (Table S9). Excised tissues were weighed and rapidly frozen using liquid nitrogen or frozen in isopentane, cooled in liquid nitrogen.

#### MyoAAV-mediated Dnmt3a overexpression experiment

Male C57BL/6J mice at 2 or 24 months of age were randomly assigned to control and Dnmt3a overexpression mice. Excised tissues were weighed and rapidly frozen using liquid nitrogen or frozen in isopentane, cooled in liquid nitrogen. MyoAAV-injected mice were subjected to a treadmill running test before euthanasia and tissue collection. As a limitation of the generalizability of the study, only male mice were used for AAV experiments in this study.

#### Dnmt3a knockout experiment using Dnmt3a-KO mice

Skeletal muscle-specific Dnmt3a knockout (Dnmt3a-KO) mice were generated, as described.^41^ The genotypes of the mice were confirmed by genotyping using Cre primers (Table S9). Excised tissues were weighed and rapidly frozen using liquid nitrogen or frozen in isopentane, cooled in liquid nitrogen. Dnmt3a-KO and age-matched WT mice (male, 10 months old in Figures 9A–9C, 9K, and 9L) were subjected to a treadmill running test before euthanasia and tissue collection. As a limitation of the generalizability of the study, only male mice were used for Dnmt3a-KO experiment.

#### Cell lines and cultures

##### Plat-E cells

Plat-E cells (retrovirus packaging cells) were cultured in 10-cm dishes in Dulbecco's Modified Eagle Medium (DMEM) supplemented with 10% FBS to 90%–100% confluence and transfected with 6 μg of pMX-GFP or pMX-Dnmt3a retroviral vectors using Lipofectamine 2000. Six hours after transfection, the medium was replaced with DMEM supplemented with 10% FBS. At 48 h after transfection, the viral supernatant collected from cultured Plat-E cells was filtered through a 0.45-μm sterilization filter, and polybrene was added at a final concentration of 5 μg/ml. The cells are authenticated as having been purchased from CosmoBio Inc. and their identity has been verified and shown by the company to be free of contamination from other cell lines and mycoplasma. The Plat-E cells used were confirmed to be mycoplasma-free using MycoStrip (InvivoGen, #rep-mys-10). Using Plat-E cells, we have confirmed that functional proteins are expressed as expected in overexpression experiments.

##### Mouse primary cells

Mouse primary cells were isolated using single myofiber method, as previously described.^116^ In brief, EDL and soleus muscles were excised from 8-week-old C57BL/6J male mice and digested in 0.2% type I collagenase, dissolved in GlutaMAX DMEM, in a shaking incubator (50 rpm, 37°C) for 105 and 120 min, respectively. The collected myofibers were carefully selected to avoid contaminating other cells such as fibroblasts. Satellite cells were harvested from isolated single myofibers treated with Accutase for 10 min. To prevent premature differentiation and given the unique property of satellite cells to proliferate better in glucose-free media,^116^ we cultured the isolated satellite cell-derived myoblasts in glucose-free DMEM supplemented with 30% FBS, 1% chicken embryo extract, 1% GlutaMAX, 1% penicillin–streptomycin, and 10 ng/ml basic fibroblast growth factor on 15-cm dishes coated with Matrigel. Primary myotubes stably overexpressing Dnmt3a and GFP were prepared using retrovirus supernatant, as described.^91^ Briefly, satellite cell-derived myoblasts were infected with viral supernatant and growth medium (1:1 ratio). Two days after infection, the cells were selected two times with puromycin (3 μg/ml). Puromycin-selected satellite cell-derived myoblasts were plated in Matrigel-coated 12- or 24-well plates. After day 1, the cells were differentiated into myotubes in differentiation medium (GlutaMAX DMEM supplemented with 5% HS, 1% penicillin–streptomycin) for 3 d. Isolated satellite cell-derived myoblasts are morphologically confirmed by normal differentiation into myotubes when induced in myogenic differentiation medium. We were very careful to avoid contamination with other cells, but we recognize that this is a limitation of our study that cannot be completely ruled out. As a limitation of the generalizability of the study, only male mice were used for isolation of mouse primary cells.

##### Viral Production Cells 2.0

Viral Production Cells 2.0 (Thermo Fisher Scientific) were cultured in Viral Production Medium (Gibco) supplemented with 4 mM GlutaMAX Supplement (Gibco). The cells are authenticated because they were purchased from Thermo Fisher. Although we have not yet tested Viral Production Cells 2.0 for mycoplasma, we have confirmed that functional proteins are expressed as expected in overexpression experiments using Viral Production Cells 2.0.

### Method details

#### Quantitative real-time PCR analysis

Total RNA was prepared from snap-frozen tissues and primary myotubes using TRIzol reagent. Snap-frozen tissues were homogenized using Tissue Lyser II (Qiagen, Hilden, Germany) at 30 Hz for 3 min per cycle (three cycles). The supernatant was separated by centrifugation at 15,000 × *g* for 10 min at 4°C. Total RNA was extracted with TRIzol/chloroform (5:1) and precipitated with 100% isopropanol. The RNA precipitate was dissolved in distilled water. RNA concentration and purity were determined by using NanoDrop Lite Plus. cDNA was synthesized from 500 ng of total RNA using ReverTra Ace qPCR RT Master Mix with gDNA Remover. mRNA expression levels were measured on a CFX Connect Real-Time PCR Detection System using Thunderbird Next SYBR qPCR Mix. Data analysis was conducted using the ΔΔCt method. All data were normalized with 36B4, 18S, and β-actin expression. The primers used are shown in Table S10. The ages of Dnmt3a-Tg and WT mice analyzed for qPCR were as follows: Figure 1B; 3-month-old female mice, Figures 2D, 2E, 2J, 6C, and 6E; 3-month-old male mice, Figures 3K and 3L; 3-month-old male mice and 2- and 31-month-old male C57BL/6J mice, Figures 4O and 4P; 3- and 26-month-old female mice, Figure 6K; 2-month-old female mice, Figures 7D and 7E; 3-month-old female mice, Figures 8C and 8L–8O; 3, 4.5 or 25-month-old male mice, and Figures 9A and 9L; 10-month-old male mice.

#### Western blot analysis

Tissue samples (gastrocnemius muscle, TA muscle, quadriceps muscle, and liver) were homogenized using Tissue Lyser II (Qiagen, Hilden, Germany) at 30 Hz for 3 min per cycle (at least three cycles) in Radioimmunoprecipitation (RIPA) Lysis Buffer (50 mM Tris-HCl [pH 7.4], 150 mM NaCl, 0.25% deoxycholic acid, 1% NP-40, and 1 mM EDTA) supplemented with 1% protease inhibitor cocktail, 1 mM sodium orthovanadate, and 1 mM phenylmethylsulfonyl fluoride. The supernatant was separated by centrifugation at 15,000 × *g* for 15 min at 4°C. Protein concentration was determined using a BCA protein assay kit. Another aliquot was mixed with Sample Buffer Solution (2ME-), supplemented with dithiothreitol at a final concentration of 45 mM, and heated at 97°C for 5 min. Samples for OXPHOS complex protein detection were not heated according to the manufacturer’s instructions. Equal amounts of protein (20 μg or 15 μg) from each sample were subjected to SDS-PAGE on 10% or 15% precast polyacrylamide gels (e-PAGEL) and transferred to a nitrocellulose membrane or PVDF membrane. The membranes were blocked with TBST supplemented with 5% nonfat dry milk or skim milk for 1 h. Immunoblotting was performed with primary antibodies overnight at 4°C, followed by HRP-conjugated secondary antibodies for 1 h at room temperature. Blots were detected using ECL Prime Western Blotting Detection Reagent, and images were captured using a ChemiDoc XRS+ imaging system. Images were analyzed using ImageLab software. The ages of Dnmt3a-Tg and WT mice analyzed for western blot analysis were as follows: Figure 1A; 2-month-old male mice, Figure 3E; 3-month-old female mice, Figure 4N, Q; 3- and 26-month-old female mice, Figure 6H; 2-month-old female mice, Figures 8B and 8J; 3 or 4.5-month-old male mice, and Figure 9B; 10-month-old male mice.

#### Glucose tolerance test and insulin tolerance test

Dnmt3a-Tg and age-matched WT mice (male, 7 months old) were used for analysis. Glucose tolerance test and insulin tolerance test were performed, as described.^41^ Briefly, for insulin tolerance test, insulin (0.75 mU/g of body weight) was administered intraperitoneally to fed mice; for glucose tolerance test, D-glucose (1 mg/g of body weight) was administered orally to overnight-fasted mice.

#### High-fat diet studies

Dnmt3a-Tg and age-matched WT mice (male, 8 weeks old) were used for analysis. Dnmt3a-Tg and WT mice were fed with either HFD (60 kcal% calories from fat, D12492, Research Diet) or CE2 for 10 weeks. Body weights were measured weekly.

#### Genome-wide analysis of DNA methylation by MIAMI and PBAT

Dnmt3a-Tg and age-matched WT mice (male, 8 weeks old) were used for MIAMI analysis. Muscle genomic DNA from Dnmt3a-Tg and WT mice was extracted with a standard proteinase K method. Gastrocnemius muscle was incubated with DNA digestion buffer (20 mM Tris [pH 8.0], 5 mM EDTA, 400 mM NaCl, 1% SDS, and 0.2 mg/ml proteinase K) overnight at 55°C. Genomic DNA was extracted with neutralized phenol/chloroform (1:1) and precipitated with 100% ethanol. The DNA precipitate was dissolved in TE buffer. Microarray-based integrated analysis of methylation by isoschizomers (MIAMI), a genome-wide analysis of DNA methylation using a gene array and methylation-sensitive restriction enzymes, was performed, as described^117^ by using our protocol (MIAMI protocol).^53^ Briefly, genomic DNA from the two samples was digested using methylation-sensitive *Hpa*II and methylation-insensitive *Msp*I, followed by adaptor ligation and PCR amplification. Amplified DNA from one sample was labeled with Cy3 and the other with Cy5 and then cohybridized in the gene arrays using Oligo aCGH/ChIP-on-chip Hybridization Kit. To describe the difference in DNA methylation between samples, scatter plot graphs were depicted with log-transformed values of *Hpa*II signal differences (horizontal axis) and *Msp*I signal differences (vertical axis) (Figure S1D). For each probe, the difference in the *Hpa*II/*Msp*I signal was used to determine the degree of DNA methylation, with the following criteria for DNA methylation difference: Values <0.714 and >1.3 denoted DNA hypomethylation and hypermethylation, respectively.^118^ GO enrichment analysis was performed and PPI networks were constructed using the STRING database,^54^ utilizing a list of 334 genes hypermethylated in Dnmt3a-Tg mice compared with WT mice. The PPI networks were further modeled using Cytoscape software.^107^ The color and circle size of nodes were mapped to the degree of genes.

For further methylome analysis, PBAT-mediated whole-genome bisulfite sequencing was performed, as described.^55^^,^^56^ TA muscles of Dnmt3a-Tg and age-matched WT mice (male, 3 months old), TA muscles of Dnmt3a-KO and age-matched WT (male, 3 months old), and gastrocnemius muscles of Dnmt3a-Tg and age-matched WT mice (female, young; 3 months old, old; 26 months old) mice were used for PBAT analysis. For preparation of PBAT libraries, genomic DNAs were extracted from muscle tissue. Sequencing was performed by Macrogen Japan Corp. (Kyoto, Japan) using HiSeq X Ten for TA muscles and Novaseq X for gastrocnemius muscles, and the reads were mapped to the mouse mm10 reference genome using BMap with default parameter settings. Methylation levels of CG sites were calculated for only those covered by ≥10 reads and ≥5 reads for TA and gastrocnemius muscles, respectively. Methylation levels of 100-kb, 50-kb, 10-kb, and 5-kb sliding windows, promoters (<2 kb upstream of transcriptional start site [TSS]), gene bodies, CG islands (CGIs), and TSS-proximal regions were determined by averaging the methylation levels of CG sites in individual features and analyzed using two unsupervised classification methods: hierarchical clustering and PCA. Differentially methylated regions (DMRs) were identified using metilene^119^ with default parameters. DMRs for TA muscles were filtered with *p* value (2D KS) < 0.01 and methylation differences > 20%. In Figure S15C, DMRs for TA muscles were also filtered with *p* value (2D KS) < 0.05 and methylation differences > 10%. DMRs for gastrocnemius muscles were filtered with *p* value (2D KS) < 0.05 and methylation differences > 10%. The nearest neighbor gene of a DMR was defined as the gene associated with the DMR. Hierarchical clustering of DMR methylation values transformed into z-scores was performed using Cluster 3.0^108^ with a correlation with a complete linkage and visualized using Java TreeView.^109^ Sex chromosomes were excluded in the PBAT analysis. The Integrative Genomics Viewer (IGV) was used to visualize the DNA methylation changes in the genomic regions closest to the genes of interest mapped to the mouse mm10 reference genome. ChIP-Atlas was used to display the previously reported gastrocnemius histone ChIP-seq peaks and leg muscle ATAC-seq peaks with a significance threshold of 50. GO enrichment analysis and KEGG pathway analysis were performed using the comprehensive bioinformatics resource functional annotation tool DAVID^120^ using a list of DMRG. The results of GO analysis and KEGG pathway analysis were plotted using ggplot2 and dplyr libraries in R (v.4.3.2).

#### cDNA microarray analysis

RNA was isolated from gastrocnemius muscles of Dnmt3a-Tg and age-matched WT mice (female, young; 3 months old, old; 26 months old). Purification of extracted RNA was performed using an RNeasy Mini Kit. RNA purity was determined by measuring ultraviolet absorbance at 260 and 280 nm, to confirm absence of protein contamination. Each sample was labeled with cyanine 3-CTP using a Low Input Quick Amp Labeling Kit and hybridized to the Agilent whole mouse genome microarray (8 × 60K). Scanning of the hybridized microarray slides was performed using an Agilent Microarray Scanner. Signal interpretation and data analysis were performed according to the manufacturer’s instructions. Analysis was performed in part using RNAseqChef,^112^ a web-based platform for systemic transcriptome analysis, using DESeq2 for pair-wise DEG detection with Benjamini-Hochberg adjusted *p* value < 0.05 in young (3-month-old) Dnmt3a-Tg compared to age-matched WT muscles (Figures 1K–1M, 3G, and 3H), using EBSeq for 3 conditions DEG in young (3-month-old) and older (26-month-old) WT and young (3-month-old) Dnmt3a-Tg muscles (Figure S7E) and using DESeq2 for multi-DEG analysis in young (3-month-old) and older (26-month-old) WT and age-matched Dnmt3a-Tg muscles (Figures 4G–4I). The plots obtained in RNAseqChef were partially replotted using ggplot2 and dplyr libraries in R (v.4.3.2) for ease of viewing. Hierarchical clustering of expression values transformed into z-scores was in part performed using Cluster 3.0^108^ with a correlation with a complete linkage and visualized using Java TreeView.^109^ Networks associated with multiple signaling or metabolic pathways were constructed using the STRING database and modeled using Cytoscape software (Figures 2B, 2C, 6A, S1H, S7C, and S12A). The color and circle size of nodes were mapped to fold-change values.

#### Functional enrichment analysis and GSEA of microarray data

GO enrichment analysis and KEGG pathway analysis were performed using the DAVID^120^ using a list of 169 genes with increased expression in 3-month-old Dnmt3a-Tg muscle compared with age-matched WT muscle (fold change > 1.5, FDR < 0.05 and average signal value; WT average > 500). The results of GO analysis and KEGG pathway analysis were plotted using ggplot2 and dplyr libraries in R (v.4.3.2). GSEA of Dnmt3a-regulated genes was performed using GSEA software (v.4.3.2)^113^ using gene sets from the Molecular Signatures Database (MSigDB) “hallmark gene sets” and “ontology gene sets” with default settings (1,000 permutations for gene sets, Signal2Noise metric for ranking genes). GSEA of Dnmt3a-regulated genes was also performed using a senescence-associated gene set (SenMayo),^77^ and gene sets of KEGG_Chemokine Signaling Pathway (M4844), KEGG_Cytokine-Cytokine Receptor Interaction (M9809), GOBP_NADH Dehydrogenase complex assembly (M22615), Muscle FoxO Signaling (Table S7),^91^ and Putative FoxO1 Target in Muscle (Table S7).^91^ Network visualization and clustering of GSEA analysis were performed using GSEA software, Cytoscape, and EnrichmentMap application (v.3.3.6), AutoAnnotate application (v.1.4.1), clusterMaker2 application (v.2.3.4) and WordCloud application (v.3.1.4) to visualize enriched gene sets at a FDR < 0.05 with the Jaccard overlap combined coefficient > 0.375 with combined constant = 0.5, where automatically WordCloud-generated theme names were in part carefully renamed as previously described.^121^ Red and blue nodes represent positive and negative NES, and circle size of nodes were mapped to gene set size.

#### Construction of PPI networks and identification of hub genes

PPI networks were constructed using the STRING database and further modeled using Cytoscape. Top 2000 genes with increased expression in 3-month-old Dnmt3a-Tg muscle compared with age-matched WT muscle (fold change > 2, FDR < 0.05) were submitted to the STRING database. Top 20 putative hub genes with high degree were plotted using ggplot2 and dplyr libraries in R (v.4.3.2).

#### Immunohistochemical analysis

Immunohistochemical analysis was performed on the TA and soleus muscles of Dnmt3a-Tg and age-matched WT mice (male, 5 months old) and Dnmt3a-KO and age-matched Dnmt3a^flox/flox^ (WT) mice (male, 10 months old), as described.^122^ MyoAAV-injected TA muscles were also subjected to immunohistochemical analysis. Briefly, excised muscles were frozen in isopentane cooled in liquid nitrogen. Muscle cryosections (8 or 10 μm) were either permeabilized with 0.5% Triton X-100 in PBS for 15 min and blocked with 1% MOM blocking reagent in PBST for 1 h, or fixed with 2% PFA in PBS for 10 min, permeabilized and blocked with 0.5% Triton X-100 and 1% MOM blocking reagent in PBS for 1 h. Immunostaining was performed with primary antibodies: mouse anti-type IIa myosin heavy chain (MyHC; SC-71), mouse anti-type IIb MyHC (BF-F3), and mouse anti-type I MyHC (BA-D5) antibodies, as well as rat anti-laminin α2 antibody, overnight at 4°C, followed by fluorescence-conjugated secondary antibodies for 1.5 h at room temperature. The sections were mounted using Fluoro-KEEPER Antifade Reagent with or without DAPI or Dako Fluorescence Mounting Medium. Immunostained images were optimized globally and assembled into figures using a BZ-X800 fluorescence microscope. Muscle cross-sectional area (CSA) was measured by using BZ-X800 Analyzer software. In Figures 8I and S13F, the CSA of type IIb and IIa myofibers was measured based on large 100 myofibers (84 myofibers in one section only) in the deep TA regions. Number of fibers and central nuclei were counted using ImageJ software. Mitochondrial succinate dehydrogenase staining of muscle sections (gastrocnemius and soleus) from Dnmt3a-Tg and age-matched WT mice (male, 3 months old) was performed by Applied Medical Research Corp. (Osaka, Japan).

#### CTX injection experiment

Dnmt3a-Tg and age-matched WT mice (4-month-old) were anesthetized, and hair was removed from their lower leg using a depilatory cream (Epilat, Kracie). To induce muscle injury, 50 μl of 10 μM CTX was injected into the TA muscles of anesthetized mice. CTX-injected and noninjected (intact) muscles were harvested for transverse sectioning and immunostaining 2 weeks after treatment.

#### Plasmid constructs: Dnmt3a expression vector

To generate the pMX-Dnmt3a retroviral vector, pcDNA-Dnmt3a plasmid, which was generated by cloning murine Dnmt3a1 cDNA into pcDNA™6/V5-His A, B and C vector (Thermo-Fisher), was used as a template for Dnmt3a cDNA sequence. Dnmt3a cDNA was cloned into pMX retroviral vector with a blunt end. The plasmid construct was confirmed by the Fasmac sequencing service (Fasmac, Kanagawa, Japan).

#### Immunofluorescence staining

For immunofluorescence staining, primary myotubes were fixed with 2% paraformaldehyde in GlutaMAX DMEM for 10 min. The cells were permeabilized and simultaneously blocked with 0.3% Triton X-100 in PBS supplemented with 10% goat serum for 20 min. Immunostaining was performed with primary antibodies to MyHC (MF-20) in 1% BSA in PBS overnight at 4°C, followed by fluorescence-conjugated secondary antibodies for 1 h at room temperature. The cells were mounted with DAPI Fluoromount-G. Image detection was performed using a BZ-X800 fluorescence microscope.

#### Measurements of oxygen consumption and respiratory exchange ratio

Dnmt3a-Tg and age-matched WT mice (male, 12–14 weeks old) were housed in individual metabolic chambers coupled to a mass spectrometer (ARCO-2000) with *ad libitum* access to food (CE-2) and water. The gas analysis system used to measure oxygen consumption (VO_2_), carbon dioxide production (VCO_2_), respiratory exchange ratio (RER), ratio of volume of CO_2_ produced to volume of O_2_ consumed (VCO_2_/VO_2_), carbohydrate oxidation (CHO), and lipid oxidation (FAT) was as described.^123^ An RER value close to 0.7 indicates exclusive fat utilization, and a value close to 1.0 indicates exclusive carbohydrate utilization.

#### Measurement of mitochondrial DNA copy number

Dnmt3a-Tg and age-matched WT mice (male, 3 months old) were used for analysis. Muscle genomic DNA from Dnmt3a-Tg and WT mice was extracted using a standard proteinase K method. Gastrocnemius muscle was incubated with DNA digestion buffer (20 mM Tris [pH 8.0], 5 mM EDTA, 400 mM NaCl, 1% SDS, and 0.2 mg/ml proteinase K) at 55°C overnight. Genomic DNA was extracted with neutralized phenol/chloroform (1:1), treated with ribonuclease A for 5 h at 37°C, and precipitated with 100% ethanol. The DNA precipitate was dissolved in TE buffer. Mitochondrial DNA (mtDNA) content was measured as mtDNA copy number normalized to the copy number of a gene in the nuclear genome (COX2/COX4 ratio), as described.^124^ The mitochondrial gene used for mtDNA estimation was cytochrome *c* oxidase subunit 2 (COX2). The copy number of COX2 was normalized to the copy number of a gene in the nuclear genome (COX4). Real-time PCR was used to estimate the copy number of specific genes.

#### Treadmill running test

Dnmt3a-Tg and age-matched WT mice (female, 7 weeks old in Figure S4J; female, 12 and 23 months old in Figures 4B and 4C), young (3-month-old) and older (29-month-old) WT mice (female, Figure 4B), and Dnmt3a-KO and age-matched WT mice (male, 10 months old in Figure 9K) were subjected to a treadmill running test (MK-690S/4M). MyoAAV-injected mice were also subjected to a treadmill running test before euthanasia. Mice were acclimatized prior to the test to familiarize them with the treadmill (Figures 4B, 4C, 9K, S13B, and S13C). On the day of acclimatization, mice were placed on the treadmill for 5 min, walked at 5 m/min for 30 min 1 week before the test (23 months old in Figures 4B and 4C) or walked at 5 m/min, and the speed was increased by 1/min up to 10 m/min 1 d before the test (3, 12 and 29 months old in Figures 4B, 4C, 9K, S13B, and S13C). An electrical stimulation grid was set to 0.5 mA and the slope was set to 0 degrees. On the day of testing, mice were placed on the treadmill and the belt speed was started at 5 m/min for 5 min. Mice were forced to run at 10 m/min for 25 min, and the speed was increased by 3 m/min every 3 min until the mice remained on the electrical stimulation grid for approximately 5 s. During the test, running distance, running time, and maximum running speed were measured. Immature 7-week-old mice were forced to run at 10 m/min for 5 min; speed was increased by 2 m/min every 2 min up to 20 m/min (Figure S4J). One hour after the start of treadmill running, speed was increased by 2 m/min every 2 min up to 25 m/min. In total, treadmill running continued for 2 h or until the mice were exhausted.

#### Grip strength test

Muscle strength was measured using a grip strength meter (GPM-101B). Dnmt3a-Tg and age-matched WT mice (male 3 months old, female 3 months old, and female 12 months old) were held by the tail, allowed to grip a forelimb grip with the front paws, and pulled backward until the grip was released. This measurement was repeated for three sets by a different person, and the highest value was noted.

#### Resistance exercise (ladder climbing)

According to a previous study, resistance exercise was performed 2–3 times a week for 5 months using a climbing ladder made of a barbecue net (length, 80 cm; grid, 1.3 cm; inclination, 80°).^125^ The first week was an acclimatization period with six and eight rounds of ladder climbing at 50% of the ‘maximum lifting weight’ at 1 min intervals. From the second week, the mice (3-month-old female WT and Dnmt3a-Tg mice) underwent resistance exercise with nuts (weight) attached to the tail at 70% of the 'maximum lifting weight' and 12 rounds of ladder climbing were performed at 1 minute intervals. As for the mouse that stopped climbing, we touched its buttocks to get it to climb. If the mouse was unable to climb, the weight was reduced to allow it to climb.

#### AAVs preparation and injection

Mouse *Dnmt3a* was cloned into pAAV-MHCK7 and the sequence was confirmed by the GENEWIZ sequencing service (Azenta Life Sciences). The preparation of MyoAAVs was performed as previously described.^126^ On the day of transfection, 9×10^7^ cells of Viral Production Cells 2.0 (Thermo Fisher Scientific) were triple transfected with pAAV-MHCK7-GOI (pAAV-MHCK7-Dnmt3a or pAAV-MHCK7-empty), pUCmini-iCAP-MyoAAV.2A (created by replacement of the capsid coding sequence of pUCmini-iCAP-PHP.S with that of MyoAAV 2A^94^) and pHelper plasmid (TaKaRa). At 72 hours after transfection, medium and cells were collected and separated by centrifugation at 2000 × g for 15 min at room temperature. Cells were lysed with salt-active nuclease (Sigma) + SAN digestion buffer^127^ at 37°C for 1 hour. AAV in the culture media was precipitated with PEG 8000 solution by centrifugation at 4000 × g for 30 min at 4°C and resuspended with SAN digestion buffer. The resuspended PEG pellet was added to the cell lysate and treated with salt-active nuclease at 37°C for 30 minutes. After removing insoluble proteins by centrifugation, the supernatant was layered on iodixanol gradients (15%, 25%, 40%, and 60%) and subjected to ultracentrifugation at 350,000 × g for 2 h 30 min at 18°C. The 40% iodixanol layer containing the AAV particles was extracted. Buffer exchange to PBS was performed using Vivaspin 20, 100 kDa MWCO (sartorius). Viral titers were measured by qPCR using primers that bind within the SV40 polyadenylation signal of AAV vector (forward primer: TGGACAAACCACAACTAGAATGC; reverse primer: CCTCCCCCTGAACCTGAAAC). For injection of purified MyoAAV, 1.3 x 10^10^ vg (viral genome)/ 50 μL PBS was injected i.m. into anesthetized 2 or 24-month-old male C57BL/6J mice (per mouse: 6 sites (1 TA and 2 gastrocnemius muscle sites on both hind limbs). At 1 or 2.5 months after i.m. injection of MyoAAV, mice were euthanized and tissue samples were collected.

#### Fasting and refeeding of mice, and the calculation of tissue elasticity score (ElaS)

For the fasting experiment, Dnmt3a-Tg and age-matched WT mice (female, 2 months old) were fasted for 48 h in individual cages, whereas control mice were fed *ad libitum*. Skeletal muscle (gastrocnemius, quadriceps, tibialis anterior, extensor digitorum longus, and soleus) was harvested immediately after fasting. For the fasting and refeeding experiment, Dnmt3a-Tg and age-matched WT mice (female, 3 months old) were fasted for 48 h, or refed for 24 h after fasting in individual cages, while control mice were fed *ad libitum*. Blood glucose levels of Dnmt3a-Tg and WT mice were measured with Glutest Neo Sensor and Glutest Ai using blood collected from mouse tails. The Tissue Elasticity Score (ElaS) was calculated using an Excel function as follows:

• *Fed value X* = ‘Fed value’ / average of ‘Fasted value’ ∗100 - 100
• *Refed value Y* = ‘Refed value’ / average of ‘Fasted value’ ∗100 - 100
• *Value 1* = (*Refed value Y* -100 + Average of (*Fed value X* -100)) ∗min(*Refed value Y* -100,Average of (*Fed value X* -100))/max(*Refed value Y* -100,Average of (*Fed value X* -100))
• *Value 2* = IF(Y-100>0,*Value1*,-*Value1*)
• ElaS = IF(AND(ABS(*Refed value Y* -100)>Average of (*Fed value X* -100), *Refed value Y* -100<0),-*Value2*,*Value2*)

Here, ‘Fed value’, ‘Fasted value’ and ‘Refed value’ are the tissue weights of each fed, fasted and refed mice, respectively.

### Quantification and statistical analysis

Student’s two-tailed unpaired *t*-test was performed for comparisons between two groups. Comparisons of more groups were performed using one- or two-way analysis of variance (ANOVA) followed by Tukey’s *post hoc* test. All statistical analyses were performed using GraphPad Prism software, except for MIAMI, PBAT, and cDNA microarray analyses. Data were expressed as mean ± SEM. ∗*P* < 0.05, ∗∗*P* < 0.01, and ∗∗∗*P* < 0.001 were considered statistically significant. The statistical details of experiments can be found in each figure legend.

## Supplemental information

Document S1. Figures S1–S16 and Tables S9 and S10

Table S1. List of the differentially expressed genes (DEGs), the differentially methylated genes (DMGs), and the differentially methylated region-associated genes (DMRGs) in skeletal muscle of young skeletal-muscle-specific Dnmt3a-Tg mice compared to WT mice

Table S2. List of 169 genes whose expression levels were upregulated (fold change >1.5, FDR <0.05, and average signal value; WT average >500) in skeletal muscle of young (3-month-old) skeletal-muscle-specific Dnmt3a-Tg mice compared to age-matched WT mice

Table S3. List of top 2000 genes whose expression levels were upregulated (fold change >2, FDR <0.05) in skeletal muscle of young (3-month-old) skeletal muscle-specific Dnmt3a-Tg mice compared to age-matched WT mice

Table S4. List of genes whose expression levels were up- or downregulated (FDR <0.1) in skeletal muscle of 26-month-old WT mice compared to 3-month-old WT mice and genes whose expression levels were up- or downregulated (FDR <0.05) in skeletal muscle of 26-month-old Dnmt3a-Tg mice compared to 26-month-old WT mice

Table S5. List of the differentially methylated region-associated genes (DMRGs) in skeletal muscle of young and old WT and skeletal-muscle-specific Dnmt3a-Tg mice

Table S6. List of the differentially expressed genes (DEGs) and the differentially methylated region-associated genes (DMRGs) in skeletal muscle of young and old WT and skeletal-muscle-specific Dnmt3a-Tg mice

Table S7. Gene sets of “Muscle FoxO Signaling” and “Putative FoxO1 Target in Muscle” previously identified 91

Table S8. List of the differentially methylated region-associated genes (DMRGs) in skeletal muscle of WT and skeletal-muscle-specific Dnmt3a-KO mice and WT and skeletal-muscle-specific Dnmt3a-Tg mice
