# Supplementary material for: Dnmt3a overexpression disrupts skeletal muscle homeostasis, promotes an aging-like phenotype, and reduces metabolic elasticity
Source: iScience. 2025 Mar 3;28(4):112144. doi: 10.1016/j.isci.2025.112144 (PMC11937683; doi:10.1016/j.isci.2025.112144)
Supplement: Document S1. Figures S1–S16 and Tables S9 and S10 [file mmc1.pdf]

iScience, Volume *28*

Supplemental information

Dnmt3a overexpression disrupts skeletal muscle homeostasis, promotes an aging-like phenotype, and reduces metabolic elasticity

Mamoru Oyabu, Yuto Ohira, Mariko Fujita, Kiyoshi Yoshioka, Runa Kawaguchi, Atsushi Kubo, Yukino Hatazawa, Hinako Yukitoshi, Huascar Pedro Ortuste Quiroga, Naoki Horii, Fumihito Miura, Hiromitsu Araki, Masaki Okano, Izuho Hatada, Hitoshi Gotoh, Tatsuya Yoshizawa, So-ichiro Fukada, Yoshihiro Ogawa, Takashi Ito, Kengo Ishihara, Yusuke Ono, and Yasutomi Kamei

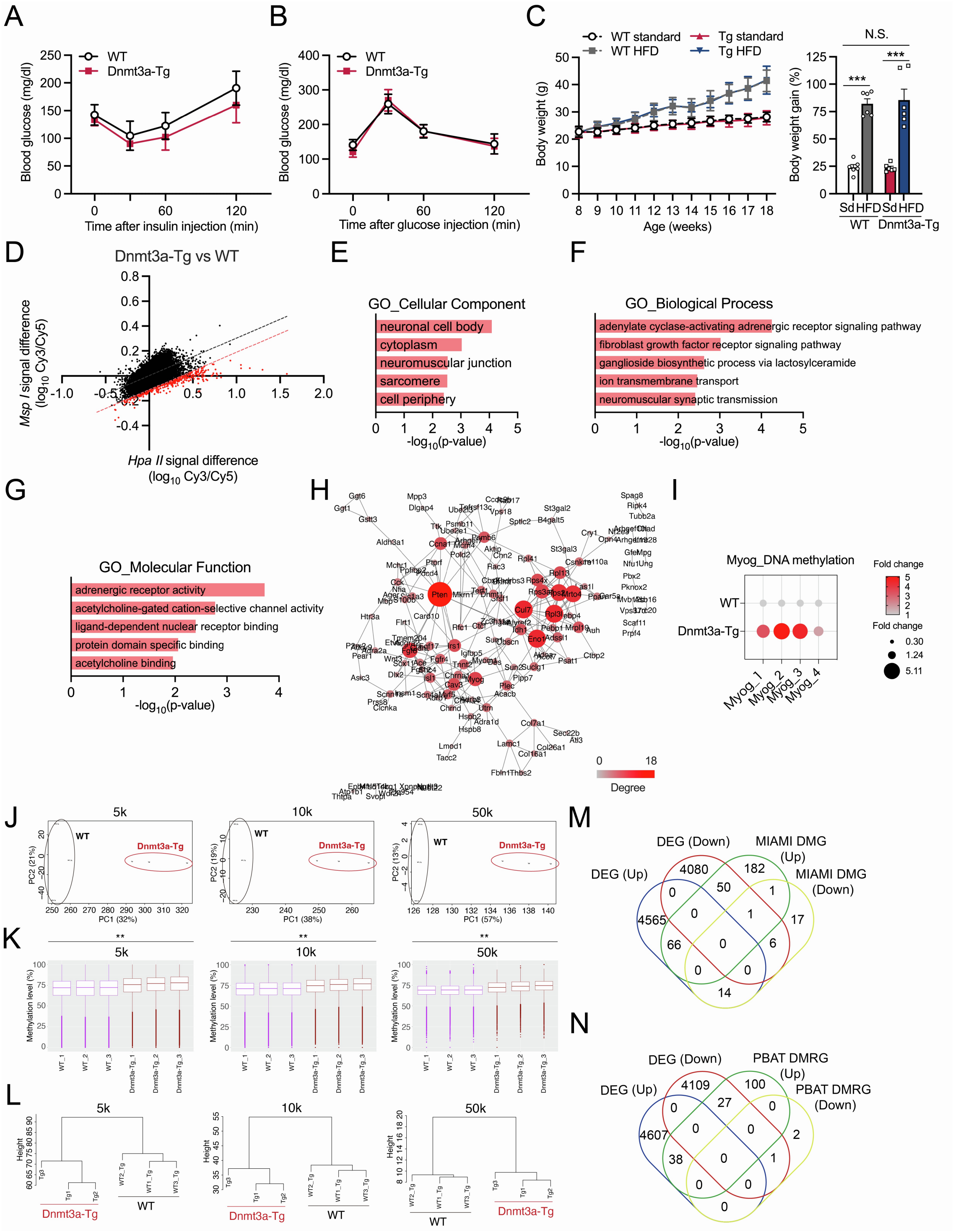

Figure S1. Phenotypes and DNA methylome analysis of Dnmt3a-Tg mice

(A-B) Insulin tolerance test (A) and glucose tolerance test (B) were conducted in WT and Dnmt3a-Tg mice (n = 7 mice/ group). (C) Chronological measurement of body weight and body weight gain of WT and Dnmt3a-Tg mice fed with standard diet or high-fat diet for 10 weeks (n = 6 mice/ group). (D) MIAMI analysis comparing gastrocnemius muscle harvested from WT and Dnmt3a-

Tg mice. Plots of log-transformed values of *Hpa*II (methylation-sensitive, horizontal axis) and *Msp*I (methylation-insensitive, vertical axis) signal difference between samples. Values of *Hpa*II signal difference/*Msp*I signal difference considered increased and decreased DNA methylation are >1.3 and <0.714, respectively. The regression line is in black and red lines is located $\log_{10}$ 1.3 of the horizontal distance from the regression line. Red plots are considered hypermethylated genes. (E-G) Gene ontology (GO) enrichment analysis in (E) cellular component, (F) biological process, and (G) molecular function of differentially methylated genes (DMGs) hypermethylated in Dnmt3a-Tg mice compared with WT mice, identified using the MIAMI method from gastrocnemius muscle. (H) The PPI network of DMGs upregulated in Dnmt3a-Tg muscle compared to WT muscle. 167 nodes and 286 edges were shown. The color and the circle size of nodes indicates the number of direct edges (degree). (I) Dot plot illustrating the increased DNA methylation of *Myog* gene at several CpG sites. (J) Principal component analysis of DNA methylation of 5-kb, 10-kb, and 50-kb sidling windows (n = 3 mice/group). (K) Boxplot of mean CpG methylation level of 5-kb, 10-kb, and 50-kb sidling windows (n = 3 mice/group). (L) Unsupervised hierarchical clustering using Euclidean distance across the sample set (n = 3 mice/group). (M, N) Venn diagram showing that DMGs and DMRGs by Dnmt3a expression partially matched the differentially expressed genes (DEGs) based on MIAMI (M) and PBAT (N) data. All data indicate mean ± SE. $^{**}P < 0.01$, $^{***}P < 0.001$. (C) One-way analysis of variance (ANOVA) followed by Tukey's post hoc test. (K) Student's two-tailed unpaired *t*-test.

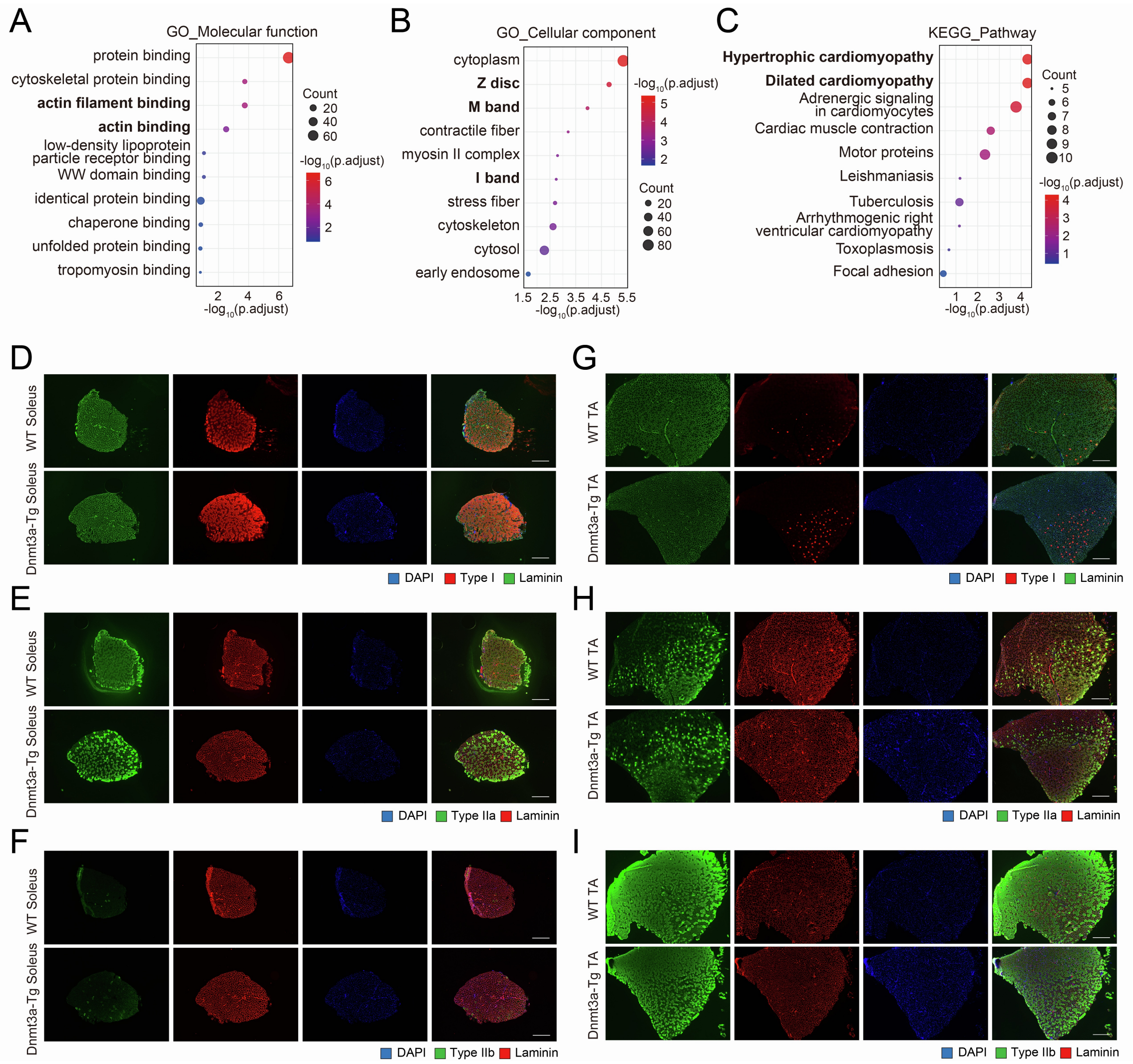

Figure S2. Overexpression of Dnmt3a affects the expression of genes involved in the muscle structural organization

(A-C) GO enrichment analysis in molecular function (A) and cellular component (B) and KEGG pathway analysis (C) of DEGs with relatively high basal expression (genes presumably expressed in myofibers) that had increased (FDR < 0.05, fold change > 1.5) expression in gastrocnemius muscle from Dnmt3a-Tg mice compared to WT mice. (D–I) Representative images of immunohistochemical staining of myosin heavy chain types, laminin, and DAPI (type I, red in Figure S2D, G; type IIa, green in Figure S2E, H; type IIb, green in Figure S2F, I) in soleus (D–F) and TA (G–I) muscle cross-section of WT and Dnmt3a-Tg mice. Scale bar = 500 μm.

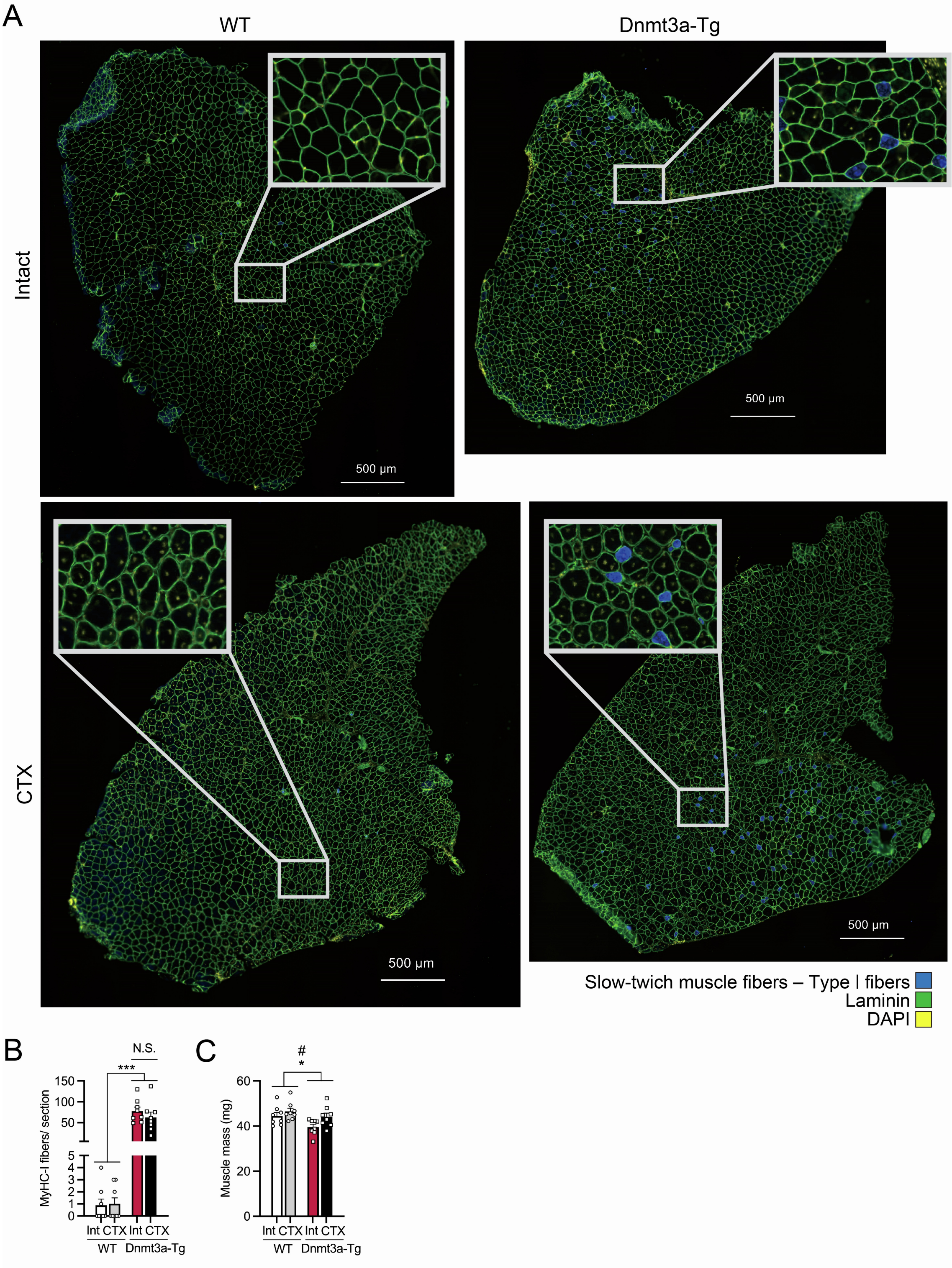

Figure S3. Type I myofiber formation in Dnmt3a-Tg mice is likely not dependent on muscle satellite cells

(A) Representative images of immunohistochemical staining of the type I myosin heavy chain, laminin and DAPI (type I, blue; laminin, green and DAPI, yellow) in intact and cardiotoxin (CTX)

injected TA muscle cross-section of WT and Dnmt3a-Tg mice. (B, C) Quantification of the number of type I myofibers (B) and TA muscle mass (C) in intact and CTX injected TA muscle of WT and Dnmt3a-Tg mice (n = 8 mice/ group). Scale bar = 500 μm. All data indicate mean ± SE. $^{*}P < 0.05$, $^{***}P < 0.001$. # indicates CTX effect. (B, C) Two-way analysis of variance (ANOVA) followed by Tukey's post hoc test.

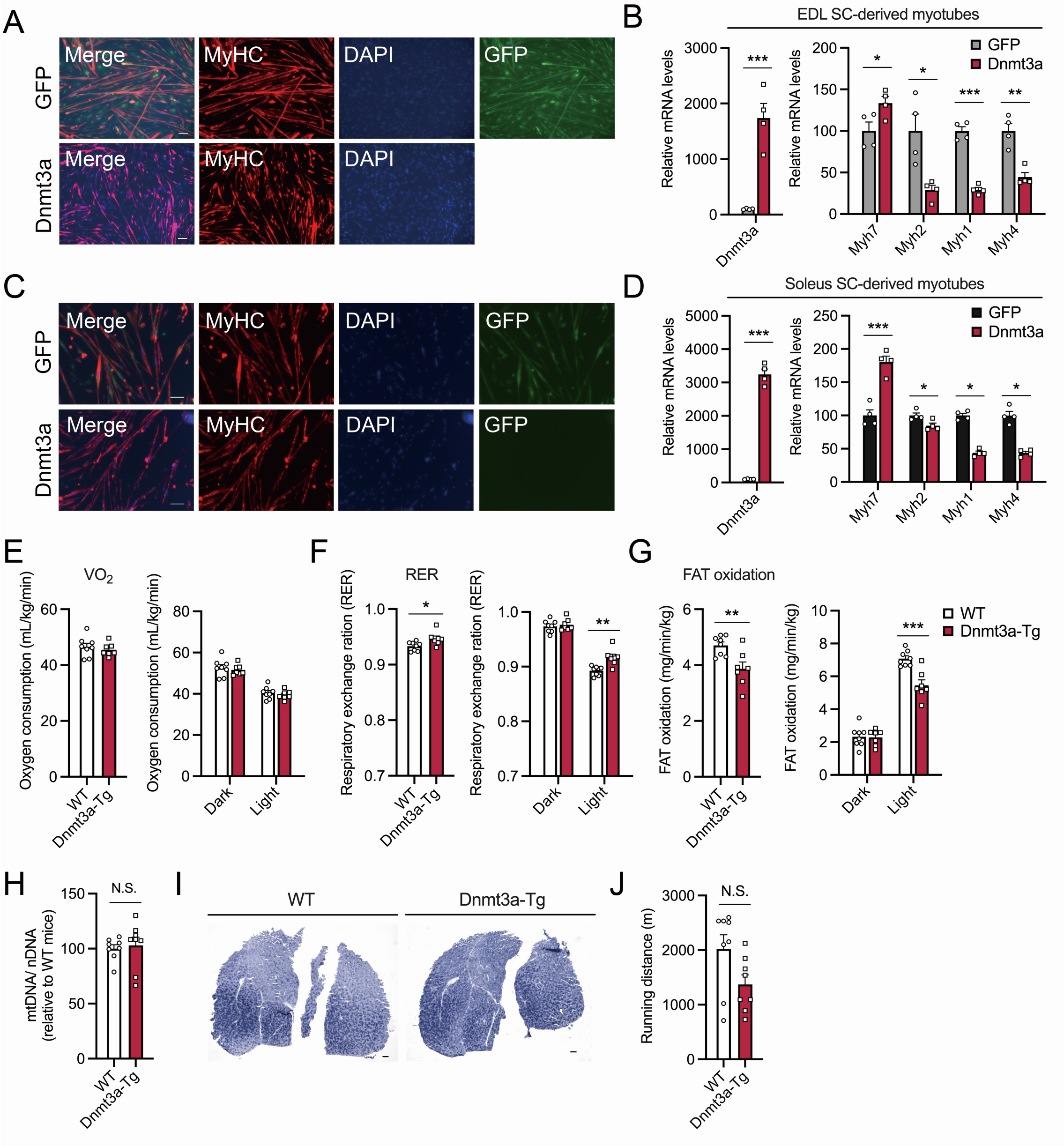

Figure S4. Sustained Dnmt3a overexpression results in muscle atrophy in vitro and dose not increase mitochondrial content, oxidative capacity and endurance exercise capacity in young mice

(A-D) Satellite cell-derived myoblasts collected from EDL (A-B) or Soleus (C-D) muscle of C57BL/6J mice were overexpressed with GFP or Dnmt3a using a retrovirus, after which the cells were induced to differentiate into myotubes for 3 days. (A) Representative images of immunofluorescence staining of the myosin heavy chains and DAPI (MyHC, red; GFP, green and DAPI, blue) in EDL-derived primary myotubes overexpressing GFP or Dnmt3a. Scale bar = 100 µm. (B) Relative mRNA expression of the *Dnmt3a* and myosin heavy chain genes in EDL-derived primary myotubes overexpressing GFP or Dnmt3a (n = 4 sets of cells per group). (C)

Representative images of immunofluorescence staining of the myosin heavy chains and DAPI (MyHC, red; GFP, green and DAPI, blue) in Soleus-derived primary myotubes overexpressing GFP or Dnmt3a. (D) Relative mRNA expression of the *Dnmt3a* and myosin heavy chain genes in Soleus-derived primary myotubes overexpressing GFP or Dnmt3a (n = 4 sets of cells per group). (E-G) The average values of oxygen consumption (E), respiratory exchange ratio (RER) (F) and FAT oxidation (G) in WT and Dnmt3a-Tg mice (n = 8 WT mice, n = 7 Dnmt3a-Tg mice). (H) Relative mitochondrial DNA copy number was calculated as the ratio of COX2 (mitochondrial) to COX4 (nuclear) genes as determined using real-time PCR for estimating mitochondrial DNA copy number in gastrocnemius muscle of WT and Dnmt3a-Tg mice (n = 8 mice/ group). (I) Representative SDH staining image in gastrocnemius and soleus muscle of WT and Dnmt3a-Tg mice. Scale bar = 200 μm. (J) Total running distance achieved by young WT and Dnmt3a-Tg mice in a treadmill running test (n = 8 mice/ group). All data indicate mean ± SE. * $P < 0.05$, ** $P < 0.01$, *** $P < 0.001$. (B, D, E, F, G, H and J) Student's two-tailed unpaired *t*-test.

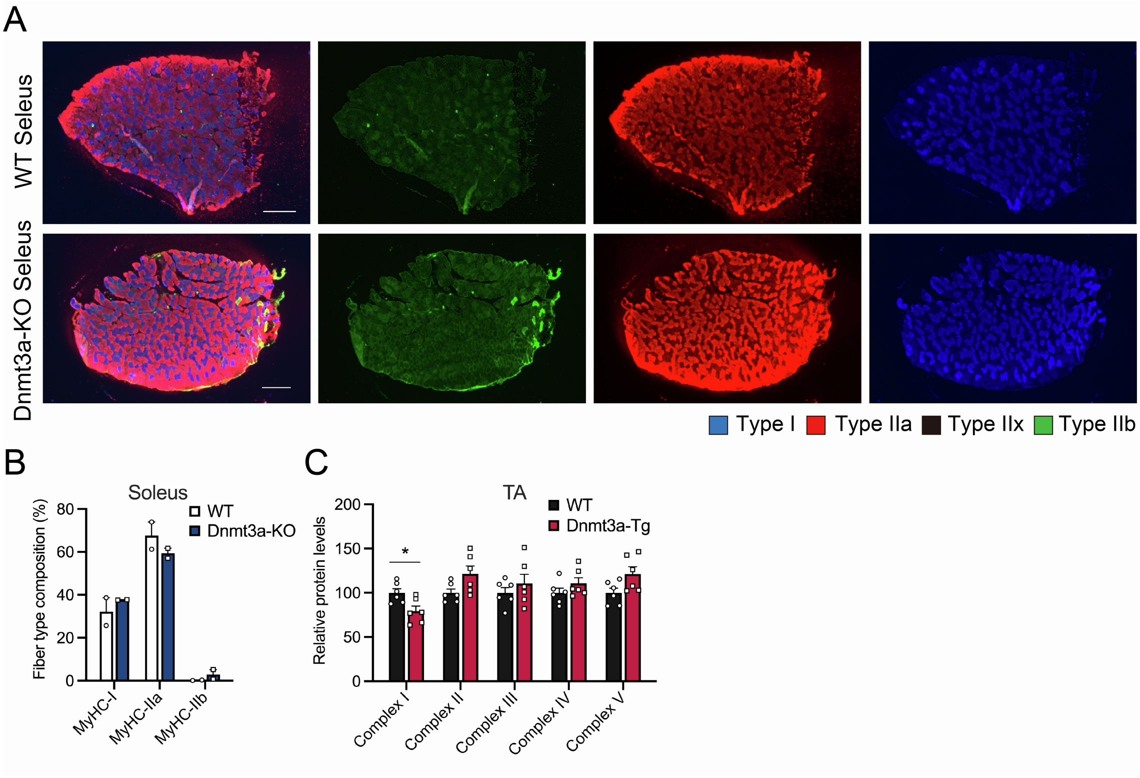

Figure S5. Skeletal muscle-specific deletion of Dnmt3a does not affect muscle fiber type

(A) Representative images of immunohistochemical staining of the myosin heavy chains and laminin (type I, blue; type IIa, red; type IIb, green) in soleus muscle cross-section of WT and skeletal muscle-specific Dnmt3a-KO mice. The unstained fibers are considered as type IIx fibers (black). Scale bar = 300 μm. (B) Frequency of each fiber type in the soleus muscle from WT and skeletal muscle-specific Dnmt3a-KO mice (n = 2 mice/ group). (C) Densitometric analysis of OXPHOS complex protein levels in the TA muscle of young (3-month-old) WT and Dnmt3a-Tg mice (n = 6 mice/group). All data indicate mean ± SE. * $P < 0.05$ (B, C) Student's two-tailed unpaired *t*-test.

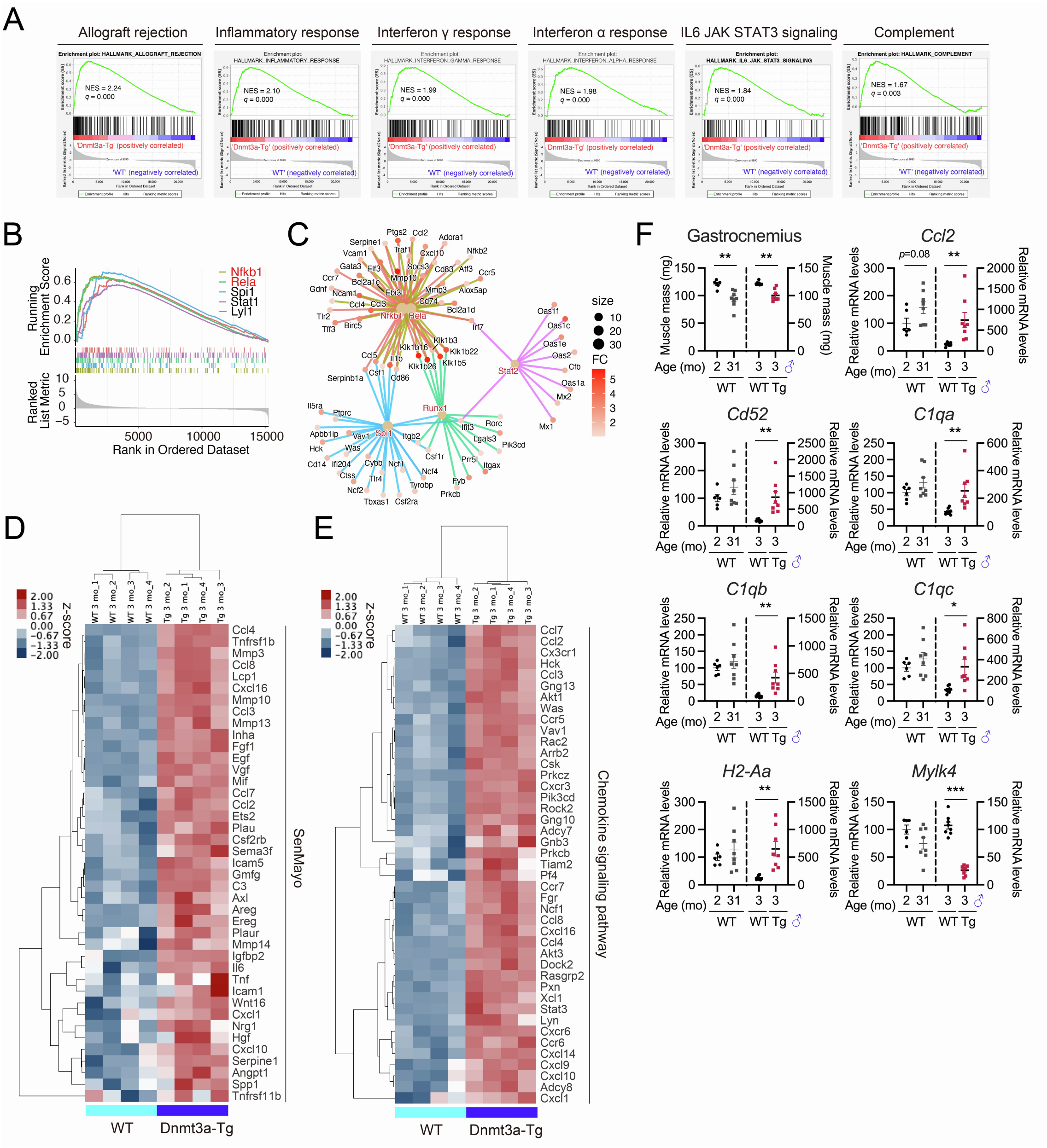

Figure S6. Increased DNA methylation in skeletal muscle by Dnmt3a results in increased inflammatory signaling

(A) GSEA of Dnmt3a-regulated genes from microarray data of young (3-month-old) WT and Dnmt3a-Tg muscles (n = 4 mice/group) using gene sets of the Molecular Signatures Database (MSigDB) "hallmark gene sets." (B) GSEA using DoRothEA regulon gene sets, showing the putative central transcription factors that are positively regulated in Dnmt3-Tg muscle. (C) Cnet plots of the results in figure 3G. (D, E) Hierarchical clustering of core gene sets associated with senescence (SenMayo) (D) and Chemokine signaling pathway (E) in young (3-month-old) Dnmt3a-Tg and WT muscles. (F) Gastrocnemius muscle mass and relative mRNA expression of chemokine, complement and AR target genes in gastrocnemius muscle from 3-month-old male

WT and Dnmt3a-Tg mice (n = 8 mice/group) and gastrocnemius muscle from young (3-month-old) and super-aged (31-month-old) male C57BL/6J mice (n = 6 young mice, n = 8 super-aged mice). All data indicate mean ± SE. **P* < 0.05, ***P* < 0.01, ****P* < 0.001. (F) Student's two-tailed unpaired *t*-test. WT: Wild type, Tg: Dnmt3a-Tg.

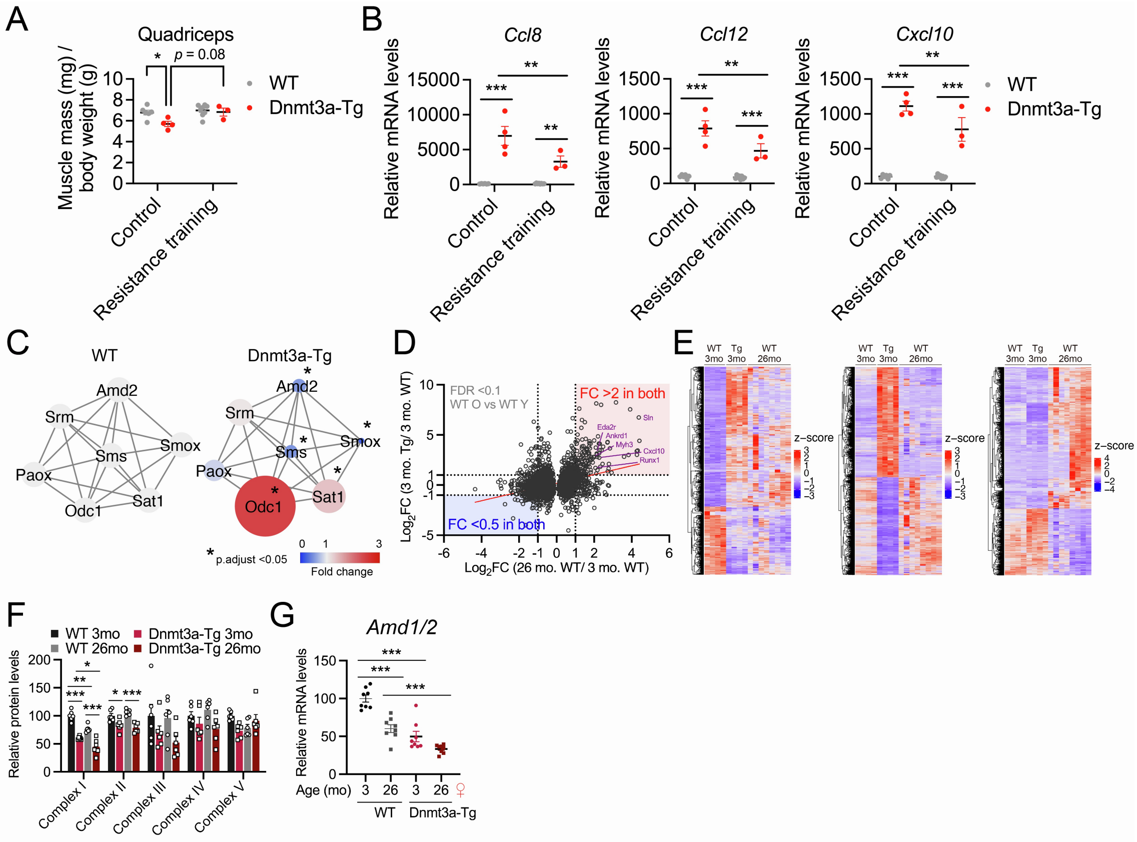

Figure S7. Increased DNA methylation in skeletal muscle by Dnmt3a recapitulates some of transcriptomic feature in skeletal muscle of elderly mice

(A) Weights of quadriceps muscles normalized to body weight in WT and Dnmt3a-Tg mice trained or untrained with resistance exercise (n = 3–8 mice/group). (B) Relative mRNA expression of *Ccl8*, *Ccl12*, and *Cxcl10* in in the gastrocnemius muscles of WT and Dnmt3a-Tg mice trained or untrained with resistance exercise (n = 3–8 mice/group). (C) PPI network from microarray data illustrating downregulation of some of the polyamine metabolism-related genes in Dnmt3a-Tg muscle compared with WT muscle. The color and circle size of nodes indicate fold-change values. (D) Scatter plot showing the genes that showed an alteration in the gastrocnemius muscle of young (3-month-old) Dnmt3a-Tg mice and older (26-month-old) WT mice (n = 4 in young mice and n = 8 in older mice, FDR < 0.1 in older WT versus young WT). The regression line is shown in red. (E) Heatmap analysis of differentially expressed genes (DEGs) in older (26-month-old) WT muscle with FDR < 0.1 and fold change value >1 compared to young (3-month-old) WT muscle. 3 conditions DEG analysis in young (3-month-old) and older (26-month-old) WT and age-matched Dnmt3a-Tg muscles were performed using RNAseqChef (3-month-old WT; n = 4, 3-month-old Dnmt3a-Tg; n = 4, 26-month-old WT; n = 8). (F) Densitometric analysis of OXPHOS complex protein levels in the TA muscle of young (3-month-old) and old (26-month-old) WT and Dnmt3a-Tg mice (n = 6 mice/group). (G) Relative mRNA expression of *Amd1/2* genes in gastrocnemius

muscle from young (3-month-old) and older (26-month-old) female WT and Dnmt3a-Tg mice (n = 8 mice/group). All data indicate mean ± SE. **P* < 0.05, ***P* < 0.01, ****P* < 0.001. (A, B, F, G) One-way analysis of variance (ANOVA) followed by Tukey's *post hoc* test. (C) Student's two-tailed unpaired *t*-test. WT: Wild type, Tg: Dnmt3a-Tg.

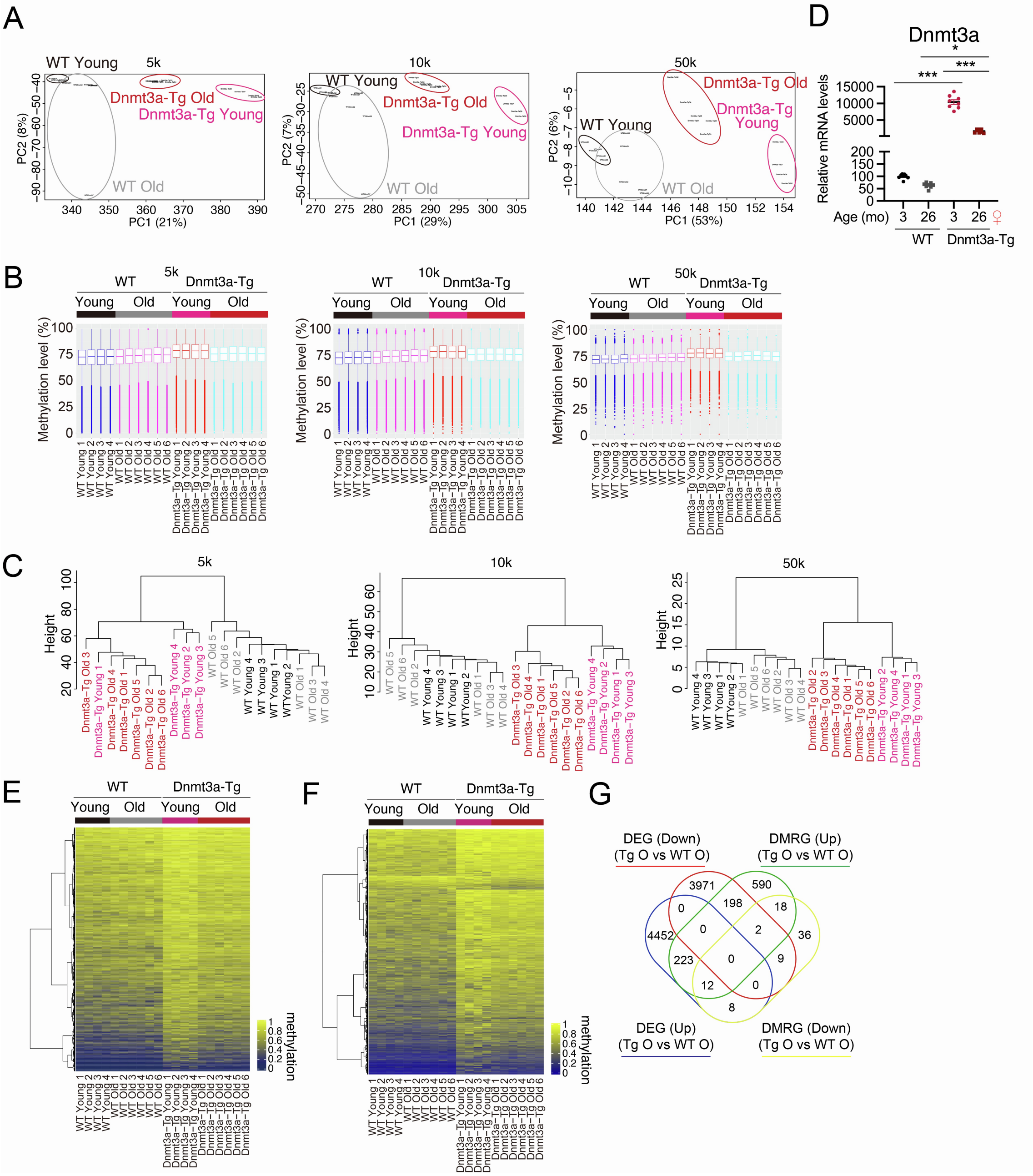

Figure S8. The effect of aging and Dnmt3a expression on skeletal muscle methylome

(A) Principal component analysis of DNA methylation of 5-kb, 10-kb, and 50-kb sidling windows. (B) Boxplot of mean CpG methylation level of 5-kb, 10-kb, and 50-kb sidling windows. (C) Unsupervised hierarchical clustering using Euclidean distance across the sample set. (D) Relative mRNA expression of Dnmt3a gene in gastrocnemius muscle from young (3-month-old) and older (26-month-old) female WT and Dnmt3a-Tg mice (n = 8 mice/group). (E) Heatmap showing DNA methylation levels in the Dnmt3a-associated hypermethylated regions (Dnmt3a-associated hyperDMR: hypermethylated regions in 3-month-old Dnmt3a-Tg muscle compared to 3-month-old WT muscle) in gastrocnemius muscle from WT and Dnmt3a-Tg mice (3-month-old WT, n = 4;

26-month-old WT, n = 6; 3-month-old Dnmt3a-Tg, n = 4; 26-month-old Dnmt3a-Tg, n = 6). (F) Heatmap showing DNA methylation levels in the Dnmt3a-associated hypermethylated regions in old age (Dnmt3a-associated hyperDMR: hypermethylated regions in 26-month-old Dnmt3a-Tg muscle compared to 26-month-old WT muscle) in gastrocnemius muscle from WT and Dnmt3a-Tg mice (3-month-old WT, n = 4; 26-month-old WT, n = 6; 3-month-old Dnmt3a-Tg, n = 4; 26-month-old Dnmt3a-Tg, n = 6). (G) Venn diagram showing that DMRGs by Dnmt3a expression in old age partially matched the differentially expressed genes (DEGs). All data indicate mean ± SE. *$P < 0.05$, ***$P < 0.001$. (D) Two-way analysis of variance (ANOVA) followed by Tukey's *post hoc* test. WT: Wild type, Tg: Dnmt3a-Tg, Y: Young, O: Old.

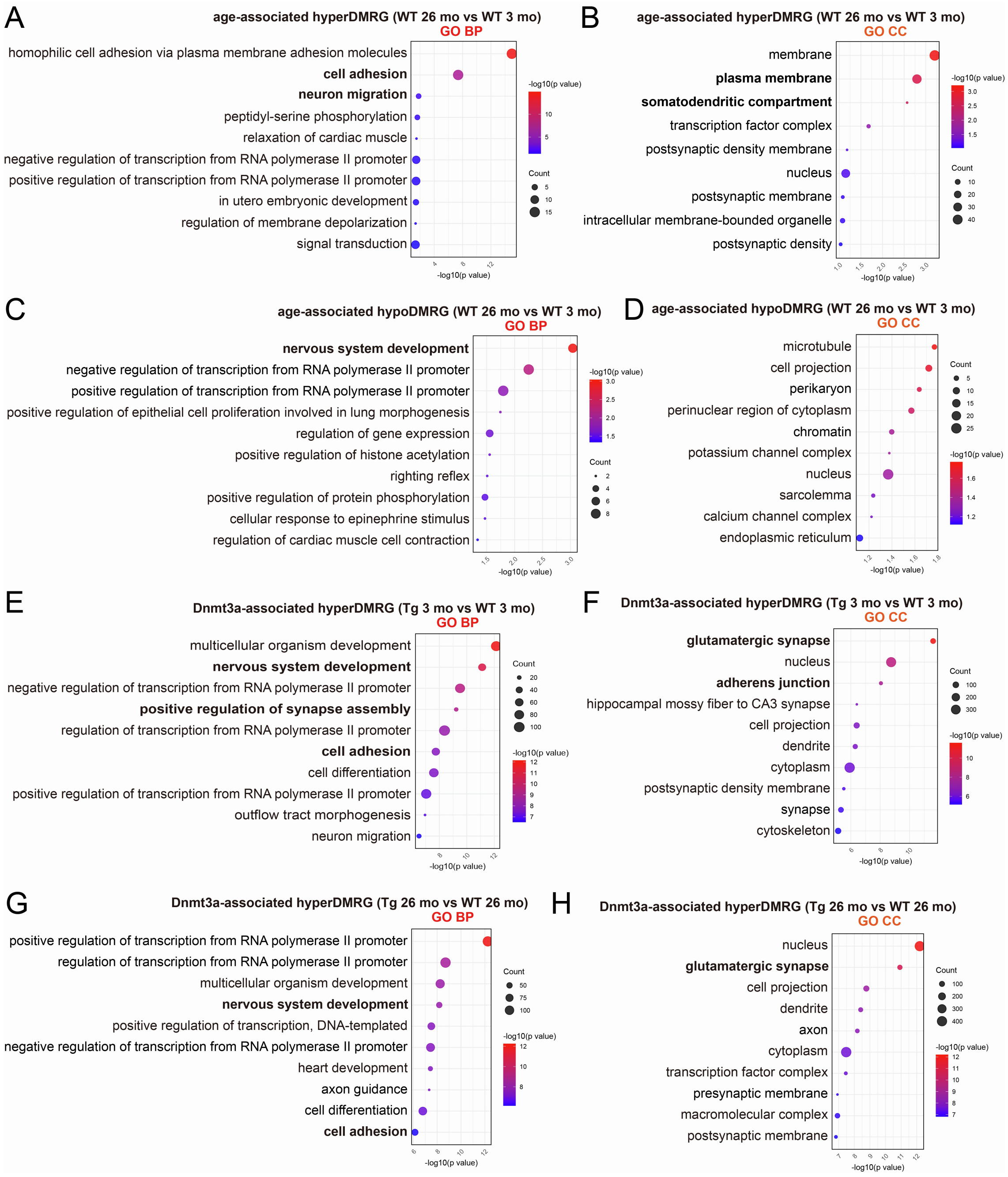

Figure S9. The GO analysis of DMRGs in skeletal muscle from WT and Dnmt3a-Tg mice

(A–H) GO enrichment analysis in biological process (A, C, E, G) and cellular component (B, D, F, H) of DMRGs in gastrocnemius muscle from WT and Dnmt3a-Tg mice. WT: Wild type, Tg: Dnmt3a-Tg.

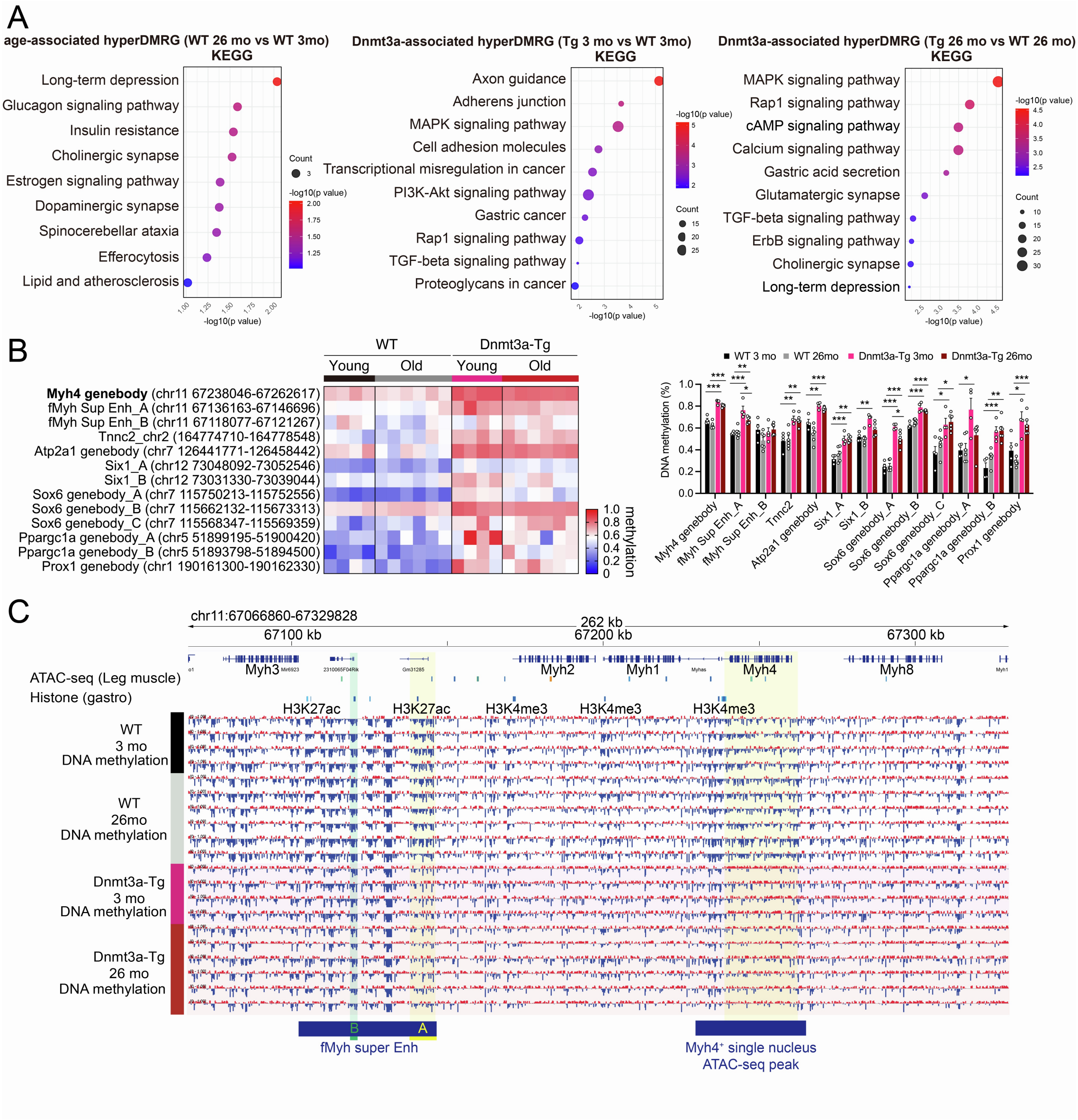

Figure S10. DNA methylation levels in the regions closest to the genes of interest in skeletal muscle from WT and Dnmt3a-Tg mice

(A) KEGG pathway analysis of DMRGs in gastrocnemius muscle from WT and Dnmt3a-Tg mice. (B) Heatmap and bar graph showing DNA methylation levels in the regions closest to the genes of interest in gastrocnemius muscle from WT and Dnmt3a-Tg mice. (C) DNA methylation levels around selected gene loci in the gastrocnemius muscle from young (3-month-old) and older (26-month-old) female WT and Dnmt3a-Tg mice (n = 8 mice/group). The mean value of 0.7205 was used to color the DNA methylation levels, with red and blue colors indicating hypermethylation and hypomethylation, respectively. Yellow indicates the regions of interest. The previously reported gastrocnemius histone ChIP-seq peaks and leg muscle ATAC-seq peaks were mapped with a significance threshold of 50 using the ChIP-Atlas. All data indicate mean ± SE. *P < 0.05,

**P < 0.01, ***P < 0.001. (B) Two-way analysis of variance (ANOVA) followed by Tukey's *post hoc* test. WT: Wild type, Tg: Dnmt3a-Tg.

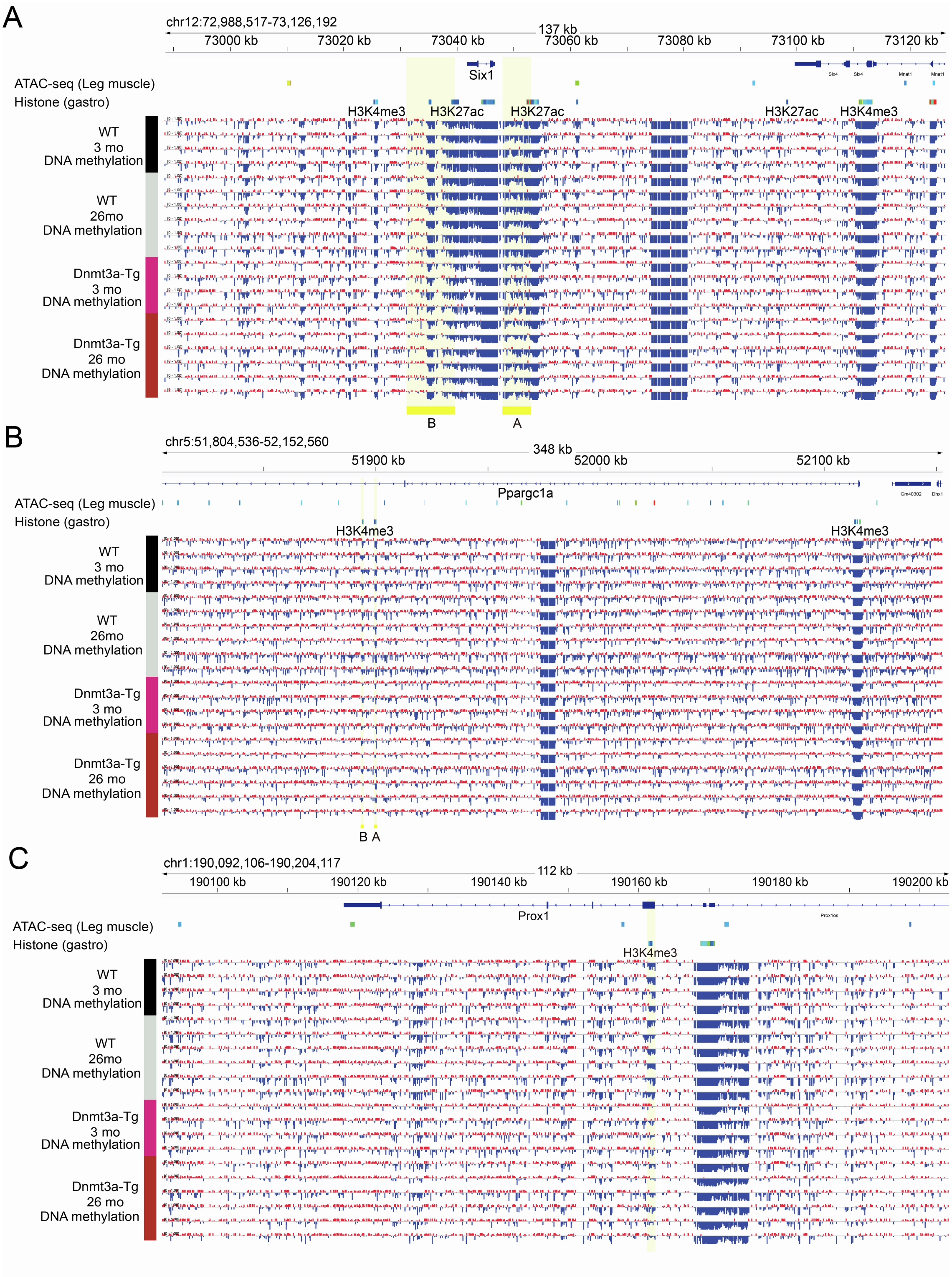

Figure S11. DNA methylation levels in the regions closest to the genes of interest in skeletal muscle from WT and Dnmt3a-Tg mice

(A–C) DNA methylation levels around selected gene loci in the gastrocnemius muscle from young (3-month-old) and older (26-month-old) female WT and Dnmt3a-Tg mice (n = 8 mice/group). The

mean value of 0.7205 was used to color the DNA methylation levels, with red and blue colors indicating hypermethylation and hypomethylation, respectively. Yellow indicates the regions of interest. The previously reported gastrocnemius histone ChIP-seq peaks and leg muscle ATAC-seq peaks were mapped with a significance threshold of 50 using the ChIP-Atlas. WT: Wild type, Tg: Dnmt3a-Tg.

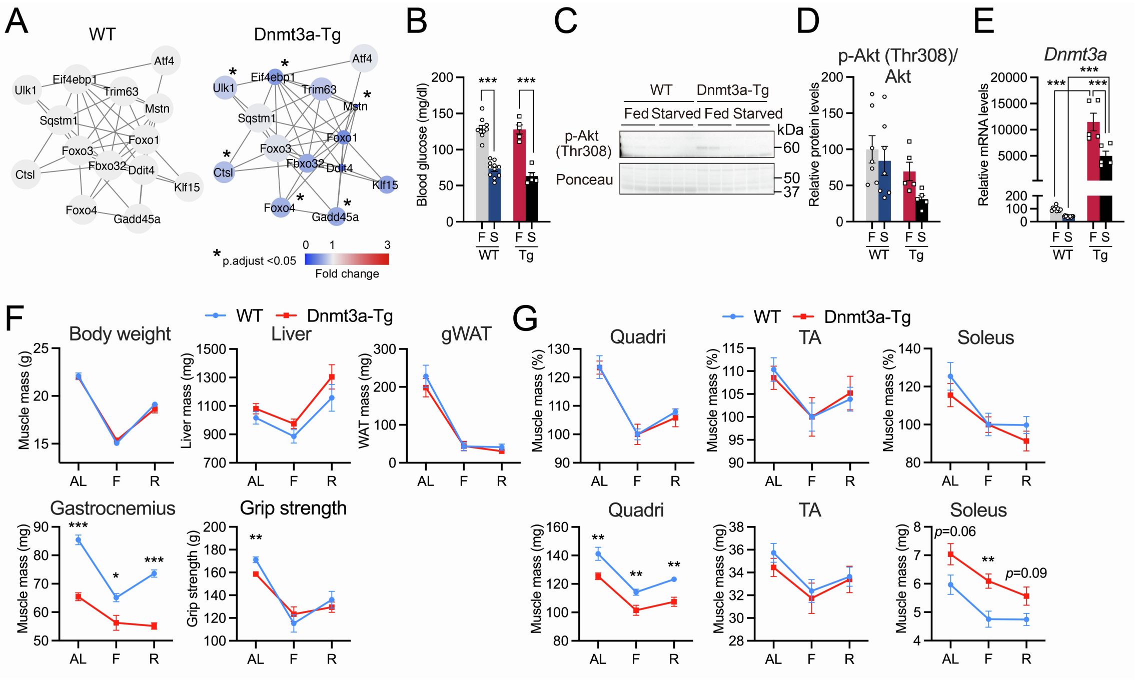

Figure S12. Dnmt3a-Tg mice were subjected to starvation

(A) PPI network of microarray data showing a set of FoxO signaling genes that were downregulated in Dnmt3a-Tg muscle compared with WT muscle. The color and circle size of nodes indicate fold-change values. (B) The blood glucose levels of fed and 48 h-fasted WT and 48 h-fasted WT and Dnmt3a-Tg female mice (n = 10-11 fed and 48 h-fasted WT mice, n = 5 fed and 48 h-fasted Dnmt3a-Tg mice). (C, D) Representative immunoblot images (C) and densitometric analysis (D) of p-Akt (Thr308) protein in the gastrocnemius of fed and 48 h-fasted WT and Dnmt3a-Tg female mice. (E) Relative mRNA expression of the *Dnmt3a* in gastrocnemius muscle from WT and Dnmt3a-Tg mice (n = 10-11 fed and 48 h-fasted WT mice, n = 5 fed and 48 h-fasted Dnmt3a-Tg mice). (F) The weight change of body weight, liver mass, gonadal white adipose tissue (gWAT) mass, gastrocnemius muscle mass, and grip strength in one AL-F-R cycle. (G) The weight change and the relative values of quadriceps muscle mass, TA muscle mass, and soleus muscle mass in one AL-F-R cycle. The relative values are normalized to the fasting state of WT and Dnmt3a-Tg mice, respectively. All data indicate mean ± SE. **P* < 0.05, ***P* < 0.01, ****P* < 0.001. AL: *ad libitum*, F: Fasting, R: Refeeding, WT: Wild type, Tg: Dnmt3a-Tg. (B, D, E) Two-way analysis of variance (ANOVA) followed by Tukey's post hoc test. (F, G) Student's two-tailed unpaired *t*-test.

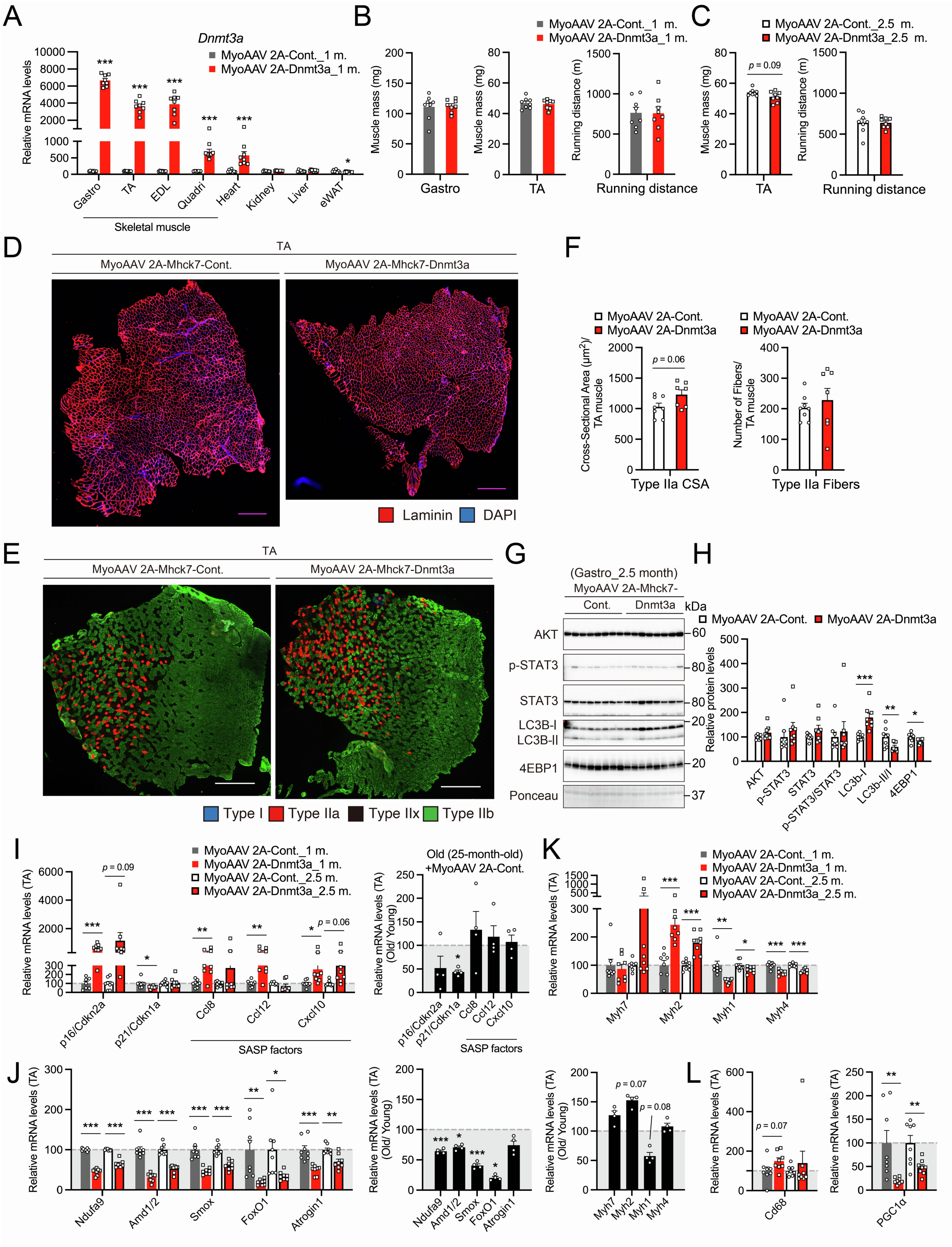

Figure S13. Dnmt3a overexpression in adult postnatal myofibers disrupts skeletal muscle homeostasis

(A) Relative mRNA expression of Dnmt3a in in the skeletal muscle and other tissues of MyoAAV-Mhck7-empty- or MyoAAV-Mhck7-Dnmt3a-treated mice (n = 8 mice/group) (1 months after AAV injection). (B, C) Weights of skeletal muscles and total running distance in a treadmill running test

from MyoAAV-Mhck7-empty- or MyoAAV-Mhck7-Dnmt3a-treated mice (n = 8 mice/group) (1 or 2.5 months after AAV injection). (D) Representative images of immunohistochemical staining of laminin and DAPI (laminin, red and DAPI, blue) in TA muscle cross-section from MyoAAV-Mhck7-empty- or MyoAAV-Mhck7-Dnmt3a-treated mice (2.5 months after AAV injection). Scale bar = 500 μm. (E) Representative images of immunohistochemical staining of myosin heavy chains (type I, blue; type IIa, red; type IIb, green) in TA muscle cross-section from MyoAAV-Mhck7-empty- or MyoAAV-Mhck7-Dnmt3a-treated mice (2.5 months after AAV injection). Scale bar = 500 μm. (F) Quantification of type IIa fiber cross-sectional area (CSA) (left) and type IIa fiber number in the TA section (n = 7–8 mice/group) (2.5 months after AAV injection). (G, H) Immunoblot images (G) and densitometric analysis (H) of AKT, p-STAT3, STAT3, LC3b, and 4EBP1 proteins in the gastrocnemius muscles of MyoAAV-Mhck7-empty- or MyoAAV-Mhck7-Dnmt3a-treated mice (n = 8 mice/group) (2.5 months after AAV injection). (I–L) Relative mRNA expression of genes related to senescence and SASP factors (I), mitochondrial complex I, polyamine metabolism, and FoxO1 signaling (J), myosin heavy chain (K), and *Cd68* and *PGC1α* (L) in the TA muscles of MyoAAV-Mhck7-empty- or MyoAAV-Mhck7-Dnmt3a-treated mice (n = 8 mice/group) (1 or 2.5 months after AAV injection). The black bar shows the relative mRNA expression in the TA muscles of old (25-month-old) mice treated with MyoAAV-Mhck7-empty compared to young (3-month-old) mice treated with MyoAAV-Mhck7-empty (n = 4 mice/group). All data indicate mean ± SE. *$P < 0.05$, **$P < 0.01$, ***$P < 0.001$. Student's two-tailed unpaired *t*-test.

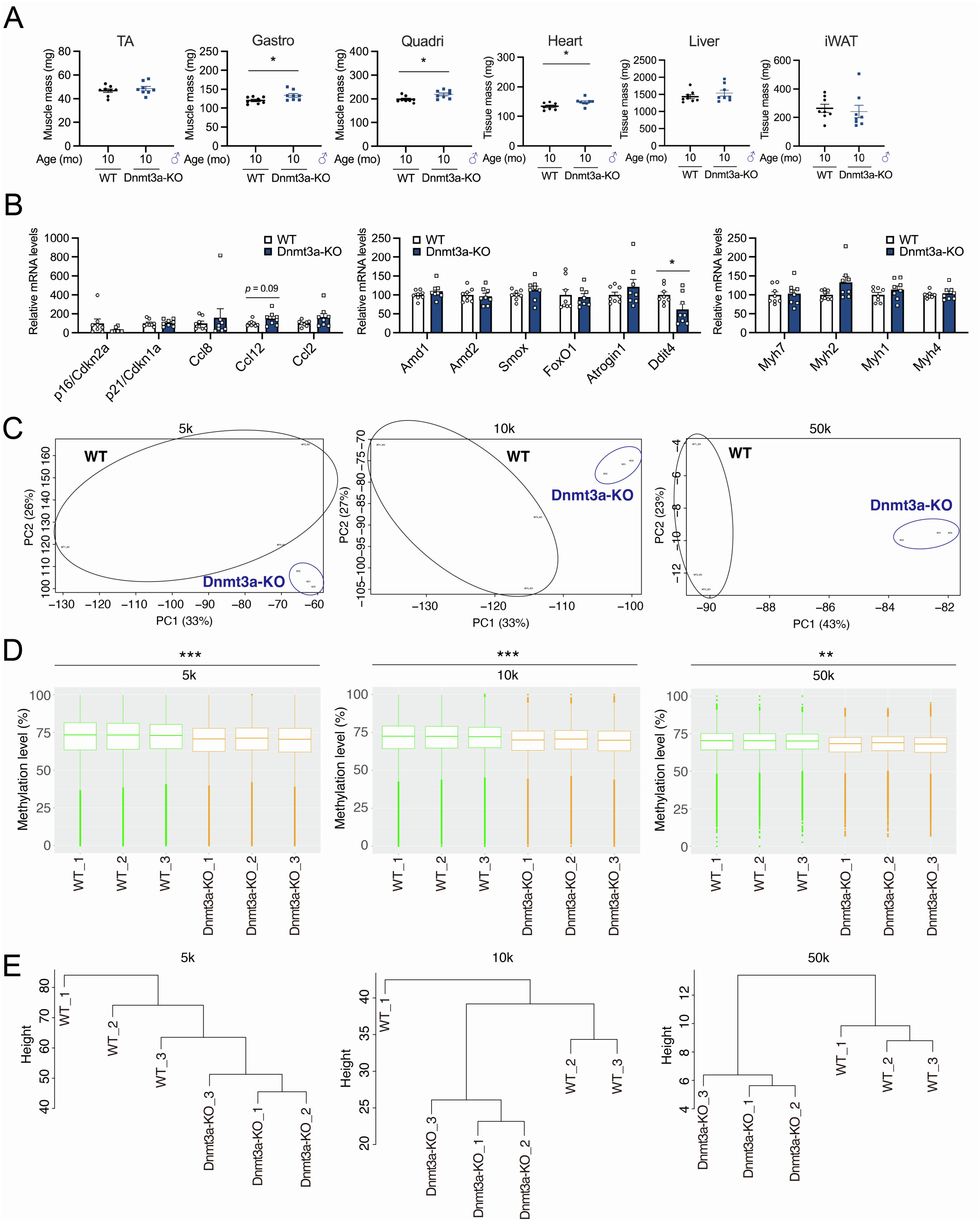

Figure S14. Knockout of Dnmt3a in muscle dose not reduce muscle mass

(A) Weights of skeletal muscles and other tissues from WT and Dnmt3a-KO mice (n = 8 mice/group). (B) Relative mRNA expression of genes related to senescence, SASP factors, mitochondrial complex I, polyamine metabolism, and FoxO1 signaling, and myosin heavy chain in the gastrocnemius muscles of WT and Dnmt3a-KO mice (n = 8 mice/group) (C) Principal component analysis of DNA methylation of 5-kb, 10-kb, and 50-kb sidling windows (n = 3

mice/group). (D) Boxplot of mean CpG methylation level of 5-kb, 10-kb, and 50-kb sidling windows (n = 3 mice/group). (E) Unsupervised hierarchical clustering using Euclidean distance across the sample set (n = 3 mice/group). All data indicate mean ± SE. *$P < 0.05$, **$P < 0.01$, ***$P < 0.001$. Student's two-tailed unpaired *t*-test.

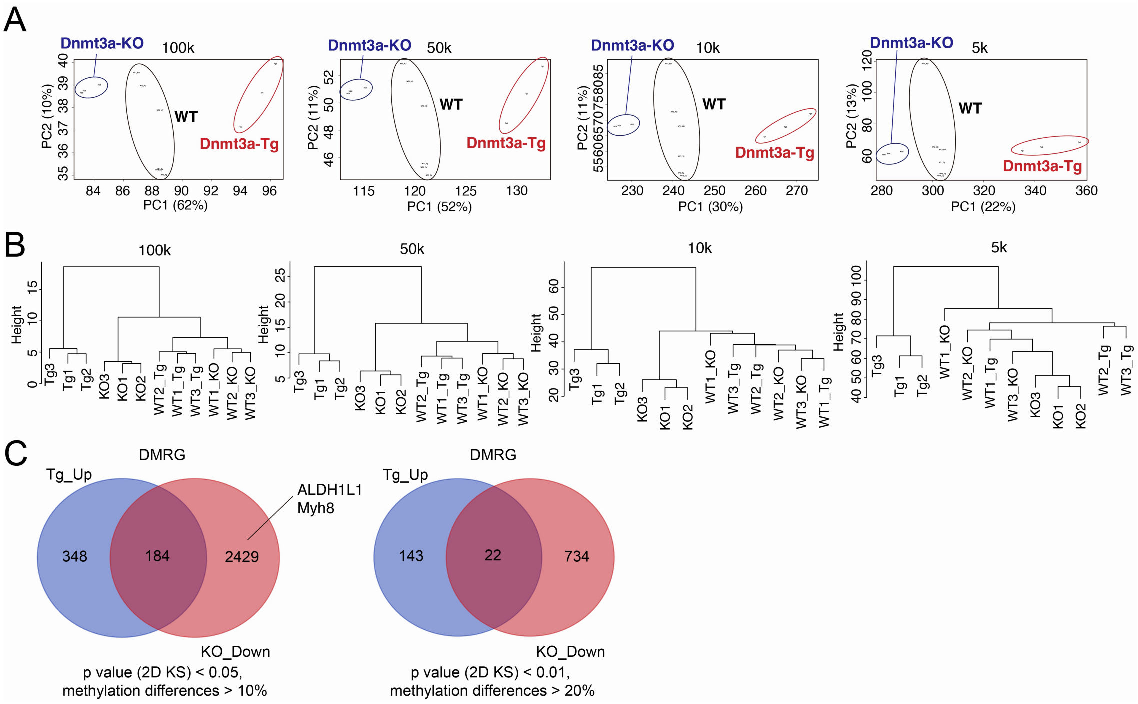

Figure S15. Comparison of DNA methylation in Dnmt3a-Tg muscle and Dnmt3a-KO muscle

(A) Principal component analysis of DNA methylation of 5-kb, 10-kb, 50-kb, and 100-kb sidling windows (n = 3 mice/group). (B) Unsupervised hierarchical clustering using Euclidean distance across the sample set (n = 3 mice/group). (C) Venn diagram showing that overlap of DMRGs increased in Dnmt3a-Tg muscle and decreased in Dnmt3a-KO muscle.

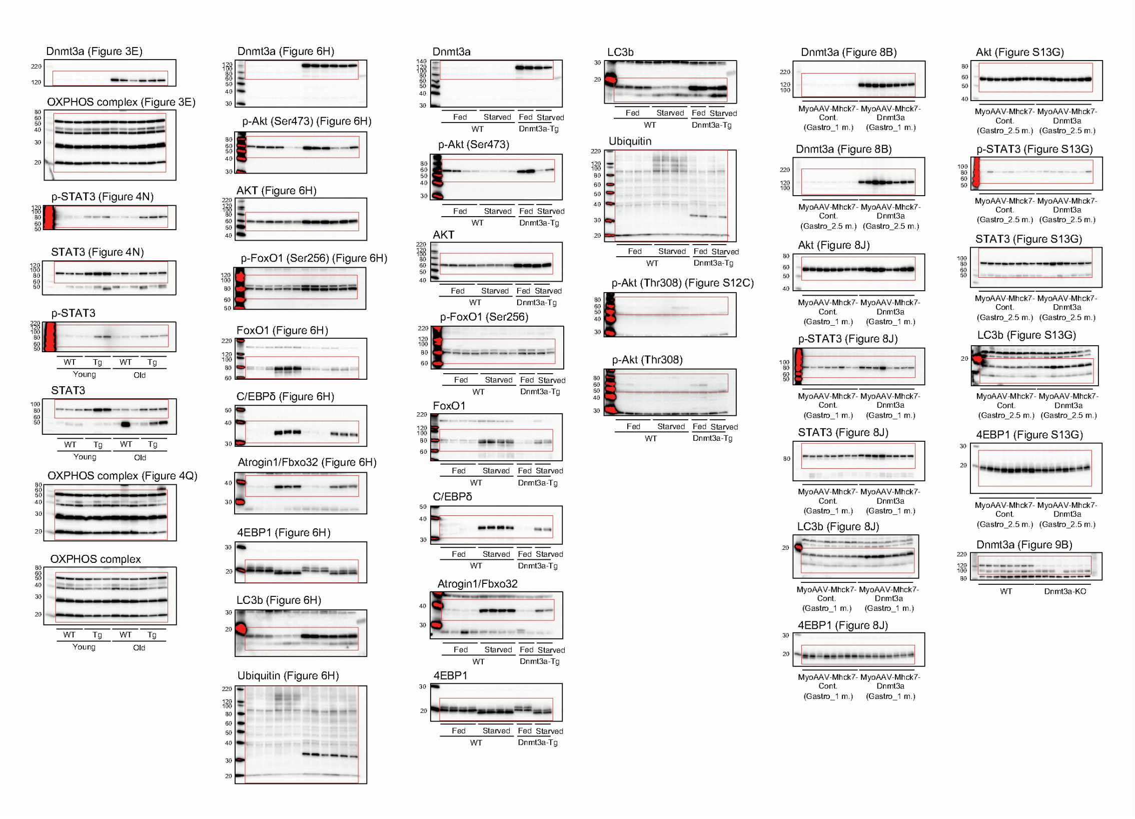

Figure S16. Original western blot images

Table S9. List of PCR primer sequences used for genotyping

Gene	Forward Primer (5'-3')	Reverse Primer (5'-3')
HSA	ATGGTTGGGGAGGCCTTTGG	GGAAGCGAGGCTTCACTTGG
Cre	CGCCGCATAACCAGTGAAAC	ATGTCCAATTTACTGACCG

Table S10. List of mouse-specific primer sequences used for real-time PCR

Gene	Forward Primer (5'-3')	Reverse Primer (5'-3')
36B4	GGCCCTGCACTCTCGCTTTC	TGCCAGGCAGCGCTTGT
18S	GGGAGCCTGAGAAACGGC	GGGTCGGGAGTGGGTAATTTT
β-actin	CTGGCTCCTAGCACCATGAA	GCAGCTCAGTAACAGTCCGC
Dnmt3a	AATGACCTCTCCATTGTCAA	TAACGCCCATGGCCACCACA
Myh7	GATGTTTTTGTGCCCGATGA	CAGTCACCGTCTTGCCATTCT
Tnnc1	TTGTCATGATGGTTCGGTGC	GTAGCCATCAGCGTTTTTGTCA
Tnni1	CATGCTGAAGAGCCTGATGCT	GAATGCGCTCCGAGAGGTAA
Tnnt1	GGTCAAGGCAGAACAGAAGC	GCGGTTGTAGAGCACATTGA
Tpm3	TGCTGATGAGAGTGAGAGAGGTATG	CCTTTAGCTGGATTTCCTGGAGTT
Mybpc1	ATCCAGATTGTTGACCGTCCA	AGCATTCCCATCGTCCTTTG
Atp2a2	GCCCCCCTGGGAGAATATC	TCTGGAAAATGAGCGGCAA
Myh4	CACCTGGACGATGCTCTCAGA	GCTCTTGCTCGGCCACTCT
Myh1	CGGAGTCAGGTGAATACTCACG	GAGCATGAGCTAAGGCACTCT
Myh2	AAGCGAAGAGTAAGGCTGTC	GTGATTGCTTGCAAAGGAAC
Tnnc2	TTAAAGAGTTGGGCACCGTGA	CGATAGTACCGCTGCCATCC
Tnni2	CTCCATGTCTGAAGTGCAGGAA	GCTGCTCTTCTGCACCTTCAC
Tnnt3	AAGCCCAGAAGCACAGCAAG	GGGTTGATGGTCTCTGCTGC
Actn3	GACTATGCCAAGCTTCGCAAG	TCTGCATCCAGCATCTTAGGAAT
Atp2a1	GGAACGGAATGCAGAGAACG	CCCGAGCCTTGATCCTTTG
Six1	GGGAGAACACCGAAAACAATAACT	GCTGGACATGAGCGGCTT
Six2	CGAGCACCTCCACAAGAATGA	AACTGGTGGCTCTCCAGGATT
Mafa	TGAACCTCCTGGCATGGG	CGGCTAGTCCAAAAAAGCGA
Mafb	CAAGACAGGCTTTGCGTCCT	TCTTTTCACCCTAAGCCCTGG
Maf	GGCAATGAACAATTCCGACC	GGTCTCCACCGGTTCCTTTT
Sox6	TTGGGGAGTACAAGCAACTGATGC	ATCTGAGGTGATGGTGTGGTCGTT
Fnip1	GGCATAGCACGTTCTGCATCT	CAACGTCGCTGGTAGCTGC
Myogenin	CATGGTGCCCAGTGAATGCAACTC	TATCCTCCACCGTGATGCTGTCCA
Mef2d	GGAACCGCCAGGTGACC	GTTGAAGATGATGAGCGCGA
PGC1α	CGGAAATCATATCCAACCAG	TGAGGACCGCTAGCAAGTTTG

ERRγ	CATTGGATGGGCAAAACATATTC	TCTGCCAGGGACAGTGTGG
PPARδ	AGAAGTGCGATCGGATCTGC	GATAGCGTTGTGCGACATGC
Prox1	CACCTTATTCAGGAAGCGCAA	GGGTAGCGGGTGTAAAAGAACA
Neurturin	GGGCTACACGTCGGATGAG	CCAGGTCGTAGATGCGGATG
Ccl8	CAACATGAAGATCTACGCAGTGC	AGCAGGTGACTGGAGCCTTATC
Ccl12	GACCAGATGCGGTGAGCAC	GGGACACTGGCTGCTTGTG
Cxcl10	GGGATCCCTCTCGCAAGG	GGCTCGCAGGGATGATTTC
Cd68	CTTGGGAACTACACGTGGGC	CCAAGCCTTTCTTCCACCCT
Eda2r	TCCCCTCTACTGGACCTGAA	TGAAAGAGACCTTTCTAGTTCACCT
Tlr4	CTCTGCCTTCACTACAGAGAC	TGGATGATGTTGGCAGCAATG
Ncam1	TTCAAGCAGACACACCGTCTTC	GCACGCCCCCTGTGG
Runx1	ACGATGAAAACTACTCGGCAG	CTGAGGTCGTTGAATCTCGCT
Chrnd	CAGCCAAGGAGATCACACTTAGC	GTTCTCTGTGAAACCTTCAGGGTC
Cdkn1a/p21	GTCTGAGCGGCCTGAAGAT	TCTGCGCTTGGAGTGATAGA
AR	CCAGATGGCGGTCATTCAGT	GCATGCGGTACTCATTGAAAAC
Amd1/2	TTTCACCGTGGCCTTCAGAT	GCCTCCTCCCTCTGGTCAGT
Smox	CACAGAGAGCTCCAAGACAGC	GGGCACTTGGATGGTAAAAG
Cdkn2a/p16	TGTTGAGGCTAGAGAGGATCTTG	CGAATCTGCACCGTAGTTGAGC
Ndufa9	GAGGCCGCTCATCTGTCAGT	TTGTGACCCCATTCGTCCA
Akt1	GCCCAACACCTTTATCATCC	GTCCATCGTCTCTTCTTCCTG
Atrogin1	TCGGCAAGTCTGTGCTGGT	CCATCCGATACACCCACATGT
Trim63/MuRF1	GCTGGTGGAAAACATCATTGACAT	CATCGGGTGGCTGCCTTT
p62	TCTGGGGTAGTGGGTGTCAG	AGAATGTGGGGGAGAGTGTG
Ulk1	TGAAGCAGGTGGTACGCAGA	GCCGTTGTTTGTCCAGAAAGA
CathepsinL	TCTCACGCTCAAGGCAATCA	AAGCAAAATCCATCAGGCCTC
4EBP1	GGCGGCACGCTCTTCA	TCCGACACTCCATCAGAAATTTC
Gadd45a	CGTAGACCCCGATAACGTGGTA	CGGATGAGGGTGAAATGGAT
Myostatin	AGTGGATCTAAATGAGGGCAGT	GGAGTACCTCGTGTTTTGTCTC
Ddit4/Redd1	AGCCAGTCCCTGACGCTAAG	CGGAGCTGTAGAGTTTCTTCTTGA
C/EBPδ	TTCAGCGCCTACATTGACTC	GCTTTGTGGTTGCTGTTGAAG
KLF15	TGCAGCAAGATGTACACCAAGA	GGTGCCTTGACAACTCATCTGA
ATF4	CCTGAACAGCGAAGTGTTGG	TGGAGAACCCATGAGGTTTCAA

FoxO1	GCGGGCTGGAAGAATTCAAT	TCCAGTTCCTTCATTCTGCA
FoxO3a	TCTGCGGGCTGGAAGAACT	CTCTTGCCCGTGCCTTCAT
FoxO4	ATGGATGGTCCGCACGGTG	CTTGCCAGTGGCCTCGTTG
LC3b	GCTTGCAGCTCAATGCTAAC	CCTGCGAGGCATAAACCATGT
Depp1	TGAGCACTCTCTGGGAAGAAAAC	GATCACTGGGAGGTGCAAATAGA
Pdk4	GTGAACACTCCTTCGGTGCAG	CAGGCTCTGGATATACCAGCTCTT
Chac1	GCCCTGTGGATTTTCGGGTA	ATCTTGTCGCTGCCCCTATG
Angptl4	CTCTCTGGTGGCTGGTGGTT	AGATACCCTTTTTACGCTCCTGC
Arrdc3	AAGGGCTACACCCCAGGTG	AGGCCTGCGTCTGGTAAATG
Slc43a1	CCCTGAATGAGAATGCTTCCTT	ATGGCATTGGTGAGCTTTTGT
Arrdc2	CGAGGAGGACCCTAACCCA	GCCAAAGAGTGTGAACCTGGTAC
COX2 (mitochondrial)	CCGACTAAATCAAGCAACAGTAACA	AAATTTCAGAGCATTGGCCATAG
COX4 (nuclear)	CTATGTGTATGGCCCCATCC	AGCGGGCTCTCACTTCTTC
Ccl2	CCAGCTCTCTCTTCCTCCACC	TTAACTGCATCTGGCTGAGCC
Cd52	TGGTTGTGATTCAGATACAAACAGG	TGGATGAGGCCCCACTCTT
C1qa	CAGGAGCTGCTGGCATCC	TGGGACCTGGGAGCCC
C1qb	ATTCCATACACAGGAAGCCCC	CCAGGACACATGGAGAAAACCTA
C1qc	CCTCAGGATGGTCGTTGGAC	AGTGGTAGGGCCAGAAGAAACA
H2-Aa	CACCTTCATCCCTTCTGACGAT	GGGCTGGAATCTCAGGTTCC
Mylk4	TGGAGGTCCCACTGGATGA	GCTCACGGTGTACAGATTGTCC
Aldh1l1	TACATCGCTAAGGAGGAGTC	GCCTGCAGTTTGTCACTGAC
Myh8	CGGGAGGTTCACACCAAAATCA	CAGAGAGGCAAGTGACCCAGCA
Amd1	GCTTTCGTCCCCATTAGATGC	CCCTAGAAAGTGGAGGTGCCTG
Amd2	GCTTTCGTCCCCATTAGATGC	CCCTAGAAAGTGGAGGTAACTG
